# An updated checklist of mosquitoes (Diptera, Culicidae) of Ecuador: new records and public health significance

**DOI:** 10.3897/zookeys.1272.179156

**Published:** 2026-03-05

**Authors:** Patricio Ponce, Varsovia Cevallos, Andrés Carrazco-Montalvo, Jennifer Gallardo-Cóndor, Valentina Arévalo, Ximena Galarza, Josefina Coloma

**Affiliations:** 1 Centro de Investigación en Enfermedades Infecciosas y Vectoriales (CIREV), Instituto Nacional de Investigación en Salud Pública, Quito, Ecuador Universidad Técnica de Cotopaxi, Facultad de Ciencias Agropecuarias y Recursos Naturales Latacunga Ecuador; 2 Centro de Referencia Nacional de Genómica, Secuenciación y Bioinformática (CRN-GENSBIO), Instituto Nacional de Investigación en Salud Pública, Quito, Ecuador School of Public Health, University of California Berkeley United States of America; 3 Universidad Técnica de Cotopaxi, Facultad de Ciencias Agropecuarias y Recursos Naturales, Carrera de Biotecnología, Latacunga, Ecuador Centro de Investigación en Enfermedades Infecciosas y Vectoriales (CIREV), Instituto Nacional de Investigación en Salud Pública Quito Ecuador; 4 Division of Infectious Diseases and Vaccinology, School of Public Health, University of California, Berkeley, CA 94704, USA Centro de Referencia Nacional de Genómica, Secuenciación y Bioinformática (CRN-GENSBIO), Instituto Nacional de Investigación en Salud Pública Quito Ecuador

**Keywords:** Culicidae, diseases, diversity, DNA barcoding, taxonomy, vector-borne

## Abstract

Mosquitoes are major vectors of human and animal diseases, making their accurate identification essential for vector surveillance and control. However, morphological identification has often been challenging, requiring taxonomic expertise and well-preserved specimens. Molecular markers, particularly DNA barcoding, offer an effective alternative for identifying both adult and immature stages. Ecuador is one of the most biodiverse countries in the world, a diversity that is also evident in its Culicidae fauna. This study provides a comprehensive revision of Ecuadorian mosquitoes, updating the national checklist and emphasizing species of public health importance. For species identification, an integrative approach was used combining morphology and DNA barcoding (COI and ITS2 regions). We list 266 species in 22 genera, of which 17 species are new national records, and 33 species are validated through molecular analysis. The updated checklist highlights Ecuador’s Culicidae diversity across its biogeographic regions, which represent 7% of the world’s mosquito diversity. These findings provide a critical foundation for future entomological research and vector control in the country.

## Introduction

The family Culicidae is one of the most widely distributed families in tropical and subtropical regions worldwide. It exhibits significant taxonomic diversity, encompassing two subfamilies, Anophelinae and Culicinae, with 112 genera and a total of 3,718 species officially recognized as of May 2025 (Beebe 2018; Harbach 2025). Mosquitoes are important vectors of animal and human diseases, with significant economic and public health implications (Nebbak et al. 2022). Their vast ability to transmit infectious and potentially deadly pathogens and parasites makes them the most significant blood-feeding arthropods globally (Jawień et al. 2024). Consequently, entomological and epidemiological monitoring is required to guide vector control strategies aimed at preventing and reducing the disease burden (Mojica et al. 2025). However, effective mosquito control depends on understanding species-specific biological traits, making accurate identification essential for managing vector-borne diseases (Ashfaq et al. 2014).

Mosquito classification based on adult and larval morphological characteristics is time-consuming and requires extensive taxonomic knowledge (Taira et al. 2012; Hernández-Triana et al. 2019). Taxonomic keys are intricate and present various limitations, while the reliability of morphological identification depends on maintaining sample integrity, which is not always achievable in field settings (Telles-de-Deus et al. 2024). Consequently, complementary tools for species morphological identification, based on molecular markers—short DNA sequences commonly located in mitochondrial and ribosomal genomes—provide a valuable alternative for classifying both adult and immature mosquito stages (Chakraborty et al. 2021).

Various molecular markers such as Cytochrome c oxidase subunit 1 (COI), Cytochrome b (Cytb), NADH dehydrogenase subunit 4 (ND4), and internal transcribed spacer 2 (ITS2), have been employed in mosquito barcoding studies (Hebert et al. 2003; Yao et al. 2010; Bourke et al. 2013; Foster et al. 2013). However, the COI DNA barcode region is considered a universal marker for identifying animal species and remains a key tool for assessing the genetic diversity and structure of mosquitoes (Diptera: Culicidae) (Laurito et al. 2013, 2022; Kirchgatter et al. 2020).

Ecuador is located on the northwest coast of South America and comprises both the continental mainland and the Galápagos Islands. The continental territory has the coastal Pacific lowlands, the Andean highlands, and the Amazonian lowlands, resulting in 26 Holdridge ecological life zones, among the highest in the Neotropics (Holdridge et al. 1971; Cañadas 1983). This remarkable geographical diversity has made Ecuador one of the world’s most biodiverse countries, a richness also reflected in the Culicidae family. Despite its long-standing status as a hotspot for mosquito-borne diseases, research on this group has been largely neglected for the past 50 years (Sippy et al. 2020). As a result, limited studies have left gaps in our understanding of mosquito diversity and disease dynamics. Meanwhile, vector mosquitoes continue to expand their global distribution, further increasing the risk of human and animal diseases (Tatem et al. 2012; Mier-y-Teran-Romero et al. 2017).

The most recent comprehensive checklist documented 179 species in Ecuador (Linton et al. 2013) predominantly within the genera *Anopheles*, *Aedes*, and *Culex*, which include taxa of medical significance and several species endemic to the country. This study updates the national inventory of mosquito species of Ecuador—emphasizing key pathogen vectors — through an integrative taxonomy approach. Morphological identification utilized taxonomic keys, original descriptions, and revisions, complemented by DNA barcoding (COI and ITS2 regions) for 33 species from 91 specimens. Historical records from scientific literature, catalogs, databases, and collections were systematically reviewed, resulting in 266 species across 22 genera, including 17 new national records.

## Materials and methods

Adult mosquitoes were collected using a variety of sampling methods—including CDC traps, BG-Sentinel traps, motor-driven aspiration, manual aspiration (adults), manual collection (immatures), human/animal bait, rotary traps, and resting traps—between 2009 and 2025. Sampling was conducted under standardized entomological protocols established for each investigation project, ensuring comparability across sites and years. These efforts covered diverse natural habitats in Ecuador’s Coastal, Andean, Amazon, and Insular regions, encompassing both undisturbed and anthropized areas.

The mosquito specimens are preserved in the National Collection of Arthropod Vectors (CIREV) at INSPI-Quito (Instituto Nacional de Investigación en Salud Pública). The nomenclature used in this study follows the accepted species names established by Harbach and Kitching (1998) and the abbreviations for genera and subgenera conform to the conventions proposed by Reinert (Reinert 2001, 2009).

Species identification was performed using taxonomic literature, incorporating original species descriptions and subsequent taxonomic revisions. Additionally, molecular identification using COI marker confirmed 30 species: six *Aedes*, seven *Anopheles*, 11 *Culex*, two *Limatus*, one *Mansonia*, one *Psorophora*, and two *Sabethes*. Molecular identification using ITS2 marker confirmed three species: one *Psorophora* and two *Coquillettidia*. COI gene fragments from 91 specimens were sequenced using Sanger technology. In addition, three specimens underwent further sequencing with Illumina AmpliSeq due to the low quality of their initial Sanger-generated sequences. For these runs, ITS2 primers ITS2_5.8-F (5’-TGTGAACTGCATGACACAT-3’), ITS2_A-F (5’-TGTGAACTGCAGGACACAT-3’) and ITS2_28-R (5’-ATGCTTAAATTTAGGGGGTA-3’) (Paskewitz and Collins 1990; Beebe and Saul 1995) were included. PCR amplifications for the COI gene were initially performed using the universal primers LCO1490-F (5’-GGTCAACAAATCATAAAGATATTGG-3’) and HCO2198-R (5’-TAAACTTCAGGGTGACCAAAAAATCA-3’) (Folmer et al. 1994). In cases where these primers yielded sequences of poor quality, an alternative primer set, Kumar-F (5’-GGATTTGGAAATTGATTAGTTCCTT-3’) and Kumar-R (5’-AAAAATTTTAATTCCAGTTGGAACAGC-3’), was employed to improve sequence reliability (Kumar et al. 2007). Sequences obtained in this study were deposited in GenBank under BioProject PRJNA1400953, which contains all sequences generated.

To update the species checklist, an extensive literature review was conducted, including mosquito catalogs (Belkin 1962; Belkin et al. 1965a; Forattini 1965; Berlin 1969; Stone 1970; Arnell 1973; Gorham et al. 1973; Zavortink 1973, 1979; Knight and Stone 1977; Faran 1979, 1980; Heinemann and Belkin 1979; Berlin and Belkin 1980; Sirivanakarn and Galindo 1980; Faran and Linthicum 1981), recognized taxonomic databases (Bánki et al. 2025; GBIF-Secretariat 2025), reports (AFPMB 1998), and peer-reviewed scientific articles. All species were included with annotations indicating: (1) valid species, (2) availability of specimens, and (3) new country record.

**Figure 1.**
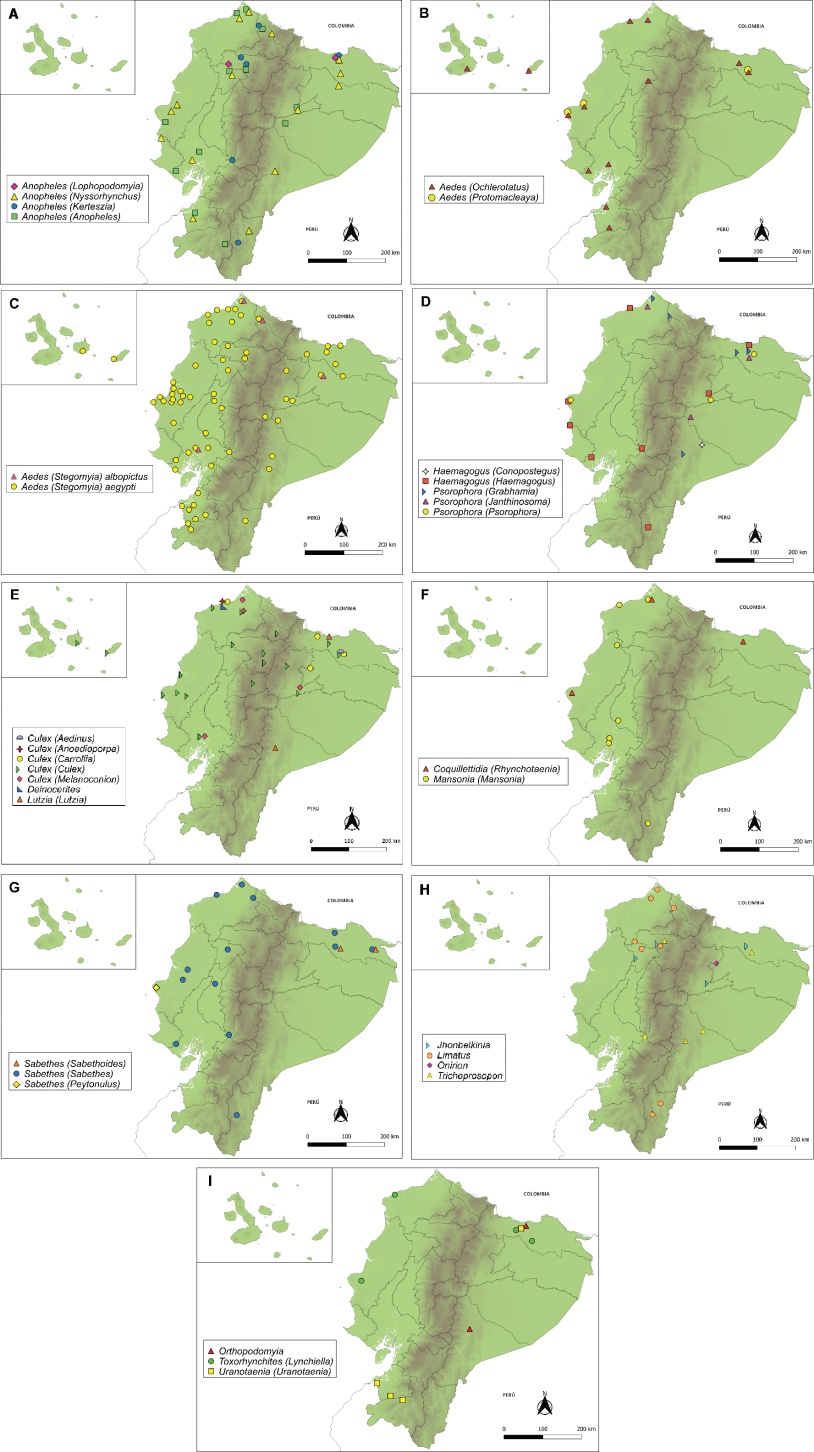
Geographic distribution of genera collected and preserved in the National Collection of Arthropod Vectors. **A**. *Anopheles* genus. **B–D**. Tribe Aedini. **E**. Tribe Culicini. **F**. Tribe Mansoniini. **G, H**. Tribe Sabethini. **I**. Tribe Orthopodomyiini, Toxorhynchitini, and Uranotaeniini. Each map includes an inset providing a separate view of the Galápagos Islands.

A total of 266 mosquito species, representing 22 genera of the family Culicidae, are listed alphabetically. The number of species per subfamily, tribe, and genus is summarized in Table 1. The geographic distribution of the species collected and preserved in the National Collection of Arthropod Vectors is presented in Fig. 1A–I.

**Table 1.** Summary of mosquito species richness by subfamily, tribe, and genus, including the total number of species recorded in Ecuador.

**Subfamily: Anophelinae (45 species**)		
**Tribe**	**Genus**	**Number of species**
N/A	* Anopheles *	42
N/A	* Chagasia *	3
**Subfamily: Culicinae (221 species)**		
Aedeomyiini	* Aedeomyia *	1
Aedini	* Aedes *	27
	* Haemagogus *	13
	* Psorophora *	14
Culicini	* Culex *	83
	* Deinocerites *	1
	* Galindomyia *	1
	* Lutzia *	2
Mansoniini	* Coquillettidia *	6
	* Mansonia *	5
Orthopodomyiini	* Orthopodomyia *	3
Sabethini	* Johnbelkinia *	2
	* Limatus *	5
	* Onirion *	1
	* Runchomyia *	1
	* Sabethes *	14
	* Trichoprosopon *	9
	* Wyeomyia *	20
Toxorhynchitini	* Toxorhynchites *	7
Uranotaeniini	* Uranotaenia *	6
**Total number of species**		**266**

Data for 60 species warrant further scrutiny, most originally described or recorded by Roberto Levi-Castillo. The absence of type specimens from his records requires further sampling and analyses to confirm their taxonomic identities and prevent misidentifications. The fate of all the mosquito specimens deposited by Levi-Castillo at the Centro Ecuatoriano de Investigaciones Entomológicas in Guayaquil during the 1950s remains unknown. Among these, Culex (Culex) guayasi and Culex (Culex) quitensis remain unrecognized, leaving their status unresolved (Bram 1967). Additionally, although *Lutzia* and *Onirion* are treated as genera in this checklist, their taxonomic placement remains under debate (Harbach 2007; Kitching et al. 2015).

**Figure 2.**
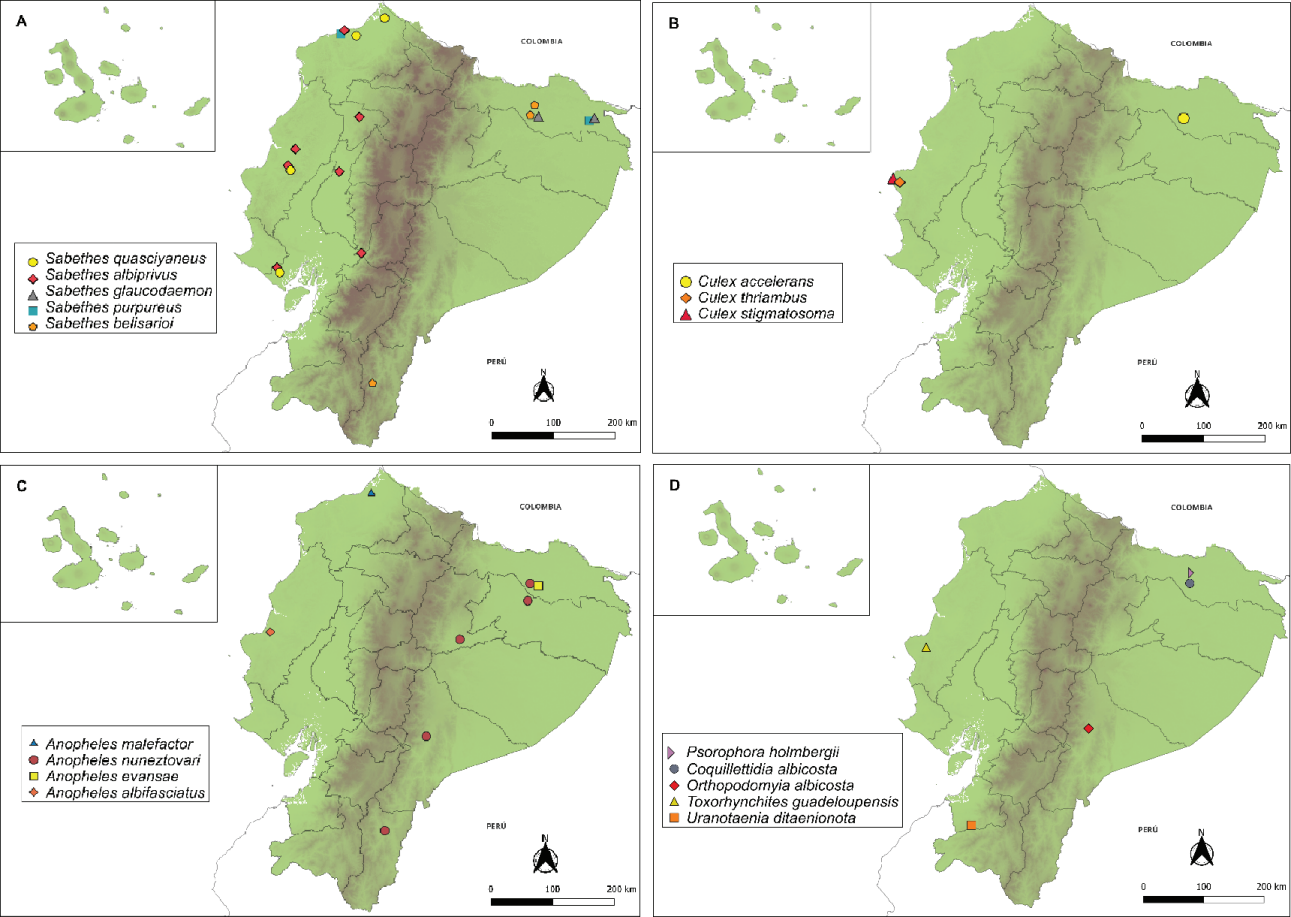
Geographic distribution of new mosquito records for Ecuador by species. **A**. *Sabethes* spp. **B**. *Culex* spp. **C**. *Anopheles* spp. **D**. *Psorophora*, *Coquillettidia*, *Orthopodomyia*, *Toxorhynchites* and *Uranotaenia*. Mapped occurrences correspond to data presented in Table 2. Each map includes an inset providing a separate view of the Galápagos Islands.

Detailed information on 17 mosquito species documented as new records is provided in Table 2. For each species, we provide the collection locality, date, habitat, collection method, and institutional code. These specimens were collected between 2010 and 2025 from multiple localities across the country. Their geographical distribution is illustrated in Fig. 2A–D. The geographic coordinates of the collection sites are provided in Suppl. material 1.

**Table 2.** Information on new mosquito records for Ecuador. Taxonomic classification, collection locations, collection dates, and collection methods are detailed.

**N°**	**Species**	**Locality**	**Date**	**Habitat**	**Collection methods**	**Institution code**
**1**	Anopheles (Anopheles) malefactor	Esmeraldas: Borbón	26 Jan. 2022	Secondary Forest	CDC Trap	ECU27944
**2**	Anopheles (Nyssorhynchus) evansae	Sucumbíos: Shushufindi	20 Jul. 2015	Periurban	Manual (immatures)	ECU8136, ECU8137
**3**	Anopheles (Nyssorhynchus) nuneztovari	Zamora Chinchipe: Yantzaza	23 Jul. 2015	Secondary Forest	Manual (immatures)	ECU8123, ECU8124, ECU8130
		Morona Santiago: Sucúa	18 Aug. 2015	Periurban	Manual (immatures)	ECU8538, ECU8539, ECU9900
		Napo: Puerto Misahuallí	18 May. 2015	Secondary Forest	Manual (immatures)	ECU7297 ECU7298
		Sucumbíos: Shushufindi	17 Jul. 2015	Secondary Forest	Manual (immatures)	ECU8134, ECU8135
		Orellana: El Edén	26 Apr. 2018	Secondary Forest	CDC Trap	ECU21452
**4**	Aedes (Ochlerotatus) albifasciatus	Manabí: Rocafuerte	24 Jul. 2014	Urban	Manual (immatures)	ECU10903
**5**	Psorophora (Psorophora) holmbergii	Sucumbíos: Pacayacu	22 Jul. 2014	Primary Forest	Human bait	ECU12276
**6**	Culex (Aedinus) accelerans	Sucumbíos: Shushufindi	26 May. 2015	Secondary Forest	Human bait	ECU3227
**7**	Culex (Culex) stigmatosoma	Manabí: Pacoche	12 Feb. 2016	Secondary Forest	Manual (immatures)	ECU12428, ECU12429, ECU12431, ECU12432
**8**	Culex (Culex) thriambus	Manabí: Pacoche	11 Feb.2016	Secondary Forest	CDC Trap	ECU10558
**9**	Coquillettidia (Rhynchotaenia) albicosta	Sucumbíos: Sushufindi	23 Jul. 2010	Periurban	Prokopack Aspirator	ECU10696
**10**	*Orthopodomyia albicosta*	Morona Santiago: Macas	16 Feb. 2014	Periurban	Manual (immatures)	ECU11574, ECU11586, ECU11590
**11**	Sabethes (Sabethes) albiprivus	Chimborazo: Cumandá	26 Aug. 2014	Periurban	Manual (immatures)	ECU6283
		Esmeraldas: Rioverde	20 May. 2015	Periurban	Manual (immatures)	ECU7244, ECU8059
		Guayas: Progreso	20 May. 2015	Periurban	Manual (immatures)	ECU7154
		Los Ríos: Quevedo	01 Jan. 2014	Periurban	Manual (immatures)	ECU6094
		Manabí: Chone	14 May. 2014	Urban	Manual (immatures)	ECU6121
		Manabí: Cantón Junín	07 Jul. 2014	Periurban	Manual (immatures)	ECU6441
		Santo Domingo de los Tsáchilas: Sueño de Bolívar	22 Sep. 2015	Periurban	Manual (immatures)	ECU10100
**12**	Sabethes (Sabethes) belisarioi	Zamora Chinchipe: Copalinga	14 Nov. 2009	Primary Forest	Prokopack Aspirator	ECU10718
		Sucumbíos: Shushufindi	30 Jun. 2014	Secondary Forest	Human bait	ECU16528
		Sucumbíos: Pacayacu	04 Agu. 2014	Secondary Forest	Manual	ECU13405
**13**	Sabethes (Sabethes) purpureus	Esmeraldas: Rioverde	31 Jul. 2015	Periurban	Manual (immatures)	ECU8528
		Sucumbíos: Cuyabeno	17 Apr. 2010	Primary Forest	Prokopack Aspirator	ECU10720
**14**	Sabethes (Sabethes) quasicyaneus	Esmeraldas: San Lorenzo	28 Oct. 2012	Periurban	Manual (immatures)	ECU3767
		Esmeraldas: Vía El Alto	31 Jul. 2015	Secondary Forest	Manual (immatures)	ECU8531
		Guayas: Progreso	22 Apr. 2015	Periurban	Manual (immatures)	ECU7158
		Manabí: Junín	07 Jul. 2014	Secondary Forest	Manual	ECU20172
**15**	Sabethes (Sabethoides) glaucodaemon	Sucumbíos: Cuyabeno	20 May. 2010	Secondary Forest	Prokopack Aspirator	ECU10716
		Sucumbíos: Shushufindi	21 Apr. 2015	Periurban	Prokopack Aspirator	ECU7093
**16**	Toxorhynchites (Lynchiella) guadeloupensis	Manabí: La Pila	24 Oct. 2014	Periurban	Manual (larvae)	ECU11017
**17**	Uranotaenia (Uranotaenia) ditaenionota	El Oro: Marcabelí	12 Apr. 2013	Periurban	Manual (larvae)	ECU4911

## An updated checklist of Diptera: Culicidae

Each entry provides the synonymic names, taxonomic author, year of publication, recorded localities, and relevant literature references. The geographical distribution of the species is categorized into four regions: East (E, Amazon basin), West (W, Pacific coastal lowlands), Andean (A, Andean region), and the Galápagos Islands (G). The institutional reference number (ECU) is provided for species deposited in the National Collection of Arthropod Vectors at INSPI-Quito. The notation NR indicates newly recorded species for Ecuador, while ** denotes endemic species and **†** denotes species whose type locality is Ecuador. Records based solely on GBIF entries should be treated cautiously, as additional verification is required to exclude potential misidentifications.

### Subfamily Anophelinae (45 species)

**1. Anopheles (Anopheles) apicimacula Dyar & Knab, 1906**.

**Distribution**. Sto. Domingo de los Tsáchilas: Valle Hermoso, Reserva La Perla (0°05'10.0"S, 79°16'21.0"W) (GBIF-Secretariat 2025). INSPI collection—Pichincha, near Mindo (0°06'44.8"S, 78°47'02.8"W) ECU7024; Esmeraldas: Borbón (1°05'47.4"N, 78°59'47.2"W) ECU27957, ECU27954, ECU27947. (W).

**2. Anopheles (Anopheles) calderoni Wilkerson, 1991**.

**Distribution**. Guayas: Yaguachi, “Hacienda Eulalia” (2°07'09.8"S, 79°41'22.9"W); Sto. Domingo de los Tsáchilas: near Tinalandia (0°16'18.0"S, 79°04'51.0"W), Valle Hermoso, Reserva La Perla (0°05'10.0"S, 79°16'21.0"W) (González et al. 2010). INSPI collection—Guayas: Eloy Alfaro (Durán) (2°09'51.9"S, 79°50'43.8"W) ECU1894; Esmeraldas: San Lorenzo (1°13'49.2"N, 78°46'00.7"W) ECU13903; Napo: Tena, Ahuano, Jatun Sacha (1°03'26.1"S, 77°36'23.0"W) ECU3473; El Oro: Arenillas ECU10372; Sto. Domingo de los Tsáchilas: Santo Domingo (0°13'20.7"S, 79°09'21.1"W) ECU19704; Esmeraldas: Borbón (1°04'55.0"N, 78°59'41.2"W) ECU27946. (E, W).

**3. Anopheles (Anopheles) eiseni Coquillett, 1902**.

**Synonym**. *An. niveopalpis* Ludlow, 1919.

**Distribution**. Morona Santiago and Zamora Chinchipe (formerly Santiago-Zamora): Cusuime, Huambi, Sucúa, Cutucú, Méndez, La Unión, Tindiuquinanda, El Tesoro (Ramírez-Aguilar 1953); Chimborazo near Huigra (2°19'2.4"S, 79°4'33.0"W); Cotopaxi near Tingo (0°54'24.6"S, 79°3'30.6"W); Pichincha: Mindo fish farm (0°4'9.0"S, 78°45'42.6"W) (Pinault and Hunter 2011). INSPI collection—Esmeraldas: Chuchubí (0°53'03.8"N, 78°30'56.0"W) ECU3773; Pastaza: Mariscal Sucre (1°21'35.9"S, 77°51'59.8"W) ECU11012. (A, E, W).

**4. Anopheles (Anopheles) fluminensis Root, 1927**.

**Synonym**. *An. holmbergi* Pont & Heredia, 1945.

**Distribution**. Orellana: Yasuní National Park, Tiputini Biodiversity Station, Harpía Trail, 800 m a.s.l. (00°38'02.7"S, 076°08'43.3"W) (Linton et al. 2013). INSPI collection—Napo: Tena, Ahuano, Jatun Sacha (1°03'26.1"S, 77°36'23.0"W) ECU3474. (E).

**5. Anopheles (Anopheles) forattinii Wilkerson & Sallum, 1999**.

**Distribution**. Orellana: Yasuní National Park, Tiputini Biodiversity Station, Harpía Trail, 1,500 m a.s.l. (00°37'32.3"S, 076°08'52.0" W) (Linton et al. 2013). (E).

**6. Anopheles (Anopheles) malefactor Dyar & Knab, 1907**.

**Distribution**. INSPI collection—Esmeraldas: Borbón (1°05'47.4"N, 78°59'47.2"W) ECU 27944. (NR, W).

**7. Anopheles (Anopheles) mattogrossensis Lutz & Neiva, 1911**.

**Synonym**. *An. amazonicus* Christophers, 1923.

**Distribution**. Ecuador (Gorham et al. 1973); Orellana: Yasuní National Park, Tiputini Biodiversity Station, lake ~ 4 hectares (00°37'32.3"S, 076°08'52"W) (Linton et al. 2013). (E).

**8. Anopheles (Anopheles) mediopunctatus (Lutz, 1903)**.

**Synonyms**. *An. costalimai* Courtinho, 1944; *An. limai* Fonseca & Silva Ramos, 1939; *Cyclolepidopteron rockefelleri* Peryassú, 1923; *Cycloleppteron mediopunctatus* Theobald, 1903.

**Distribution**. Guayas: Guayaquil and its surroundings (Campos 1925); Esmeraldas; Los Ríos; Manabí; Guayas (Levi-Castillo 1945b); Ecuador (Levi-Castillo 1958). (W).

**9. Anopheles (Anopheles) neomaculipalpus Curry, 1931**.

**Distribution**. INSPI collection—Pichincha, near Mindo (0°06'44.8"S, 78°47'02.8"W) ECU7023; Sto. Domingo de los Tsáchilas: Santo Domingo (0°15'06.7"S, 79°10'58.5"W) ECU8235. (W).

**10. Anopheles (Anopheles) peryassui Dyar & Knab, 1908**.

**Synonyms**. *An. celidopus* Dyar & Shannon, 1925; *An. alagoani* Peryassu, 1925; *Manguinhosia lutzi* Cruz, 1907.

**Distribution**. Ecuador (Levi-Castillo 1958).

**11. Anopheles (Anopheles) pseudopunctipennis Theobald, 1901**.

**Synonyms**. *An. pseudopunctipennis* spp. levicastilloi Levi-Castillo, 1944; *An. pseudopunctipennis* spp. *neghmei* Mann, 1950; *An. pseudopunctipennis* spp. *noei* Mann, 1950; *An. rivadeneirai*; *An. argentinus* Brèthes, 1912; *An. tucumanus* Lahille, 1912; An. var. bifoliata Osorno-Mesa & Munoz-Sarmiento, 1948; *An. willardi* Vargas, 1941.

**Distribution**. Guayas: Chongón, km 18–19 on Rt. 3, near road shrine (02°11'32.7"S, 80°02'24"W); Esmeraldas: La Propicia 1 (00°56'0.2"N, 079°39'28.6"W); Guayas: just S of Valdivia: San Pedro (01°56'54.4"S, 080°43'56.9"W) (GBIF-Secretariat 2025); from sea level to 2,500 m a.s.l. (Levi-Castillo 1945a); Loja; Pichincha; Imbabura; up to 1,930 m a.s.l. (Pinault and Hunter 2011); Imbabura: Cachaco (0°49'41.2"N, 78°24'03.6"W) (Navarro et al. 2015). INSPI collection—Manabí, Puerto Cayo (1°20'47.5"S, 80°39'15.4"W) ECU20423; Guayas: Progreso (2°28'00.7"S, 80°24'43.8"W) ECU7137; Imbabura: Cachaco (0°49'56.8"N, 78°24'00.1"W) ECU3774; Manabí: Puerto Cayo (1°20'47.5"S, 80°39'15.4"W) ECU20444; Loja: Malacatos (4°13'17.7"S, 79°15'38.9"W) ECU6115. (A, W).

**12. Anopheles (Anopheles) punctimacula Dyar & Knab, 1906**.

**Synonyms**. *An. venezuelae* Evans, 1922; *An. strigimacula* Dyar & Knab, 1906; *An. malefactor* Dyar & Knab, 1907; *An. apicimacula* Howard et al. (nec Dyar & Knab), 1917; *An. venezuelae* Evans, 1922.

**Distribution**. Coastal-side foothills of the Andes, 147–1,906 m a.s.l. (Pinault and Hunter 2011). INSPI collection—El Oro: El Retiro (3°26'02.3"S, 79°57'57.5"W) ECU19017; Sucumbíos: General Farfán (0°09'54.9"N, 76°40'39.0"W) ECU18675; Esmeraldas: Maldonado (1°04'03.0"N, 78°54'38.5"W) ECU27630. (A, E, W).

**13. Anopheles (Anopheles) shannoni Davis, 1931**.

**Distribution**. Ecuador (Levi-Castillo 1958); assumed to be in Amazonian Ecuador (Wilkerson et al. 1997). (E).

**14. Anopheles (Anopheles) vestitipennis Dyar & Knab, 1906**.

**Distribution**. El Oro; Orellana (Quinatoa Tutillo et al. 2025). (E, W).

**15. Anopheles (Kerteszia) bambusicolus Komp, 1937**.

**Distribution**. Napo; Pastaza; Santiago-Zamora (Gabaldon and Cova-Garcia 1952); Orellana: Tiputini River, 6 km from Tiputini Biodiversity Station (00°39'50"S, 076°12'56"W) (Linton et al. 2013). INSPI collection— Pichincha: Mindo (0°00'41.0"N, 78°48'33.2"W) ECU10392. (E, W).

**16. Anopheles (Kerteszia) boliviensis (Theobald, 1905)**.

**Synonym**. *Ker. boliviensis* Theobald, 1905.

**Distribution**. Napo, Orellana, and Pastaza (formerly Napo-Pastaza); Morona Santiago and Zamora Chinchipe (formerly Santiago-Zamora) (Levi-Castillo 1945b; Gabaldon and Cova-Garcia 1952). (E).

**17. Anopheles (Kerteszia) cruzii Dyar & Knab, 1908**.

**Distribution**. Ecuador (Lane 1953).

**18. Anopheles (Kerteszia) homunculus Komp, 1937**.

**Synonym**. *An. anoplus* Komp, 1937.

**Distribution**. Ecuador (Levi-Castillo 1949a).

**19. Anopheles (Kerteszia) lepidotus Zavortink, 1973**.

**Distribution**. Orellana (0°38'24.0"S, 76°08'24.0"W) (GBIF-Secretariat 2025); Orellana: Yasuní National Park, Tiputini Biodiversity Station (0°38'17.00"S, 76°8'42.00"W) (Harrison et al. 2012; Linton et al. 2013). (E).

**20. Anopheles (Kerteszia) neivai Howard, Dyar & Knab, 1913**.

**Synonym**. *An. hylephilus* Dyar & Knab, 1917.

**Distribution**. Guayas: Guayaquil, Posorja (Campos 1922, 1925); Bolívar: Balzapamba (01°46'00.9"S, 079°10'55.3"W); Guayas: Guayaquil (02°08'19.5"S, 079°55'00.6"W); Los Ríos: Montalvo (01°47'23.1"S, 079°17'14.8"W); Pichincha: Tandapi (00°24'56.3"S, 078°47'53.7"W) (Zavortink 1973); Esmeraldas; Manabí; Guayas: Guayaquil, El Salado (02°10'43.6"S, 79°54'15.2"W); Sto. Domingo de los Tsáchilas: near Tinalandia (00°17'35"S, 079°03'50"W), Valle Hermoso (00°05'10"S, 079°16'21"W); Orellana: Tiputini River, 6 km from Tiputini Biodiversity Station (00°39'50"S, 076°12'56"W) (GBIF-Secretariat 2025); Morona Santiago: Cusuime (Ramírez-Aguilar 1953); Imbabura: Lita (0°52'11.8"N, 78°27'05.6"W), Cachaco (0°49'41.2"N, 78°24'03.6"W); Esmeraldas: Alto Tambo (0°55'49.00"N, 78°32'37.00"W) (Navarro et al. 2015). INSPI collection—Sucumbíos: Nueva Loja (0°12'15.1"N, 76°39'27.2"W) ECU18669, Pichincha: 1.5 km from the Pachijal River (0°10'14.8"N, 78°55'51.0"W) ECU3222; Pichincha: Mindo (0°00'41.0"N, 78°48'33.2"W) ECU10403; Esmeraldas: Chuchubí (0°53'03.8"N, 78°30'56.0"W) ECU8589; Zamora Chinchipe: Parque Nacional Podocarpus (4°07'40.6"S, 78°59'44.2"W) ECU11367; Chimborazo: Cumandá (2°12'46.1"S, 79°07'54.9"W) ECU18643. (A, E, W).

**21. Anopheles (Kerteszia) pholidotus Zavortink, 1973**.

**Distribution**. Pichincha, Mindo, Sachatamia Lodge, at km 78 on the Quito-Calacali highway (GBIF-Secretariat 2025); Pastaza (Quinatoa Tutillo et al. 2025). (E, W)

**22. Anopheles (Lophopodomyia) gilesi (Peryassú, 1908)**.

**Synonym**. *Myzorhynchella gilesi* Peryassú, 1908.

**Distribution**. Pastaza (Quinatoa Tutillo et al. 2025). (E).

23. Anopheles (Lophopodomyia) gomezdelatorrei Levi-Castillo, 1955**†.

**Distribution**. Type locality: Carchi, Chiltazón, 2,880 m a.s.l. (Levi-Castillo 1955c, 1958; Stone et al. 1959; Gorham et al. 1973). (A).

**24. Anopheles (Lophopodomyia) squamifemur Antunes, 1937**.

**Distribution**. Orellana: Yasuní National Park, 3.5 km from Tiputini Biodiversity Station (00°38'18"S, 76°10'55"W) (Linton et al. 2013). INSPI collection—Sucumbíos: Nueva Loja (0°09'55.5"N, 76°40'34.3"W) ECU18602; Pichincha: Caoní River (0°00'48.4"N, 79°09'36.0"W) ECU14931. (E, W).

**25. Anopheles (Lophopodomyia) vargasi Gabaldón, Cova García & Lopez, 1941**.

**Distribution**. Morona Santiago, Sucúa, Parroquia Huambi (2°32'00.0"S, 78°10'00.1"W) (Ramírez-Aguilar 1953); Ecuador (Levi-Castillo 1958). (E).

**26. Anopheles (Nyssorhynchus) albimanus Wiedemann, 1820**.

**Synonyms**. *An. albipes* Theobald, 1901; *An. bisignatus* Hoffmann, 1938; *An. cubensis* Agramonte, 1900; *An. gorgasi* Dyar & Knab, 1907; *An. tarsimaculata* Goeldi, 1905; *An. trisignatus* Hoffmann, 1938.

**Distribution**. Esmeraldas: near Las Palmas Port (0°59'20.6"N, 79°38'54.8), near Atacames (0°52'34.4"N, 79°50'45.6"W), Isla La Burrera (00°57'20.5"N, 79°38'33.8"W), road to Muisne (00°46'37"N, 079°55'56"W); Guayas: Chongón, at km 24 on Rt. 3, near road to Chongón (02°13'10.5"S, 080°05'05.8"W); Guayaquil (02°12'05"S, 079°53'46"W); Milagro River (02°08"12"S, 079°41'07.9"W); Los Ríos: Pichilingue (01°06'11.9"S, 079°29'7.8"W); Pichincha: Reserva La Perla, Valle Hermoso (00°05'43.7"S, 079°18'06.3"W) (Heinemann and Belkin 1979); Imbabura, Mira River valley, 767—832 m a.s.l. (00°32'32.5"N, 078°02'34.6"W); Pichincha: near La Hesperia Biological Station, 1,366 m a.s.l. (00°21'00.1"S, 078°51'13.2"W); Azuay: near Girón, 1,541 m a.s.l. (03°09'35"S, 079°08'49.3"W) (Pinault and Hunter 2011); Esmeraldas: Borbón, Muisne (Kroeger et al. 1995); Imbabura: Cachaco (0°49'41.2"N, 78°24'03.6"W) (Navarro et al. 2015). INSPI collection—Imbabura, 90 m of San Lorenzo road (0°44'27.9"N, 78°13'58.4"W) ECU8587; Manabí: Rocafuerte (0°54'37.5"S, 80°26'08.6"W) ECU6647; Guayas: Guayaquil (2°14'23.1"S, 80°05'01.9"W) ECU6377; Napo: Puerto Misahuallí (1°01'41.8"S, 77°37'42.5"W) ECU7174; Esmeraldas: Borbón (1°05'33.2"N, 78°59'07.6"W) ECU28826 (A, E, W).

**27. Anopheles (Nyssorhynchus) albitarsis Lynch Arribálzaga, 1878**.

**Synonyms**. *An. allopha* Peryassu, 1939; *An. domesticus* Galvao & Damasceno, 1944; *An. imperfectus* Correa & Ramos, 1943; *An. limai* Galvao & Lane, 1937.

**Distribution**. Ecuador (AFPMB 1998).

**28. Anopheles (Nyssorhynchus) aquasalis Curry, 1932**.

**Synonyms**. *An. delta* Anduze, 1948; *An. deltaorinoquensis* Cova García, Pulido & Amarista, 1977; *An. emilianus* Komp, 1941; *An. guarauno* Anduze, 1948; *An. guarujaensis* Ramos, 1942.

**Distribution**. Guayas: Guayaquil Gulf (Forattini 1965). INSPI collection— Esmeraldas: San Lorenzo (1°14'58.8"N, 78°45'59.6"W) ECU18146; El Oro: Arenillas (3°32'54.2"S, 80°03'52.9"W) ECU10357; Manabí: El Guayabal (1°03'48.2"S, 80°34'06.4"W) ECU11658. (W).

**29. Anopheles (Nyssorhynchus) argyritarsis Robineau-Desvoidy, 1827**.

**Synonym**. *An. rooti* Brèthes, 1926.

**Distribution**. INSPI collection—Manabí: Ayampe (1°40'46.2"S, 80°48'13.4"W) ECU6953. (W).

**30. Anopheles (Nyssorhynchus) benarrochi Gabaldón, Cova García & López, 1941**.

**Distribution**. Morona Santiago: Taisha, Wachirpas (2°34'48.0"S, 76°48'00.0"W) (GBIF-Secretariat 2025). INSPI collection—Sucumbíos: Shushufindi (0°10'55.1"S, 76°38'07.5"W) ECU9797 (E).

**31. Anopheles (Nyssorhynchus) darlingi Root, 1926**.

**Synonym**. *An. paulistensis* Galvao, Lane & Correa, 1937.

**Distribution**. East of the Andes (Faran and Linthicum 1981); Ecuador (Levi-Castillo 1958; Arregui et al. 2015; GBIF-Secretariat 2025).

**32. Anopheles (Nyssorhynchus) evansae Brèthes, 1926**.

**Synonyms**. *An. noroestensis* Galvao & Lane, 1937; *An. ayrozai* Unti, 1940; *An. clarki* Komp, 1942; *An. metcalfi* Galvão & Lane, 1937; *Cel. evansae* Brèthes, 1926; *Cel. evansi* Brèthes, 1926.

**Distribution**. INSPI collection—Sucumbíos: Shushufindi (0°15'0.60"S, 76°36'48.08"W) ECU8136, ECU8137. (NR, E).

**33. Anopheles (Nyssorhynchus) konderi Galvão & Damasceno, 1942**.

**Distribution**. Orellana: Yasuní National Park, Tiputini Biodiversity Station, lake ~ 4 hectares (0°38'13.52"S, 76°9'0.33"W); Harpía Trail, 1,500 m a.s.l. (0°37'32.30"S, 76° 8'52.03"W); Tiputini River, blackwater area ~ 3.5 km from Tiputini Biodiversity Station (0°38'18.00"S, 76°10'55.00"W); Maquisapa Trail (0°37'34.91"S, 76°10'12.13"W) (Linton et al. 2013); Orellana: Coca, Cañón de los Monos (0°20'36.28"S, 77°0'25.23"W), Juan Montalvo (0°28'21.00"S, 76°59'29.04"W), Guamayacu, Tiputini (0°38'17.16"S, 76°8'42.01"W) (Ruiz-Lopez et al. 2013). (E).

**34. Anopheles (Nyssorhynchus) marajoara Galvão & Damasceno, 1942**.

**Distribution**. Sucumbíos (Quinatoa Tutillo et al. 2025). (E).

**35. Anopheles (Nyssorhynchus) nuneztovari Gabaldón, 1940**.

**Synonym**. *An. goeldii* Rozeboom & Gabaldón, 1941.

**Distribution**. INSPI collection—Zamora Chinchipe: Yantzaza (78°45'45.26" W 03°49'50.98"S) ECU8123, ECU8124, ECU8130; Morona Santiago: Sucúa (02°27'18.93"S, 78°09'32.05"W) ECU8538, ECU8539, ECU9900; Napo: Puerto Misahuallí (1°1'56.97"S, 77°40'14.37"W) ECU7297, ECU7298; Sucumbíos: Shushufindi (0°12'55.70"S, 76°38'37.70"W) ECU8134, ECU8135; Orellana: El Edén (0°28'06.6"S, 76°40'43.7"W) ECU21452 (NR, E).

**36. Anopheles (Nyssorhynchus) oswaldoi (Peryassú, 1922)**.

**Synonyms**. *An. aquacaelestis* Curry, 1932; *An. metcalfi* Galvao & Lane, 1937.

**Distribution**. Orellana: Yasuní National Park, near Tiputini Biodiversity Station (00°38'18"S, 076°10'55"W) (GBIF-Secretariat 2025); Pastaza: Mera, 1,233 m a.s.l. (01°27'27.5"S, 078°06'37.3"W); Tungurahua: Río Verde, 1,230 m a.s.l. (01°24'13.3"S, 078°18'03.7"W); Napo: Tena and Archidona (00°54'31.1"S, 077°48'29.9"W); Pastaza: Puyo (01°29'42.6"S, 077°59'19.7"W); Zamora Chinchipe: Zamora (Pinault and Hunter 2011). INSPI collection—Napo: Tena, Jatun Sacha (1°03'26.1"S, 77°36'23.0"W) ECU3475; Morona Santiago: Río Blanco (2°20'15.3"S, 78°09'50.4"W) ECU7495. (E).

**37. Anopheles (Nyssorhynchus) rangeli Gabaldón, Cova García & López, 1940**.

**Distribution**. Morona Santiago: Sucúa; Sucumbíos (formerly Napo): Limoncocha (00°24'01.4"S, 076°38'01.5"W); Napo: Tena; Pastaza: Puyo (Faran 1980); Morona Santiago: Sucúa (02°28'12"S, 078°10'07.7"W); Napo: 1.5 km S from Tena (01°01'14"S, 077°49'01.5"W); Pastaza: 4 km N from Puyo (01°28'01.4"S, 077°58'01.5"W) (GBIF-Secretariat 2025); Morona Santiago: Arapicos, Cusuime, Huambi, Sucúa, Cutucú, Cuyatza, Tindiuquinanda, Ambazha, Catachiyacu, Santa Marianita, Ceipa, Miriumi, La Unión, Chapi, Asunción, Huambinimi, Quimi, San José, Sungaime, Cachayacu, Imbiano, Ambaza, La Banda, Guayataza, Yuquiaza, Yurupaza, Ieminbis, Topo (Ramírez-Aguilar 1953); Lower-Napo region: Morona Santiago, Napo, Pastaza (Faran and Linthicum 1981); Napo: Coca (Fritz et al. 2004). INSPI collection—Sto. Domingo de los Tsáchilas: Santo Domingo (0°13'20.7"S, 79°09'21.1"W) ECU19705; Sucumbíos: Shushufindi (0°07'55.5"N, 76°40'12.1"W) ECU14943; Zamora Chinchipe: Yantzaza (3°49'47.9"S, 78°42'06.4"W) ECU10084. (E, W).

**38. Anopheles (Nyssorhynchus) strodei Root, 1926**.

**Distribution**. It is presumably found in eastern Ecuador (Faran 1980); Esmeraldas; Morona Santiago (Quinatoa Tutillo et al. 2025). (E, W).

**39. Anopheles (Nyssorhynchus) triannulatus (Neiva & Pinto, 1922)**.

**Synonyms**. *An. bachmanni* Petrocchi, 1925; *An. chagasi* Galvao, 1941; *An. cuyabensis* Neiva & Pinto, 1923.

**Distribution**. Orellana (formerly Napo): Limoncocha (00°24'01.4"S, 076°38'01.5"W); Napo: Tena (00°59'48"S, 077°48'49.3"W); Pastaza: Puyo (Faran 1980); Morona Santiago: Sucúa (02°27'27.2"S, 078°10'15.1"W); Pastaza: Puyo (01°28'01.4"S, 077°59'01.5"W) (GBIF-Secretariat 2025). INSPI collection—Morona Santiago: Sucúa (2°27'18.9"S, 78°09'32.1"W) ECU9857; Morona Santiago: Sucúa (2°27'18.9"S, 78°09'32.1"W) ECU3198. (E).

**40. Anopheles (Nyssorhynchus) trinkae Faran, 1979†**.

**Distribution**. Type locality: Pastaza: Puyo (Faran 1979); Napo: Tena (00°59'48"S, 077°48'49.3"W); Pastaza: Puyo (01°28'01.4"S, 077°59'01.5"W) (Faran 1980); Napo: Sardina Yaku (Fritz et al. 2004); Orellana (formerly Napo): Coca (00°27'49"S, 076°58'18.8"W); Sucumbíos: Lago Agrio, (00°05'1.7"N, 076°53'0.1"W) (Lounibos et al. 1998); Pastaza: Puyo (1°28'1.03"S, 77°59'0.18"W); Napo: Tena (1°10'1.45"S, 77°53'1.50"W) (GBIF-Secretariat 2025). INSPI collection—Zamora Chinchipe: Yantzaza (3°49'46.8"S, 78°45'43.7"W) ECU10089; Morona Santiago: Sucúa (2°27'03.5"S, 78°08'34.9"W) ECU9913; Morona Santiago: Río Blanco (2°20'15.3"S, 78°09'50.4"W) ECU7508; Napo: Puerto Misahualli (1°01'57.0"S, 77°40'14.4"W) ECU7299; Napo: Tena (1°01'40.8"S, 77°37'37.6"W) ECU7183. (E).

**41. Anopheles (Stethomyia) kompi Edwards, 1930**.

**Distribution**. Ecuador (Levi-Castillo 1949a, 1958; Gorham et al. 1973).

**42. Anopheles (Stethomyia) nimbus (Theobald, 1902)**.

**Distribution**. Orellana; Morona Santiago (Quinatoa Tutillo et al. 2025). (E).

**43. *Chagasia bathana* (Dyar, 1928)**.

**Distribution**. Sto. Domingo de los Tsáchilas: Santo Domingo (0°15'19.7"S, 79°10'48.7"W) (Harbach and Howard 2009); Orellana: Tiputini Biodiversity Station (00°38'15.5"S, 076°09'59.3"W) (Sallum et al. 2002). (E, W).

**44. *Chagasia bonneae* Root, 1927**.

**Distribution**. Orellana (formerly Napo): Coca, confluence of Coca and Napo rivers, 250 m a.s.l. (0°28'00.0"S, 76°58'00.0"W) (Heinemann and Belkin 1979); Orellana: Yasuní National Park, Tiputini River, blackwater area ~ 3.5 km from Tiputini Biodiversity Station (00°38'18"S, 76°10'55"W) (Linton et al. 2013); Orellana (formerly Napo) Coca; Napo: Tena; Pastaza: Santa Ana (Harbach and Howard 2009). (E, W).

**45. *Chagasia fajardi* (Lutz, 1904)**.

**Synonyms**. *Ch. maculata* Peryassú, 1921; *Ch. neivae* Cruz, 1906; *Ch. stigmopteryx* Martini, 1932; *Pyretophorus fajardi* Lutz, 1904.

**Distribution**. Orellana: Yasuní National Park, Tiputini Biodiversity Station, Harpía Trail, 750 m a.s.l. (00°37'50.2"S, 076°08'43.4"W) (Linton et al. 2013). (E).

### Subfamily Culicinae. Tribe Aedeomyiini (1 species)

**46. Aedeomyia (Aedomyia) squamipennis (Lynch Arribálzaga, 1878)**.

**Synonym**. *Ae. squamipennis* Lynch Arribálzaga, 1878.

**Distribution**. Los Ríos: Babahoyo (Calisher et al. 1981). (W).

### Subfamily Culicinae. Tribe Aedini (54 species)

**47. Aedes (Aedimorphus) vexans (Meigen, 1830)**.

**Synonyms**. *Cx. parvus* Macquart, 1834; *Cx. articulatus* Rondani, 1872; *Cx. malariae* Grassi, 1898; *Cx.* sylvestris Theobaldo, 1901; *Ae. nocturnus* Theobald, 1903; *Cx. montcalmi* Blanchard, 1905; *Cda. minuta* Theoblad, 1907; *Cda. nipponii* Theobald, 1907; *Cdа. eruthrosops* Theobald, 1910; *Cx. sudanensis* Theobald, 1911; *Ae. euochrus* Howard, Dyar & Knab, 1917.

**Distribution**. Galápagos: ‘‘Archipiélago de Galápagos’’ (Campos 1922). (G).

**48. Aedes (Howardina) albonotatus (Coquillett, 1906)**.

**Synonym**. *Gy. albonotata* Coquillett, 1905.

**Distribution**. Pichincha; Imbabura; Bolívar; Chimborazo (Levi-Castillo 1952b, 1953d); Ecuador (Levi-Castillo 1958). (A).

49. Aedes (Howardina) brevivittatus Berlin, 1969**†.

**Distribution**. Type locality: Azuay: Zorrocucho, E of Girón, Cerro Tinajillas (03°11'12"S, 079°02'07.8"W) (Heinemann and Belkin 1979); Sto. Domingo de los Tsáchilas, 50 km SE from Tandapi (00°23'11.9"S, 078°46'07.7"W) (Berlin 1969). INSPI collection—Pichincha: Nanegalito, ECU 3258. (A, W).

50. Aedes (Howardina) ecuadoriensis Berlin, 1970**†.

**Distribution**. Type locality: Azuay: Oña, ~ 10 km N of León River at km 99 on Cuenca-Loja highway (3°32'00.0"S, 79°10'00.0"W) (Heinemann and Belkin 1979); Imbabura: Ibarra, Taguando River (0°21'00.0"N, 78°07'00.0"W) (Berlin 1969; Heinemann and Belkin 1979). (A).

**51. Aedes (Howardina) eleanorae Berlin, 1969**.

**Distribution**. Napo: Colonso Chalupas Biological Reserve, 1,230 m a.s.l. (0°56'25.2"S, 77°54'00.0"W) (Navarro et al. 2016). (E).

**52. Aedes (Howardina) fulvithorax (Lutz, 1904)**.

**Synonyms**. *Ae. tachirensis* Anduze, 1947; *Haemagogus fulvithorax* Lutz, 1904; *Taeniorhynchus palliatus* Coquillett, 1906.

**Distribution**. Pichincha: Quito D.M.; Pululahua Geobotanical Reserve; Pululahua Volcano, 2,101 m a.s.l. (0°03'28.2"N, 78°30'29.1"W) (Navarro et al. 2016). (A).

**53. Aedes (Howardina) pseudodominicii Komp, 1936**.

**Synonym**. *Ae. delpontei* Martinez & Prosen, 1955.

**Distribution**. Napo, Orellana, and Pastaza (formerly Napo-Pastaza); Pichincha: Tandapi (Levi-Castillo 1953c). (E).

**54. Aedes (Howardina) sexlineatus (Theobald, 1901)**.

**Synonyms**. *Stegomyia dominicii* Rangel & Romero Sierra, 1907; *St. sexlineatus* Theobald, 1901.

**Distribution**. Napo: Colonso-Chalupas Biological Reserve, 1,230 m a.s.l. (0°56'25.2"S, 77°54'00.0"W) (Navarro et al. 2016). INSPI collection— Pichincha: Nanegalito ECU3260; Pichincha: Quito ECU16510; Manabí: San Lorenzo ECU10666; Orellana: Loreto, Bigal River ECU8594; Loja: Alamor ECU19111. (A, E, W).

**55. Aedes (Howardina) quadrivittatus (Coquillett, 1902)**.

**Synonym**. *Cx. quadrivittatus* Coquillett, 1902.

**Distribution**. Azuay; Cañar; Pichincha; Imbabura (Levi-Castillo 1953c); Ecuador (Levi-Castillo 1958; AFPMB 1998). (A).

**56. Aedes (Ochlerotatus) albifasciatus (Macquart, 1838)**.

**Synonyms**. *Ae. colonarius* Dyar, 1924; *Ae. philippii* Dyar, 1924; *Cx. albifasciatus* Macquart, 1838; *Cx. annuliferus* Blanchard, 1852; *Cx. flavipes* Macquart, 1838; *Ochlerotatus albifasciatus* Macquart, 1838.

**Distribution**. Manabí: Rocafuerte, El Ceibal (0°55'25.07"S, 80°28'9.14"W) ECU10903. (NR, W).

**57. Aedes (Ochlerotatus) angustivittatus Dyar & Knab, 1907**.

**Synonyms**. *Aedes argentescens* Dyar & Knab, 1908; *Aedes cuneatus* Dyar & Knab, 1908; *Aedes traversus* Dyar, 1925.

**Distribution**. Guayas ~ 30 km SW of Quevedo, 3 km S of El Empalme, 100 m (01°06'12"S, 079°41'07.9"W); Los Ríos: Quevedo, Valencia (00°57'07.7"S, 079°21'05.9"W), Guayas: Samborondón, La Bocana estuary, road upstream from town (01°57'12"S, 079°44'7.9"W); outskirts of Los Ceibos (02°09'54.8"S, 79°55'55.6"W) (Arnell 1976; Heinemann and Belkin 1979). INSPI collection—Sucumbíos: Shushufindi (0°07'26.5"S, 76°39'02.6"W) ECU9827; Guayas: Guayaquil (2°24'30.5"S, 80°22'15.9"W) ECU7400; Esmeraldas: Borbón (1°05'05.6"N, 78°59'52.1"W) ECU 25390. (E, W).

**58. Aedes (Ochlerotatus) crinifer (Theobald, 1903)**.

**Synonyms**. *Cx. crinifer* Theobald, 1903; *Cx. lynchii* Brèthes, 1910.

**Distribution**. Ecuador (Stone et al. 1959; Arnell 1973; AFPMB 1998; Bánki et al. 2025).

**59. Aedes (Ochlerotatus) fluviatilis (Lutz, 1904)**

**Synonyms**. *Ae. draconarius* Dyar, 1922; *Ae. lithoecetor* Dyar & Knab, 1907; *Cx. fluviatilis* Lutz, 1904; *Danielsia mediomaculata* Theobald, 1907; *Dn. tripunctata* Theobald, 1907.

**Distribution**. Zamora Chinchipe: Zamora, ~ 10 km NE, tributary stream of Zamora River (04°04'12"S, 078°58'07.8"W) (Heinemann and Belkin 1979); Pastaza, 31 km W of Puyo (01°28'01.4"S, 078°16'01.5"W) (GBIF-Secretariat 2025). INSPI collection—El Oro: Piñas (3°43'39.6"S, 79°53'12.4"W) ECU11366; Sto. Domingo de los Tsáchilas: Santo Domingo (0°19'05.6"S, 78°59'02.0"W) ECU 11364. (E, W).

**60. Aedes (Ochlerotatus) fulvus (Wiedemann, 1828)**.

**Synonyms**. *Cx. flavicosta* Walker, 1856; *Cx. fulvus* Wiedemann, 1828.

**Distribution**. Guayaquil and its surroundings (Campos 1922, 1925); Orellana (formerly Napo), ~ 50 km E of El Coca, ‘‘Isla Pompeya’’ (00°26'12"S, 076°37'07.5"W); Orellana (formerly Napo): Coca, confluence of Coca and Napo rivers, 250 m a.s.l. (0°28'00.0"S, 76°58'00.0"W) (Heinemann and Belkin 1979). INSPI collection—Sucumbíos: Nueva Loja (0°05'20.0"N, 76°52'09.0"W) ECU3192; Shushufindi (0°10'14.9"S, 76°38'26.5"W) ECU7142. (E, W).

**61. Aedes (Ochlerotatus) hastatus Dyar, 1922**.

**Distribution**. Sucumbíos (formerly Napo): Cuyabeno, 50 km W of Tarapoa, Topography Camp, Aguas Negras (Heinemann and Belkin 1979). (E).

**62. Aedes (Ochlerotatus) hortator Ficalbi, 1889**.

**Distribution**. Orellana (formerly Napo): Coca, confluence of Coca and Napo rivers, 250 m a.s.l. (0°28'00.0"S, 76°58'00.0"W) (Heinemann and Belkin 1979). INSPI collection—Esmeraldas: Rioverde (1°04'05.3"N, 79°24'17.2"W) ECU7506. (E, W).

**63. Aedes (Ochlerotatus) meprai Martinez & Prosen, 1953**.

**Distribution**. Loja, ~ 20 km S of Loja, (04°18'01.4"S, 079°18'01.5"W) (Arnell 1976; Heinemann and Belkin 1979). (A).

**64. Aedes (Ochlerotatus) milleri Dyar, 1922**.

**Synonyms**. *Ae. araozi* Shannon & Ponte, 1927; *Ae. oroecetor* Martini, 1931.

**Distribution**. INSPI collection—Pichincha: Sangolqui (00°21'32.43"S, 78°25’ 29.33"W), ECU10041, ECU16263. (A).

**65. Aedes (Ochlerotatus) nubilus Theobald, 1903**.

**Synonyms**. *Ae. polyagrus* Dyar, 1949; *Cx. nubilus* Theobald, 1903.

**Distribution**. Orellana (formerly Napo): Coca, confluence of Coca and Napo rivers, 250 m a.s.l. (0°28'00.0"S, 76°58'00.0"W) (Heinemann and Belkin 1979). (E).

**66. Aedes (Ochlerotatus) scapularis (Rondani, 1848)**.

**Synonyms**. *Ae. camposanus* Dyar, 1918; *Ae. hemisurus* Dyar & Knab, 1906; *Cx. scapularis* Rondani, 1848; *Ochlerotatus confirmatus* Lynch Arribalzaga, 1891.

**Distribution**. Los Ríos: Babahoyo (01°48'10.1"S, 079°31'51.6"W); Manabí: Manta (00°57'11.8"S, 080°43'57.3"W) (Arnell 1976); Esmeraldas: Island near Las Palmas Port (00°56'55"N, 79°38'33"W); Guayas: Guayaquil, El Salado estuary, N of Catholic University of Santiago de Guayaquil (02°10'43.6"S, 079°54'15.2"W); Guayas: Guayaquil, just S of Pascuales, at km 9.5 on Rt. 2 (02°05'01.7"S, 079°56'28.2"W); Guayas: Guayaquil, ~ 5 km S of Escuela Guayas – Salado (02°16'25.5"S, 079°53'13.5"W); Guayas: Samborondón, La Bocana estuary, road upstream from town (01°57'12"S, 079°44'7.9"W); Guayas: Guayaquil, 5 km N of Mapasingue; Guayas: Guayaquil, swamp N of airport (02°08'49.5"S, 079°53'13.8"W) (Heinemann and Belkin 1979). INSPI collection—Zamora Chinchipe: Zamora, Copalinga; Sucumbíos: Shushufindi (0°10'55.4"S, 76°40'22.0"W) ECU19618; Guayas: Guayaquil (2°15'34.1"S, 79°54'18.6"W) ECU18872; El Oro: Machala (3°14'56.7"S, 79°57'41.8"W) ECU17814; Esmeraldas: Borbón (1°05'24.4"N, 78°59'32.4"W) ECU28266. (E, W).

**67. Aedes (Ochlerotatus) serratus (Theobald, 1901)**.

**Synonyms**. *Ae. meridionalis* Dyar & Knab, 1906; *Cx. mathisi* Neveu-Lemaire, 1902; *Cx. serratus* Theobald, 1901.

**Distribution**. Orellana: Yasuní National Park, Tiputini Biodiversity Station, Guacamayo Trail, 2,300 m a.s.l (00°38'02.4"S, 076°09'49.9"W); Orellana (formerly Napo): Coca, confluence of Coca and Napo rivers, 250 m a.s.l. (0°28'00.0"S, 76°58'00.0"W); Orellana (formerly Napo), ~ 50 km E of El Coca, ‘‘Isla Pompeya’’ (00°26'12"S, 076°37'07.5"W) (Heinemann and Belkin 1979; GBIF-Secretariat 2025); Orellana: Yasuní National Park, Tiputini Biodiversity Station, Guacamayo Trail, 2,300 m a.s.l. (0°38'2.42"S, 76°9'49.87"W) (Linton et al. 2013). INSPI collection—Sucumbíos: Shushufindi (0°10'55.4"S, 76°37'32.0"W) ECU19595; Manabí: Manta (1°07'12.6"S, 80°50'33.1"W) ECU10621; Esmeraldas: Borbón (1°05'24.4"N, 78°59'32.4"W) ECU27768. (E, W).

**68. Aedes (Ochlerotatus) taeniorhynchus (Wiedemann, 1821)**.

**Synonyms**. *Ae. epinolus* Dyar & Knab, 1914; *Ae. niger* Giles, 1904; *Ae. pix* Martini, 1935; *Cx. damnosus* Say, 1823; *Cx. portoricensis* Ludlow, 1905; *Cx. taeniorhynchus* Wiedemann, 1821.

**Distribution**. Guayas: Guayaquil; Durán; Posorja (Campos 1922, 1925); Guayas: Guayaquil, ~ 5 km on road to Esclusa Guayas-Salado, 2.3 km E of road to Puerto Nuevo; 10 km W of Guayaquil on Rt. 3; NW outskirts of Los Ceibos; Estero Salado just NW of Catholic University of Santiago de Guayaquil (Heinemann and Belkin 1979; GBIF-Secretariat 2025); Guayas: Guayaquil, three specimens caught in a plane flying from Guayaquil to Baltra, Galápagos (Bataille et al. 2009); Guayas: Playas, 0.5 km N of Playas (2°37'36.66"S, 80°23'25.84"W), 7 km SE of Playas on road to Posorja (2°40'52.00"S, 80°21'16.20"W) (GBIF-Secretariat 2025); Galápagos: Santa Cruz, Baltra, San Cristobal, Fernandina, Floreana, Isabela, Santiago, Española, Santa Fe (Stone et al. 1959; Whiteman et al. 2005; Bataille et al. 2009, 2012; Siers et al. 2010; Eastwood et al. 2013). INSPI collection—Guayas: Guayaquil (2°15'33.5"S, 79°54'18.6"W) ECU18888; El Oro: Machala (3°14'49.7"S, 79°57'44.0"W) ECU17224; Galápagos: San Cristobal (0°54'35.6"S, 89°35'36.8"W) ECU12903; Isabela (0°51'22.0"S, 91°01'32.1"W) ECU12580; Esmeraldas: Rioverde (1°03'38.6"N, 79°22'20.9"W) ECU8411; Esmeraldas: Borbón (1°05'24.4"N, 78°59'32.4"W) ECU28265. (W, G).

**69. Aedes (Protomacleaya) argyrothorax Bonne-Wepster & Bonne, 1920**.

**Distribution**. Orellana: Yasuní National Park, Tiputini Biodiversity Station (00°38'13.5"S, 076°09'0.3"W) (Linton et al. 2013; GBIF-Secretariat 2025). INSPI collection—Sucumbíos: Shushufindi (0°10'45.8"S, 76°39'03.6"W) ECU11005. Manabí: Rocafuerte (0°55'25.1"S, 80°28'09.1"W) ECU1097. (E).

70. Aedes (Protomacleaya) metoecopus Dyar, 1925**†.

**Synonym**. *Ae. terrens metoecopus* Levi-Castillo, 1953.

**Distribution**. Type locality: Guayas: Guayaquil, just S of Pascuales, at km 9.5 on Rt. 2 (02°05'01.7"S, 079°56'28.2"W); Guayas: Chongón, W of Guayaquil; Los Ríos: Quevedo, 1 km E from Valencia (00°56'46.9"S, 079°20'10.5"W) (Stone and Knight 1956; Belkin 1968); Los Ríos, ~ 30 km SW of Quevedo, 3 km S of El Empalme, 100 m a.s.l. (01°06'12"S, 079°41'07.9"W); Los Ríos: Montalvo, 30 km E of Babahoyo, “Hacienda Mora”, across river at end of main path of Montalvo (01°49'12"S, 079°31'07.8"W) (Schick 1970; GBIF-Secretariat 2025). (W).

**71. Aedes (Protomacleaya) terrens gr . (Walker, 1856)**.

**Synonyms**. *Ae. apollo* Schick, 1970; *Cx. terrens* Walker, 1856; *Gualteria oswaldi* Lutz, 1904.

**Distribution**. Guayas: Bucay; Guayas: Atahualpa, ~ 15 km SE of Chanduy (Heinemann and Belkin 1979); Carchi: El Baboso (0°53'47.05"N, 78°26'34.98"W) (Navarro et al. 2015). INSPI collection—Manabí: Río Seco (1°07'39.1"S, 80°50'35.5"W) ECU10551. (W).

**72. Aedes (Stegomyia) aegypti (Linnaeus, 1762)**.

**Synonyms**. *Cx. aegypti* Linnaeus, 1762; *Cx. albopalposus* Becker, 1908; *Cx. anguste-alatus* Becker, 1908; *Cx. annulitarsis* Macquart, 1844; *Cx. argenteus* Poiret, 1787; *Cx. augens* Wiedemann, 1828; *Cx. bancrofti* Skuse, 1889; *Cx. calopus* Meigen, 1818; *Cx. elegans* Ficalbi, 1889; *Cx. exagitans* Walker, 1856; *Cx. excitans* Walker, 1848; *Cx. fasciatus* Fabricius, 1805; *Cx. frater* Robineau-Desvoidy, 1827; *Cx. inexorabilis* Walker, 1848; *Cx. insatiabilis* Bigot, 1859; *Cx. kououpi* Brulle, 1833; *Cx. mosquito* Robineau-Desvoidy, 1827; *Cx. rossii* Giles, 1889; *Cx. taeniatus* Wiedemann, 1828; *Cx. toxorhynchus* Macquart, 1838; *Cx. viridifrons* Walker, 1848; *Duttonia alboannulis* Ludlow, 1911; *Mimeteomyia pulcherrima* Taylor, 1919; *St. atritarsis* Edwards, 1920; *Stegomyia canariensis* Pittaluga, 1905; *St. luciensis* Theobald, 1901; *St. nigeria* Theobald, 1901; *St. queenslandensis* Theobald, 1901.

**Distribution**. INSPI collection—widely spread all over the country, up to 1650 m a.s.l., ECU20713, ECU20767. (A, E, W, G).

**73. Aedes (Stegomyia) albopictus (Skuse, 1894)**.

**Synonyms**. *St. nigritia* Ludlow, 1910; *St. quasinigritia* Ludlow, 1911; *St. samarensis* Ludlow, 1903.

**Distribution**. INSPI collection—Guayas: Guayaquil (2°09'27.8"S, 79°54'20.0"W) ECU20101 (Ponce et al. 2018); Esmeraldas: Alto Tambo (0°53'30.5"N, 78°30'07.9"W) ECU27432; Imbabura: Cachaco (0°50'01.7"N, 78°24'06.1"W) ECU21370; Orellana: El Coca (0°27'38.5"S, 76°59'33.8"W) ECU27313. (E, W).

**74. Haemagogus (Conopostegus) leucotaeniatus (Komp, 1938)**.

**Synonym**. *Ae. leucotaeniatus* Komp, 1938.

**Distribution**. INSPI collection—Morona Santiago: Macuma, Wisui Biological Station (2°06'39.3"S, 77°44'21.3"W) ECU3520 (E).

**75. Haemagogus (Conopostegus) leucocelaenus Dyar & Shannon, 1924**.

**Synonyms**. *Ae. leucocelaenus* Dyar & Shannon, 1924; *Hg. leucomelas* Lutz, 1904.

**Distribution**. Guayas: Guayaquil (GBIF-Secretariat 2025); Ecuador (Navarro et al. 2013a) (E, W).

76. Haemagogus (Haemagogus) acutisentis Arnell, 1973**†.

**Distribution**. Type locality: Los Ríos: Quevedo, 1 km E from Valencia, banana and cocoa grove (00°56'46.9"S, 079°20'10.5"W); Los Ríos: Quevedo, 4 km W of Valencia; Los Ríos: Montalvo, 30 km E of Babahoyo, ‘‘Hacienda Mora”, across river at end of main path of Montalvo (01°49'12"S, 079°31'07.8"W); Guayas: Guayaquil, at km 14 on Rt. 3, ~ 1 km beyond checkpoint (02°11'01"S, 080°00'14.5"W); Guayas: Guayaquil, just S of Pascuales, at km 9.5 on Rt. 2 (02°05'01.7"S, 079°56'28.2"W) (Arnell 1973; Heinemann and Belkin 1979). (W).

**77. Haemagogus (Haemagogus) albomaculatus Theobald, 1903**.

**Distribution**. Guayas: El Salado (Campos 1922). INSPI collection—Guayas: Progreso (2°25'03.8"S, 80°18'41.7"W) ECU16505. (W).

**78. Haemagogus (Haemagogus) anastasionis Dyar, 1921**.

**Synonym**. *Hg. dominguezi* Duret, 1971.

**Distribution**. Zamora Chinchipe: Copalinga (04°05'26"S, 078°57'28.8"W). INSPI collection—Esmeraldas: Río Verde (1°02'54.0"N, 79°25'00.8"W) ECU19650. Sucumbíos: Nueva Loja (0°10'56.2"N, 76°38'51.1"W) ECU18575. Manabí: Montecristi (1°07'25.0"S, 80°50'43.9"W) ECU11423. Zamora Chinchipe: Copalinga (4°02'27.5"S, 78°59'16.4"W) ECU6101. (E, W).

**79. Haemagogus (Haemagogus) boshelli Osorno-Mesa, 1944**.

**Synonym**. *Hg. garciai* Levi-Castillo, 1955.

**Distribution**. Esmeraldas: Isla de Changuaral (01°22'52.3"N, 078°52'02.1"W) (Heinemann and Belkin 1979). (W).

**80. Haemagogus (Haemagogus) chalcospilans Dyar, 1921**.

**Note**. Described as Haemagogus (Osomomysa) garciai (Levi-Castillo, 1955).

**Distribution**. Guayas: Guayaquil, at km 10 on Rt. 3, patch of bamboo at the back of a farm (02°11'32.6"S, 079°58'05"W); Cañar: Cochancay, at km 86 on Rt. 8 from Guayaquil (02°28'08.4"S, 079°17'50.6"W); Esmeraldas: Isla de Changuaral, Bahía de Ancón de Sardinas (GBIF-Secretariat 2025). (W).

**81. Haemagogus (Haemagogus) equinus (Theobald, 1903)**.

**Synonyms**. *Ae. affirmatus* Dyar & Knab, 1906; *Ae. philosophicus* Dyar & Knab, 1906.

**Distribution**. Guayas: El Salado (Campos 1922). INSPI collection—Manabí: Ayampe (1°40'46.2"S, 80°48'13.4"W) ECU13549; Chimborazo: Cumandá (2°12'39.8"S, 79°08'00.3"W) ECU20080. (W).

**82. Haemagogus (Haemagogus) janthinomys Dyar, 1921**.

**Synonyms**. *Hg. falco* Kumm & Osorno-Mesa, 1946; *Hg. obscurescens* Martini, 1931; *Hg. petrocchiae* Martinez & Carcavallo, 1961.

**Distribution**. Orellana (formerly Napo): Coca, confluence of Coca and Napo rivers, 250 m a.s.l. (0°28'00.0"S, 76°58'00.0"W); Orellana: Yasuní National Park, near Lago Tiputini Biodiversity Station (00°38'08.2"S, 076°09'53.5"W) (Linton et al. 2013). INSPI collection—Napo: Tena (1°01'21.4"S, 77°34'31.2"W) ECU10706. (E).

**83. Haemagogus (Haemagogus) lucifer (Howard, Dyar & Knab, 1913)**.

**Synonym**. *Stegoconops lucifer* Howard, Dyar & Knab, 1912.

**Distribution**. Napo: Ila (formerly Napo-Pastaza) (Arnell 1973). (E).

84. Haemagogus (Haemagogus) panarchys Dyar, 1921**†.

**Distribution**. Type locality: Guayas: Guayaquil, El Salado (02°10'43.6"S, 79°54'15.2"W) (Stone et al. 1959; Belkin 1968); Guayas: Guayaquil, at km 14 on Rt. 3, ~ 1 km beyond checkpoint (02°11'01"S, 080°00'14.5"W); Guayas: Guayaquil, just S of Pascuales, at km 9.5 on Rt. 2 (02°05'01.7"S, 079°56'28.2"W); Los Ríos: Quevedo, 4 km W of Valencia (00°57'56.7"S, 079°25'14.5"W); Santa Elena (formerly Guayas): Chanduy, ~ 15 km SE of Atahualpa (Arnell 1973; Heinemann and Belkin 1979). (W).

85. Haemagogus (Haemagogus) soperi Leví-Castillo, 1955**†.

**Distribution**. Type locality: Los Ríos: Juan Montalvo (Levi-Castillo 1955a; Stone et al. 1959; Forattini 1965); Cañar: Cochancay, at km 86 on Rt. 8 from Guayaquil (02°28'08.4"S, 079°17'50.6"W); Los Ríos: Montalvo, 30 km E of Babahoyo, “Hacienda ‘‘Mora’’, across river at end of main path of Montalvo (01°49'12"S, 079°31'07.8"W); Los Ríos, ~ 30 km SW of Quevedo, 3 km S of El Empalme, 100 m a.s.l. (01°06'12"S, 079°41'07.9"W); Los Ríos: Quevedo, 1 km E from Valencia. (00°56'46.9"S, 079°20'10.5" W) (Arnell 1973; Heinemann and Belkin 1979; GBIF-Secretariat 2025). (W).

**86. Haemagogus (Haemagogus) spegazzinii Brèthes, 1912**.

**Synonyms**. *Hg. lindneri* Martini, 1931; *Hg. uriartei* Shannon & Ponte, 1927.

**Distribution**. Ecuador (Arnell 1973).

**87. Psorophora (Grabhamia) cingulata Fabricius, 1805**.

**Synonyms**. *Ae. garciai* Levi-Castillo, 1953; *Cx. apicalis* Theobald, 1903; *Cx. cingulatus* Fabricius, 1805; *Janthinosoma indoctum* Dyar & Knab, 1906; *Ps. neoapicalis* Theobald, 1910.

**Distribution**. Orellana: Yasuní National Park, Tiputini Biodiversity Station, Tower (0°39'52.99"S, 76°12'54.00"W); Orellana (formerly Napo): Coca, confluence of Coca and Napo rivers, 250 m a.s.l. (0°28'00.0"S, 76°58'00.0"W); Orellana (formerly Napo), ~ 50 km E of El Coca, ‘‘Isla Pompeya” (00°26'12"S, 076°37'07.5"W); Orellana (formerly Napo): Yasuní National Park, Tiputini Biodiversity Station, Tower (00°38'17"S, 076°08'42"W) (Linton et al. 2013). INSPI collection— Sucumbíos: Nueva Loja (0°01'57.6"S, 76°35'45.3"W) ECU20510. Imbabura: Lita, Chuchubí (0°52'07.6"N, 78°27'04.3"W) ECU10698. (E, W).

**88. Psorophora (Grabhamia) confinnis (Lynch Arribalzaga, 1891)**.

**Synonyms**. *Cx. scutipunctatus* Lutz & Neiva, 1911; *Ps. chiquitana* Pinto, 1932; *Ps. funiculus* Dyar, 1920; *Taeniorhynchus confinnis* Lynch Arribalzaga, 1891; *Taeniorhynchus trigonophorus* Lutz, 1928.

**Distribution**. Imbabura: Lita (0°52'11.83"N, 78°27'05.56"W); Cachaco (0°49'41.2"N, 78°24'03.6"W) (Navarro et al. 2015). INSPI collection— Esmeraldas: Alto Tambo (1°17'18.0"N, 78°50'13.0"W) ECU3771. (W).

**89. Psorophora (Grabhamia) dimidiata Cerqueira, 1943**.

**Distribution**. Napo: Tena (peninsula known as PALI) (0°59'44.45"S, 77°49'07.31"W) (Jacome et al. 2023). INSPI collection—Morona Santiago: Macas (2°19'28.34"S, 78°7'56.86"W) ECU7206. Sucumbíos: Nueva Loja (0°5'20.00"S, 76°52'9.00"W) ECU3185. Sucumbíos: Shushufindi (0°7'26.46"S, 76°39'2.61"W) ECU9828. (E).

**90. Psorophora (Janthinosoma) albigenu Peryassú, 1908**.

**Synonyms**. *Jan. albigenu* Peryassú, 1908; *Jan. paraguayensis* Strickland, 1911; *Ps. bruchi* Petrocchi, 1927.

**Distribution**. Orellana (formerly Napo): Yasuní National Park, Tiputini Biodiversity Station, Tower (00°38'17"S, 076°08'42"W); Guacamayo Trail, 2,300 m a.s.l. (0°38'2.42"S, 76°9'49.87'W) (Linton et al. 2013). (E).

**91. Psorophora (Janthinosoma) albipes (Theobald, 1907)**.

**Synonym**. *Jan. albipes* Theobald, 1907.

**Distribution**. Sucumbíos (formerly Napo): Cuyabeno, 50 km W of Tarapoa, Topography Camp, Aguas Negras; Orellana (formerly Napo): Coca, confluence of Coca and Napo rivers, 250 m a.s.l. (0°28'00.0"S, 76°58'00.0"W) (Heinemann and Belkin 1979); Orellana: Yasuní National Park, Tiputini Biodiversity Station, Main Camp (0°38'13.51"S, 76°09'0.32"W) (Linton et al. 2013); Imbabura: Lita (0°52'11.83"N, 78°27'05.56"W); Cachaco (0°49'41.19"N, 78°24'03.55"W); Esmeraldas: San Francisco de Bogotá (01°09'21.98"N, 78°40'17.98"W) (Navarro et al. 2015). INSPI collection—Sucumbíos: Shushufindi (0°10'55.4"S, 76°37'32.0"W) ECU19592; Esmeraldas, Eloy Alfaro, Borbón (1°05'07.0"N, 79°00'11.7"W) ECU25591. (E, W).

**92. Psorophora (Janthinosoma) cyanescens (Coquillett, 1902)**.

**Synonyms**. *Cx. cyanescens* Coquillett, 1902; *Ps. purpurascens* Edwards, 1922; *Ps. tovari* Evans, 1922.

**Distribution**. Napo, Orellana, and Pastaza (formerly Napo-Pastaza) (Levi-Castillo 1952b, 1953d); Ecuador (Levi-Castillo 1958). (E).

**93. Psorophora (Janthinosoma) ferox (Humboldt, 1819) †**.

**Synonyms**. *Ae. pazosi* Pazos, 1908; *Cx. ferox* Humboldt, 1819; *Cx. posticatus* Wiedemann, 1821; *Cx. musicus* Say, 1829; *Jan. centrale* Brèthes, 1910; *Jan. coquilletti* Theobald, 1907; *Jan. echinata* Grabham, 1906; *Jan. jamaicensis* Theobald, 1907; *Jan. terminalis* Coquillett, 1906; *Jan. vanhalli* Dyar & Knab, 1906; *Ps. sayi* Dyar & Knab, 1906; *Ps. sayi* Theobald, 1907.

**Distribution**. Type locality: Guayas: flooded Guayaquil River valley near ‘‘Borodon’’, most likely refers to Samborondón (Stone et al. 1959; Belkin 1968); Guayas: 12 km W of Guayaquil on Rt. 3; Guayas: Samborondón, La Bocana estuary, road upstream from town (01°57'12"S, 079°44'7.9"W); Los Ríos, ~ 30 km SW of Quevedo, 3 km S of El Empalme, 100 m a.s.l. (01°06'12"S, 079°41'07.9"W); Esmeraldas; Napo-Pastaza; Orellana (formerly Napo): Coca, confluence of Coca and Napo rivers, 250 m a.s.l. (0°28'00.0"S, 76°58'00.0"W) (Heinemann and Belkin 1979). INSPI collection—Pastaza, Puyo (1°28'38.5"S, 77°59'54.7"W) ECU2955; Sucumbíos: Shushufindi (0°10'55.4"S, 76°37'31.8"W) ECU19593. (E, W).

**94. Psorophora (Janthinosoma) lanei Shannon & Cerqueira, 1943**.

**Distribution**. Orellana (formerly Napo): Coca, confluence of Coca and Napo rivers, 250 m a.s.l. (0°28'00.0"S, 76°58'00.0"W) (Heinemann and Belkin 1979; GBIF-Secretariat 2025); Orellana (formerly Napo): Yasuní National Park, Tiputini Biodiversity Station, Tower (00°38'17"S, 076°08'42"W) (Linton et al. 2013). (E).

**95. Psorophora (Janthinosoma) lutzii (Theobald, 1901)**.

**Synonyms**. *Jan. lutzii* Theobald, 1901; *Ps. chaquensis* Paterson & Shannon, 1927.

**Distribution**. Orellana (formerly Napo): ~ 50 km E of El Coca, ‘‘Isla Pompeya’’ (00°26'12"S, 076°37'07.5"W); Orellana (formerly Napo): Coca, confluence of Coca and Napo rivers, 250 m a.s.l. (00°28'12"S, 076°58'07.5"W) (Heinemann and Belkin 1979; GBIF-Secretariat 2025). (E).

**96. Psorophora (Psorophora) ciliata (Fabricius, 1794)**.

**Synonyms**. *Cx. ciliata* Fabricius, 1794; *Cx. cyanopennis* Humboldt, 1819; *Cx. molestus* Wiedemann, 1820; *Cx. nubilus* Robineau-Desvoidy, 1827; *Cx. tibialis* Robineau-Desvoidy, 1827; *Ps. boscii* Robineau-Desvoidy, 1827; *Ps. ctites* Dyar, 1918; *Ps. lynchi* Brèthes, 1916.

**Distribution**. Orellana (formerly Napo): Coca, confluence of Coca and Napo rivers, 250 m a.s.l. (0°28'00.0"S, 76°58'00.0"W) (GBIF-Secretariat 2025). INSPI collection—Sucumbíos: Shushufindi ECU10703; Manabí: Pacoche (1°04'14.6"S, 80°51'02.9"W) ECU10665. (E, W).

**97. Psorophora (Psorophora) cilipes (Fabricius, 1805)**.

**Synonyms**. *Cx. cilipes* Fabricius, 1805; *Ps. iracunda* Dyar & Knab, 1906; *Sabethes scintillans* Walker, 1848.

**Distribution**. Orellana (formerly Napo): Coca, confluence of Coca and Napo rivers, 250 m a.s.l. (0°28'00.0"S, 76°58'00.0"W) (Heinemann and Belkin 1979). INSPI collection—Napo: Tena, Jatún Sacha Station (1°04'01.1"S, 77°35'53.3"W) ECU3512; Sucumbíos: Shushufindi (0°12'39.2"S, 76°36'58.3"W) ECU19509. (E).

**98. Psorophora (Psorophora) holmbergii Lynch Arribalzaga, 1891**.

**Synonym**. *Ps. agoggylia* Dyar, 1922.

**Distribution**. INSPI collection—Sucumbíos: Pacayacu (0°02'03.0"S, 76°35'30.7"W) ECU12276. (NR, E).

**99. Psorophora (Psorophora) lineata (Humboldt, 1819)**.

**Synonym**. *Ps. blanchardi* Surcouf & Gonzalez-Rincones, 1911.

**Distribution**. Orellana (formerly Napo): Coca, confluence of Coca and Napo rivers, 250 m a.s.l. (0°28'00.0"S, 76°58'00.0"W) (Heinemann and Belkin 1979). (E).

**100. Psorophora (Psorophora) pallescens Edwards, 1922**.

**Distribution**. Ecuador (AFPMB 1998).

### Tribe Culicini (87 species)

**101. Culex (Aedinus) accelerans Root, 1927**.

**Synonym**. *Cx. paraplesia* Dyar, 1922.

**Distribution**. INSPI collection—Sucumbíos: Shushufindi (00°11'32.64"S, 76°37'48.87"W) ECU3227. (NR, E).

**102. Culex (Aedinus) amazonensis (Lutz, 1905)**.

**Synonyms**. *Ads. amazonensis* Lutz, 1905; *Cx. hildebrandi* Evans, 1923; *Eubonnea tapena* Dyar, 1919.

**Distribution**. Orellana (formerly Napo): ~ 50 km E of El Coca, ‘‘Isla Pompeya” (00°26'12"S, 076°37'07.5"W) (GBIF-Secretariat 2025); Los Ríos: Babahoyo (01°48'10.1"S, 079°31'51.6"W) (Berlin and Belkin 1980); Sucumbíos (formerly Napo): Cuyabeno, Tarapoa, 300 m a.s.l. (00°07'21.2"S, 076°20'45.9"W) (Heinemann and Belkin 1979). (E, W).

**103. Culex (Anoedioporpa) bamborum Rozeboom & Komp, 1948**.

**Distribution**. Orellana: Yasuní National Park, near Tiputini River, ~ 8 km from Tiputini Biodiversity Station (00°39'53"S, 076°12'54"W) (Linton et al. 2013). (E).

**104. Culex (Anoedioporpa) browni Komp, 1936**.

**Distribution**. Sucumbíos (formerly Napo): Cuyabeno, Tarapoa, 300 m a.s.l. (00°07'21.2"S, 076°20'45.9"W) (Berlin and Belkin 1980); Orellana: Yasuní National Park, Tiputini Biodiversity Station, Lago Trail, 2,000 m a.s.l. (0°38'15.54"S, 76°9'51.82"W); near Río Tiputini, ~ 8 km from Tiputini Biodiversity Station (00°39'53"S, 076°12'54"W) (Linton et al. 2013). INSPI collection—Esmeraldas: Palestina (1°01'25.8"N, 79°25'45.9"W) ECU19158 (E, W).

**105. Culex (Anoedioporpa) conservator Dyar & Knab, 1906**.

**Synonyms**. *Cx. bifoliata* Dyar, 1922; *Cx. divisior* Dyar & Knab, 1906; *Cx. paganus* Evans, 1923; *Cx. surukumensis* Anduze, 1941.

**Distribution**. Sto. Domingo de los Tsáchilas: Reserva La Perla (00°01'53"S, 79°24'04"W) (GBIF-Secretariat 2025); Orellana, Yasuní National Park, Tiputini Biodiversity Station, Murciélago Trail, 250 m a.s.l. (0°38'13.50"S, 76°8'38.08"W), Lago Trail, 2,000 m a.s.l. (00°38'15.5"S, 076°09'51.8"W) (Linton et al. 2013). INSPI collection— Guayas: Guayaquil ECU20063. (E, W).

**106. Culex (Anoedioporpa) originator Gordon & Evans, 1922**.

**Distribution**. Ecuador (Levi-Castillo 1958).

**107. Culex (Carrollia) babahoyensis Levi-Castillo, 1953†**.

**Distribution**. Type locality: Los Ríos: Juan Montalvo, “Hacienda Mora’’ (Levi-Castillo 1953f, 1953a; Stone et al. 1959); Los Ríos: Montalvo, 30 km E of Babahoyo, “Hacienda Mora”, across river at end of main path of Montalvo (01°49'12"S, 079°31'07.8"W); Los Ríos: Quevedo, 1 km E from Valencia (00°56'46.9"S, 079°20'10.5"W); Los Ríos, ~ 30 km SW of Quevedo, 3 km S of El Empalme, 100 m a.s.l. (01°06'12"S, 079°41'07.9"W) (Levi-Castillo 1953c; Heinemann and Belkin 1979; GBIF-Secretariat 2025); Orellana: Loreto, Bigal River (00°32'11.4"S, 077°25'29"W). INSPI collection—Sucumbíos: Nueva Loja (0°04'30.3"N, 77°13'03.2"W) ECU19837; Sucumbíos: Shushufindi (0°10'53.4"S, 76°37'31.9"W) ECU19631; Orellana, Loreto, Bigal River (0°31'08.0"S, 77°25'40.4"W) ECU10729. (E, W).

**108. Culex (Carrollia) bihaicolus Dyar & Núñez Tovar, 1927**.

**Distribution**. Los Ríos: Quevedo, 1 km E from Valencia, 200 m a.s.l. (00°56'46.9"S, 079°20'10.5"W) (Heinemann and Belkin 1979); Sto. Domingo de los Tsáchilas: Valle Hermoso, at km 24 on the Quinidé-Esmeraldas highway (00°05'10"S, 079°16'21"W) (GBIF-Secretariat 2025); Esmeraldas: Quinindé; Los Ríos: Balzar (00°19'33.2"N, 079°27'58"W) (Valencia 1973). (W).

**109. Culex (Carrollia) bonnei Dyar, 1921**.

**Distribution**. Orellana (formerly Napo), ~ 50 km E of El Coca, ‘‘Isla Pompeya’’ at confluence with Jivino River (00°26'12"S, 076°37'07.5"W) (Valencia 1973); Orellana: Yasuní National Park, Tiputini Biodiversity Station, Lago trail, 1,500 m a.s.l. (00°38'15.5"S, 076°09'51.8"W); Main Camp (0°38'13.51"S, 76°9'0.32"W) (Linton et al. 2013); Esmeraldas: San Francisco de Bogotá (01°09'21.98"N, 78°40'17.98"W) (Navarro et al. 2015). INSPI collection—Esmeraldas, Rioverde (1°04'28.3"N, 79°24'39.3"W) ECU19654. (E, W).

**110. Culex (Carrollia) infoliatus Bonne-Wepster & Bonne, 1920**.

**Distribution**. Pastaza (formerly Napo-Pastaza): Puyo (Valencia 1973). INSPI collection—Orellana, Loreto, Bigal River (0°31'08.0"S, 77°25'40.4"W) ECU10723. (E).

**111. Culex (Carrollia) irridescens Lutz, 1905**.

**Synonym**. *Car. iridescens* Lutz, 1905.

**Distribution**. Orellana: Yasuní National Park, Tiputini Biodiversity Station, Maquisapa Trail, 6,200 m a.s.l. (00°37'54.9"S, 076°09'58.4"W) (Linton et al. 2013). (E).

**112. Culex (Carrollia) metempsytus Dyar, 1921**.

**Distribution**. Napo, Pastaza, and Orellana (formerly Napo-Pastaza); Morona Santiago and Zamora Chinchipe (formerly Santiago-Zamora) (Levi-Castillo 1958). (E).

**113. Culex (Carrollia) secundus Bonne-Wepster & Bonne, 1920**.

**Distribution**. Orellana: Tiputini Biodiversity Station, Tiputini River, 6 km from Tiputini Biodiversity Station (0°39'50.00"S, 76°12'56.01"W) (Linton et al. 2013). (E).

**114. Culex (Carrollia) urichii (Coquillett, 1906)**.

**Synonyms**. *Cx. mathesoni* Anduze, 1942; *Melanoconion urichii* Coquillett, 1906.

**Distribution**. Orellana: Yasuní National Park, Tiputini Biodiversity Station, Harpía Trail, 100 m a.s.l. (0°38'13.5"S, 76°09'00.3"W); Murciélago Trail, 250 m a.s.l. (0°38'13.50"S, 76°8'38.08"W); Lake ~ 4 hectares (00°38'13.51"S, 76°09'0.31"W); Lago Trail, 1,700 m a.s.l. (0°38'14.12"S, 76°9'43.74"W); Lago Trail, 1,500 m a.s.l. (0°38'14.98"S, 76°9'37.53"W) (Linton et al. 2013). (E).

**115. Culex (Carrollia) wilsoni Lane & Whitman, 1943**.

**Distribution**. Orellana: Yasuní National Park, Tiputini Biodiversity Station, Maquisapa Trail, 6,300 m a.s.l. (00°37'54.9"S, 076°09'58.3"W) (Linton et al. 2013). (E).

**116. Culex (Culex) archegus Dyar, 1929**.

**Distribution**. Pichincha: Quito (Bram 1967); Azuay: Cuenca (Levi-Castillo 1953c; Stone et al. 1959); Ecuador (AFPMB 1998; Bánki et al. 2025). (A).

**117. Culex (Culex) articularis Philippi, 1865**.

**Distribution**. Azuay; Cañar; Chimborazo; Loja (Levi-Castillo 1953c); Ecuador (Levi-Castillo 1958; AFPMB 1998). (A).

**118. Culex (Culex) bonnei Dyar & Knab, 1919**.

**Distribution**. Orellana (formerly Napo): Coca, confluence of Coca and Napo rivers, 250 m a.s.l. (0°28'00.0"S, 76°58'00.0"W) (Heinemann and Belkin 1979). (E).

**119. Culex (Culex) camposi Dyar, 1925†**.

**Synonym**. *Cx. coronator camposi*, resurrected from syn coronator Bram, 1967.

**Distribution**. Type locality: Los Ríos, Quevedo, ~ 10 km SW of Quevedo, Pichilingue, 100 m a.s.l. (Heinemann and Belkin 1979); Orellana: Near Tiputini River ~ 8 km from Tiputini Biodiversity Station (0°39'53.00"S, 76°12'54.00"W) (Linton et al. 2013). (E, W).

**120. Culex (Culex) chidesteri Dyar, 1921**.

**Synonyms**. *Cx. deanei* Correa & Ramalho, 1959; *Cx. finlayi* Perez Vigueras, 1956.

**Distribution**. INSPI collection—Manabí: Pacoche ECU12425; Napo: Jatun Sacha (1°05'11.3"S, 77°37'02.0"W) ECU3497. (E).

**121. Culex (Culex) coronator Dyar & Knab, 1906**.

**Synonym**. *Cx. mooseri* Vargas & Martinez Palacios, 1954.

**Distribution**. Guayas: Guayaquil; Durán (Campos 1922, 1925); Zamora Chinchipe: Zamora, ~ 10 km NE of tributary stream of Zamora River; Sucumbíos (formerly Napo): Cuyabeno, ~ 50 km of ‘‘Tarapoa (Main Camp)”; Cuyabeno, ~ 50 km W of ‘‘Tarapoa, Aguarico 3, Contrasa”; Cuyabeno, ~ 50 km W of ‘‘Tarapoa, Campsite 5.5 km N” (Heinemann and Belkin 1979). Orellana: Yasuní National Park: near Tiputini River ~ 8 km from Tiputini Biodiversity Station (00°39'53"S, 076°12'54"W); Main Camp (0°38'13.52"S, 76°9'0.33"W) (Linton et al. 2013). INSPI collection—Pichincha: Caoni (0°00'47.6"N, 79°10'22.7"W) ECU14922; Manabí: Portoviejo (1°04'09.8"S, 80°29'55.5"W) ECU7120; Napo: Jatun Sacha (1°05'11.3"S, 77°37'02.0"W) ECU3419. (E, W).

**122. Culex (Culex) declarator Dyar & Knab, 1906**.

**Synonyms**. *Cx. dictator* Dyar & Knab, 1909; *Cx. forattinii* Correa & Ramalho, 1959; *Cx. inquisitor* Dyar & Knab, 1906; *Cx. jubilator* Dyar & Knab, 1907; *Cx. proclamator* Dyar & Knab, 1906; *Cx. revelator* Dyar & Knab, 1907; *Cx. vindicator* Dyar & Knab, 1909.

**Distribution**. Guayas: Chongón, 24 km W of Guayaquil on Rt. 3 near road to Chongón (02°13'10.5"S, 080°05'05.8"W); Guayaquil, 10 km W on Rt. 3; Esmeraldas: near Atacames (00°52'36"N, 79°50'46.3"W); Orellana: Tarapoa, ~ 50 km W of Cuyabeno (00°07'21.2"S, 076°20'45.9"W) (Heinemann and Belkin 1979). INSPI collection—Manabí: Pacoche ECU12427. (E, W).

**123. Culex (Culex) dolosus (Lynch Arribálzaga, 1891)**.

**Synonyms**. *Cx. bilineatus* Theobald, 1903; *Cx. bonariensis* Brèthes, 1916; *Heteronycha dolosus* Lynch Arribalzaga, 1891.

**Distribution**. Ecuador (Stone et al. 1959; Bram 1967; AFPMB 1998; Bánki et al. 2025).

124. Culex (Culex) guayasi Levi-Castillo, 1953**†. Unrecognized species (Bram 1967).

**Distribution**. Type locality: Babahoyo, Los Ríos (01°48'55.5"S, 079°31"00"W) (Levi-Castillo 1953c, 1953c; GBIF-Secretariat 2025); Ecuador (Levi-Castillo 1958). (W).

**125. Culex (Culex) levicastilloi Lane, 1945†**.

**Synonym**. *Cx. tejerai* Cova García, 1962.

**Distribution**. Type locality: Imbabura, Ibarra, just NE of Laguna de Yahuarcocha, 1,950 m a.s.l. (00°22'46.2"N, 078°05'22.7"W) (Heinemann and Belkin 1979; GBIF-Secretariat 2025). (A).

**126. Culex (Culex) maracayensis Evans, 1923**.

**Synonym**. *Cx. aglischrus* Dyas, 1924.

**Distribution**. Ecuador (Levi-Castillo 1958).

**127. Culex (Culex) mollis Dyar & Knab, 1906**.

**Synonyms**. *Cx. elocutilis* Dyar & Knab, 1909; *Cx. equivocator* Dyar & Knab, 1907; *Cx. lateropunctata* Theobald, 1907; *Cx. lepostenis* Dyar, 1923; *Cx. tisseuli* Senevet, 1937.

**Distribution**. Orellana: Yasuní National Park, Tiputini Biodiversity Station, near Lake (0°38'13.51"S, 76°9'0.32"W); Lago Trail, 2,000 m a.s.l. (0°38'15.54"S, 76°9'51.79"W); Lago Trail, 1,700 m a.s.l. (0°38'14.13"S, 76°9'43.83"W); Lago Trail, 1,500 m a.s.l. (0°38'14.98"S, 76°9'37.53"W); Main Camp (0°38'13.52"S, 76°9'0.33"W); Numa Trail (0°38'15.50"S, 76°8'59.31"W); Chorongo Trail (0°38'11.18"S, 76°8'55.67"W) (Linton et al. 2013); Sto. Domingo de los Tsáchilas (formerly Pichincha): Reserva La Perla (00°01'03"S, 079°23'47"W); Orellana (formerly Napo): Coca, confluence of Coca and Napo rivers, 250 m a.s.l. (0°28'00.0"S, 76°58'00.0"W) (Heinemann and Belkin 1979). INSPI collection—Esmeraldas: Borbón ECU27968. (E, W).

**128. Culex (Culex) nigripalpus Theobald, 1901**.

**Synonyms**. *Cx. azuayus* Levi-Castillo, 1954; *Cx. biocellatus* Theobald, 1903; *Cx. caraibeus* Howard, Dyar & Knab, 1912; *Cx. carmodyae* Dyar & Knab, 1906; *Cx. factor* Dyar & Knab, 1906; *Cx. microsquamosus* Theobald, 1905; *Cx. mortificator* Dyar & Knab, 1906; *Cx. palus* Theobald, 1903; *Cx. prasinopleurus* Martini, 1914; *Cx. proximus* Dyar & Knab, 1909; *Cx. regulator* Dyar & Knab, 1906; *Cx. similis* Theobald, 1903; *Trichopronomyia microannulata* Theobald, 1907.

**Distribution**. Guayas: Chongón, 24 km W of Guayaquil on Rt. 3 near road to Chongón (02°13'10.5"S, 080°05'05.8"W); Guayas: Guayaquil, 10 km W of Guayaquil on Rt. 3 (2°11'20.69"S, 79°57'56.99"W); Guayas: Guayaquil, airport (2°9'42.79"S, 79°53'5.73"W); Manabí: Manta; Guayas: Guayaquil, Febres Cordero (2°12'2.89"S, 79°54'55.69"W); Guayas: Guayaquil, Atarazana (2°10'47.92"S, 79°53'5.68"W) (Heinemann and Belkin 1979; GBIF-Secretariat 2025); Sto Domingo de los Tsáchilas: Reserva La Perla (0°1'3.00"S, 79°23'47.00"W); Orellana (formerly Napo): Coca, confluence of Coca and Napo rivers, 250 m a.s.l. (0°28'00.0"S, 76°58'00.0"W) (GBIF-Secretariat 2025). INSPI collection—Sucumbíos: Shushufindi (0°10'19.6"S, 76°38'18.7"W) ECU14855; (Sucumbíos: Nueva Loja) (0°06'04.2"N, 76°52'47.8"W) ECU14114; Manabí: Santa Ana (1°09'16.0"S, 80°16'59.8"W) ECU13696; Manabí: Bahía de Caráquez (0°38'33.5"S, 80°24'36.0"W) ECU13667; Manabí: Río Seco (1°07'37.5"S, 80°50'33.0"W) ECU10581; Pichincha: Sangolquí (0°21'18.9"S, 78°25'29.8"W) ECU13554; Napo: Jatun Sacha (1°05'11.3"S, 77°37'02.0"W) ECU3484. (E, W).

**129. Culex (Culex) quinquefasciatus Say, 1823**.

**Synonyms**. *Cx. acer* Walker, 1848; *Cx. aestuans* Wiedemann, 1828; *Cx. aikenii* Dyar & Knab, 1908; *Cx. albolineatus* Giles, 1901; *Cx. anxifer* Bigot, 1859; *Cx. aseyehae* Dyar & Knab, 1915; *Cx. autumnalis* Weyenbergh, 1882; *Cx. barbarus* Dyar & Knab, 1906; *Cx. cartroni* Ventrillon, 1905; *Cx. christophersii* Theobald, 1907; *Cx. cingulatus* Doleschall, 1856; *Cx. cubensis* Bigot, 1857; *Cx. didieri* Neveu-Lemaire, 1906; *Cx. fatigans* Wiedemann, 1828; *Cx. fouchowensis* Theobald, 1901; *Cx. hensemaeon* Dyar, 1920; *Cx. luteoannulatus* Theobald, 1901; *Cx. macleayi* Skuse, 1889; *Cx. minor* Theobald, 1908; *Cx. nigrirostris* Enderlein, 1920; *Cx. pallidocephala* Theobald, 1904; *Cx. penafieli* Sanchez, 1885; *Cx. pungens* Wiedemann, 1828; *Cx. pygmaeus* Neveu-Lemaire, 1906; *Cx. quasilinealis* Theobald, 1907; *Cx. quasipipiens* Theobald, 1901; *Cx. raymondii* Tamayo, 1907; *Cx. reesi* Theobald, 1901; *Cx. revocator* Dyar & Knab, 1909; *Cx. sericeus* Theobald, 1901; *Cx. serotinus* Philippi, 1865; *Cx. skusii* Giles, 1900; *Cx. trillineatus* Theobald, 1901; *Cx. zeltneri* Neveu-Lemaire, 1906; *Culicelsa fuscus* Taylor, 1914.

**Distribution**. Guayas: Daule, ~ 10 km S of El Triunfo (01°56'12"S, 079°58'07.9"W); Guayas: Guayaquil, airport (02°09'54.7"S, 079°53'13.8"W); Orellana (formerly Napo): Coca, confluence of Coca and Napo rivers, 250 m a.s.l. (0°28'00.0"S, 76°58'00.0"W) (Heinemann and Belkin 1979; GBIF-Secretariat 2025); Galápagos Islands (restricted to human settlements): Baltra, Isabela, Santa Cruz, San Cristóbal (Whiteman et al. 2005; Bataille et al. 2009; Siers et al. 2010; Eastwood et al. 2013); Imbabura: Lita (0°52'11.83"N, 78°27'05.56"W); Cachaco (0°49'41.19"N, 78°24'03.55"W); Esmeraldas: San Francisco de Bogotá (01°09'21.98"N, 78°40'17.98"W) (Navarro et al. 2015); Cosmotropical (Stone et al. 1959). Cosmotropical (Bánki et al. 2025). INSPI collection—Guayas: Guayaquil (2°07'21.5"S, 79°58'03.6"W) ECU19978; Napo: Quijos (0°26'28.4"S, 77°52'39.9"W) ECU16353; Sucumbíos: Shushufindi (0°10'20.6"S, 76°38'04.7"W) ECU14859; Esmeraldas: Esmeraldas (0°57'07.5"N, 79°39'10.1"W) ECU13849; Pichincha: Llano Chico (0°07'55.4"S, 78°26'04.7"W) ECU13698; Imbabura: Ibarra (0°20'20.8"N, 78°07'20.0"W) ECU13153; Galápagos: Santa Cruz (0°40'11.8"S, 90°16'26.4"W) ECU12905; Galápagos: San Cristóbal (0°54'47.4"S, 89°34'44.6"W) ECU12890; Manabí: Pacoche ECU12425; Cotopaxi: Pujilí (0°56'08.9"S, 78°40'43.3"W) ECU29447; Esmeraldas: San José de Cayapas (0°51'21.2"N, 78°58'10.1"W) ECU29114. (E, W, A, G).

130. Culex (Culex) quitensis Levi-Castillo, 1953**†. Unrecognized species (Bram 1967).

**Synonym**. *Cx. nigripalpus*Bram 1967.

**Distribution**. Type locality: Pichincha, Quito (Levi-Castillo 1953d); Cotopaxi; Pichincha; Imbabura (Levi-Castillo 1953c); Ecuador (Levi-Castillo 1958; AFPMB 1998; Bánki et al. 2025). (A).

**131. Culex (Culex) stenolepis Dyar & Knab, 1908**.

**Distribution**. Pichincha: Pululahua Geobotanical Reserve, Pululahua Volcano (0°03'28.9"N, 78°30'29.1"W) (Navarro et al. 2015). (A).

**132. Culex (Culex) stigmatosoma Dyar, 1907**.

**Synonyms**. *Cx. eumimetes* Dyar & Knab, 1908.

**Distribution**. INSPI collection—Manabí: San Lorenzo, Pacoche Wildlife Refuge (1°4'14.74"S, 80°53'26.99"W) ECU12428, ECU12429, ECU12431, ECU12432. (NR, W).

**133. Culex (Culex) thriambus Dyar, 1921**.

**Synonyms**. *Cx. affinis* Adams, 1903; *Cx. peus* Speiser, 1904; *Cx. eumimetes* Dyar & Knab, 1908.

**Distribution**. Manabí: San Lorenzo, Pacoche Wildlife Refuge (1°7'38.86"S, 80°50'35.48"W) ECU10558. (NR, W).

**134. Culex (Culex) usquatissimus Dyar, 1922**.

**Synonym**. *Cx. coronator*Bram 1967.

**Distribution**. Orellana: Yasuní National Park, Tiputini Biodiversity Station (Linton et al. 2013). (E).

**135. Culex (Culex) usquatus Dyar, 1918**.

**Distribution**. Orellana: Yasuní National Park, Tiputini Biodiversity Station (0°39'53.00"S, 76°12'54.00"W) (Linton et al. 2013). (E).

**136. Culex (Melanoconion) adamesi Sirivanakarn & Galindo, 1980**.

**Distribution**. Esmeraldas: Hdq. de Jesús, Quiñónez, Quinindé (00°20'48.4"N, 079°28'35"W); Guayas: San Rafael, Tenguel (02°58'12.4"S, 079°46'20.3"W) (Sirivanakarn and Galindo 1980). (W).

**137. Culex (Melanoconion) albinensis Bonne-Wepster & Bonne, 1920**.

**Synonyms**. *Cx. gordoni* Evans, 1924; *Cx. maroniensis* Bonne-Wepster & Bonne, 1919.

**Distribution**. Ecuador (Levi-Castillo 1958).

**138. Culex (Melanoconion) bastagarius Dyar & Knab, 1906**.

**Synonyms**. *Cx. alfaroi* Dyar, 1921; *Cx. cuclyx* Dyar & Shannon, 1924; *Cx. innominatus* Evans, 1924; *Cx. thomasi* Evans, 1924; *Cx. vapulans* Dyar, 1920; *Cx. xivylis* Dyar, 1920.

**Distribution**. Pastaza: Puyo, 1.5 km S (01°28'01.4"S, 077°59'1.5"W); Napo: Tena, 13 km SW; Sto. Domingo de los Tsáchilas (formerly Pichincha): Valle Hermoso, Hostería “Valle Hermoso” (00°05'17"S, 079°16'34"W) (GBIF-Secretariat 2025). (E, W).

**139. Culex (Melanoconion) batesi Rozeboom & Komp, 1948**.

**Distribution**. Napo: Tena (0°59'48.0"S, 77°48'49.3"W) (Pecor et al. 1992; GBIF-Secretariat 2025). (E).

**140. Culex (Melanoconion) comatus Senevet & Abonnenc, 1939**.

**Distribution**. Ecuador (Levi-Castillo 1958).

**141. Culex (Melanoconion) comminutor Dyar, 1920**.

**Distribution**. Ecuador (Stone et al. 1959; Pecor et al. 1992; AFPMB 1998; Bánki et al. 2025).

**142. Culex (Melanoconion) conspirator Dyar & Knab, 1906**.

**Synonyms**. *Cx. dysmathes* Dyar & Ludlow, 1921; *Cx. fatuator* Dyar & Shannon, 1924; *Cx. holoneus* Dyar, 1935; *Cx. inducens* Root, 1928; *Cx. macaronensis* Dyar & Nunez Tovar, 1926; *Cx. merodaemon* Dyar, 1921; *Cx. meroneus* Dyar, 1925; *Cx. pasadaemon* Dyar, 1921.

**Distribution**. Guayas: Guayaquil, 10 km W of Rt. 3 (02°11'32.6"S, 079°58'05"W) (Heinemann and Belkin 1979). INSPI collection—Esmeraldas: Santa María de los Cayapas (0°51'24.3"N, 78°58'06.7"W) ECU28283. (W).

**143. Culex (Melanoconion) dunni Dyar, 1918**.

**Synonyms**. *Cx. exedrus* Root, 1927; *Cx. ruffinis* Dyar & Shannon, 1924.

**Distribution**. Napo: Tena, 32 km S (01°16'01.4"S, 077°49'01.5"W); Sucumbíos: Limoncocha Lake (0°23'39.8"S, 76°37'01.2"W) (GBIF-Secretariat 2025). (E).

**144. Culex (Melanoconion) distinguendus Dyar, 1928**.

**Distribution**. Napo, Pastaza, and Orellana (formerly Napo-Pastaza); Morona Santiago and Zamora Chinchipe (formerly Santiago-Zamora) (Levi-Castillo 1949b, 1953d); Ecuador (Levi-Castillo 1958; Pecor et al. 1992; AFPMB 1998). (E).

**145. Culex (Melanoconion) eastor Dyar, 1920**.

**Synonym**. *Cx. manaosensis* Evans, 1924.

**Distribution**. Orellana (formerly Napo): Coca, confluence of Coca and Napo rivers, 250 m a.s.l. (0°28'00.0"S, 76°58'00.0"W) (Heinemann and Belkin 1979; GBIF-Secretariat 2025); Orellana: Yasuní National Park, Tiputini Biodiversity Station, near Lake (00°38'08.2"S, 076°09'53.5"W) (Linton et al. 2013). (E).

**146. Culex (Melanoconion) educator Dyar & Knab, 1906**.

**Synonyms**. *Cx. aneles* Dyar & Ludlow, 1922; *Cx. apeteticus* Howard, Dyar & Knab, 1912; *Cx. bibulus* Dyar, 1920; *Cx. keenani* Galindo & Mendez, 1961.

**Distribution**. Los Ríos: Babahoyo, 5 km W of Babahoyo, 50 m a.s.l. (Stone et al. 1959; Heinemann and Belkin 1979). (E).

**147. Culex (Melanoconion) eknomios Forattini & Sallum, 1992**.

**Distribution**. Sucumbíos: Limoncocha (00°24'37.8"S, 076°37'26.4"W) (Forattini and Sallum 1992). (E).

**148. Culex (Melanoconion) elevator Dyar & Knab, 1906**.

**Synonyms**. *Cx. bonneti* Senevet, 1938; *Cx. curryi* Dyar, 1926; *Cx. dornarum* Dyar & Shannon, 1924; *Cx. vogelsangi* Anduze, 1948.

**Distribution**. Orellana: Yasuní National Park, Tiputini Biodiversity Station, Harpía Trail, 1,500 m a.s.l. (0°37'32.30"S, 76°8'52.03"W) (0°38'8.99"S, 76°9'34.99"W); Harpía Trail, 800 m a.s.l. (0°37'48.64"S, 76°8'43.81"W); Maquisapa trail, 6,300 m a.s.l. (00°37'54.9"S, 076°09'58.3"W) (Linton et al. 2013). (E).

**149. Culex (Melanoconion) ensiformis Bonne-Wepster & Bonne, 1920**.

**Distribution**. Sto. Domingo de los Tsáchilas (formerly Pichincha): Valle Hermoso, next to Hostería “Valle Hermoso” (00°05'17"S, 079°16'34"W) (GBIF-Secretariat 2025). (W).

**150. Culex (Melanoconion) erraticus (Dyar & Knab, 1906)**.

**Distribution**. Guayas, Guayaquil: Entrada Atarazana (2°12'00.0"S, 79°54'00.0"W); Esmeraldas: Propicia 1 (0°54'00.0"N, 79°42'00.0"W) (GBIF-Secretariat 2025). INSPI collection—Esmeraldas: Borbón (1°05'55.4"N, 78°59'10.8"W) ECU28419. (W).

**151. Culex (Melanoconion) evansae Root, 1927**.

**Distribution**. Orellana (formerly Napo): ~ 50 km E of El Coca, ‘‘Isla Pompeya’’ (island in Napo River at confluence with Jivino River) (00°26'12"S, 076°37'07.5"W); Bigal (00°32'11.4"S, 077°25'29"W) (Heinemann and Belkin 1979; GBIF-Secretariat 2025); Orellana: Yasuní National Park, Tiputini Biodiversity Station (0°38'8.99"S, 76°9'34.96"W), Guacamayo Trail, 2,300 m a.s.l. (0°38'2.42"S, 76°9'49.87"W) (Linton et al. 2013). (E).

**152. Culex (Melanoconion) gnomatos Sallum, Hutchings, Leila & Ferreira, 1997**.

**Distribution**. Ecuador (AFPMB 1998).

**153. Culex (Melanoconion) inhibitator Dyar & Knab, 1906**.

**Synonym**. *Cx. investigator* Dyar & Knab, 1906.

**Distribution**. Napo, Pastaza, and Orellana (formerly Napo-Pastaza); Morona Santiago and Zamora Chinchipe (formerly Santiago-Zamora) (Levi-Castillo 1949b, 1953d); Ecuador (Levi-Castillo 1958). (E).

**154. Culex (Melanoconion) innovator Evans, 1924**.

**Distribution**. Ecuador (Levi-Castillo 1958).

**155. Culex (Melanoconion) iolambdis Dyar, 1918**.

**Distribution**. Orellana: Yasuní National Park, Tiputini Biodiversity Station, Harpía Trail, 750 m a.s.l. (00°37'50.2"S, 076°08'43.4"W) (Linton et al. 2013). (E).

**156. Culex (Melanoconion) lucifugus Komp, 1936**.

**Distribution**. Orellana (formerly Napo), ~ 50 km E of El Coca, “Isla Pompeya” (island in Napo River at confluence with Jivino River) (00°26'12"S, 076°37'07.5"W) (Heinemann and Belkin 1979). (E).

**157. Culex (Melanoconion) madininensis Senevert, 1936**.

**Distribution**. Ecuador (Levi-Castillo 1958).

**158. Culex (Melanoconion) ocossa Dyar & Knab, 1919**.

**Distribution**. Sucumbíos: Limoncocha (00°24'1.4"S, 076°38'1.5"W) (GBIF-Secretariat 2025). (E).

**159. Culex (Melanoconion) oedipus Root, 1927**.

**Distribution**. Esmeraldas: San Lorenzo (01°17'21.9"N, 078°50'9.0"W) (Pecor et al. 1992). (W).

**160. Culex (Melanoconion) paracrybda Komp, 1936**.

**Distribution**. Esmeraldas: on road to Muisne (00°37'35.9"N, 80°00'26.7"W) (GBIF-Secretariat 2025). (W).

**161. Culex (Melanoconion) pedroi Sirivanakarn & Belkin, 1980**.

**Distribution**. Orellana (formerly Napo): Coca; Esmeraldas: San Lorenzo (01°17'21.9"N, 078°50'9.0"W) (Berlin and Belkin 1980). (E, W).

**162. Culex (Melanoconion) phlogistus Dyar, 1920**.

**Distribution**. Los Ríos (Levi-Castillo 1953c); Ecuador (Levi-Castillo 1958). (W).

**163. Culex (Melanoconion) plectoporpe Root, 1927**.

**Distribution**. Ecuador (Levi-Castillo 1958).

**164. Culex (Melanoconion) penai Sirivanakarn, 1979**.

**Distribution**. Orellana (formerly Napo): Coca, confluence of Coca and Napo rivers, 250 m a.s.l. (0°28'00.0"S, 76°58'00.0"W) (Sirivanakarn and Galindo 1980). (E).

**165. Culex (Melanoconion) pilosus Lee, 1946**.

**Synonyms**. *Cx. agitator* Dyar & Knab, 1907; *Cx. colombiensis* Dyar, 1924; *Cx. curopiensis* Bonne-Wepster & Bonne, 1919; *Cx. deceptor* Dyar & Knab, 1909; *Cx. hesitator* Dyar & Knab, 1907; *Cx. ignobilis* Dyar & Knab, 1909; *Cx. mastigia* Howard, Dyar & Knab, 1912; *Cx. radiatus* Senevet & Abonnenc, 1939; *Cx. reductor* Dyar & Knab, 1909; *Mochlostyrax cubensis* Dyar & Knab, 1906; *M. floridanus* Dyar & Knab, 1906; *M. jamaicensis* Grabham, 1906; *M. pilosus* Dyar & Knab, 1906.

**Distribution**. Manabí “Bahía, Floresta” (Heinemann and Belkin 1979); Orellana: Yasuní National Park, Tiputini Biodiversity Station, near Tiputini River ~ 8 km from Tiputini Biodiversity Station (0°39'53.00"S, 76°12'54.00"W); Napo: Tena, 11.7 km SW (01°10'1.4"S, 077°53'1.5"W); Pastaza: Puyo, 4 km NE, (01°28'1.4"S, 077°59'1.5"W); Pastaza: Puyo, 23 km SE (01°35'01.4"S, 077°50'01.5"W); Sucumbíos (formerly Napo): Limoncocha (00°24'37.8"S, 076°37'26.4"W) (GBIF-Secretariat 2025). INSPI collection—Guayas: Guayaquil (2°09'17.1"S, 79°53'09.9"W) ECU13151. Napo: Jatun Sacha (1°05'11.3"S, 77°37'02.0"W) ECU3499. (E, W).

**166. Culex (Melanoconion) psatharus Dyar, 1920**.

**Distribution**. Guayas: Posorja, just S of Punta del Morro (02°44'7.2"S, 80°15'19.8"W) (Heinemann and Belkin 1979; GBIF-Secretariat 2025). (W).

**167. Culex (Melanoconion) putumayensis Matheson, 1934**.

**Synonym**. *Cx. cavernicola* Floch & Abonnenc, 1945.

**Distribution**. Orellana (formerly Napo): Coca, ~ 50 km E of “Isla Pompeya” (island in Napo River at confluence with Jivino River) (00°26'12"S, 076°37'07.5"W) (Heinemann and Belkin 1979; GBIF-Secretariat 2025). (E).

**168. Culex (Melanoconion) saramaccensis Bonne-Wepster & Bonne, 1920**.

**Distribution**. Napo, Pastaza, and Orellana (formerly Napo-Pastaza); Morona Santiago and Zamora Chinchipe (formerly Santiago-Zamora) (Levi-Castillo 1949b); Ecuador (Pecor et al. 1992; AFPMB 1998). (E).

**169. Culex (Melanoconion) serratimarge Root, 1927**.

**Distribution**. Orellana: Yasuní National Park, Tiputini Biodiversity Station, Lake ~ 4 hectares (0°38'13.52"S, 76°9'0.33"W) (Linton et al. 2013). (E).

**170. Culex (Melanoconion) spissipes (Theobald, 1903)**.

**Synonyms**. *Cx. alvarezi* Sutil Oramas, Pulido Florenzano & Amarista Meneses, 1987; *Cx. fur* Dyar & Knab, 1907; *Cx. haynei* Komp & Curry, 1932; *Cx. menytes* Dyar, 1918; *Melanoconion spissipes* Theobald, 1903.

**Distribution**. Sucumbíos (formerly Napo): Cuyabeno, ~ 50 km W of Tarapoa, Campsite 800–1000 m south of the trail”, 300 m a.s.l. (Heinemann and Belkin 1979). (E).

**171. Culex (Melanoconion) sursumptor Dyar, 1924**.

**Synonym**. *Cx. ligator* Dyar, 1924.

**Distribution**. Pastaza: Puyo, 16 km NE (01°23'1.4"S, 077°53'1.5"W); Sucumbíos: Limoncocha, other side of Limoncocha Lake (GBIF-Secretariat 2025). (E).

**172. Culex (Melanoconion) symbletos Sallum & Hutchings, 2003**.

**Distribution**. Orellana: Yasuní National Park, Tiputini Biodiversity Station, Lake ~4 hectares (0°38'13.52"S, 76°9'0.33"W) (Linton et al. 2013). (E).

**173. Culex (Melanoconion) taeniopus Dyar & Knab, 1907**.

**Synonyms**. *Cx. opisthopus* Komp, 1926; *Mel. annulipes* Theobald, 1907; *Cx. mychonde* Komp, 1928.

**Distribution**. Orellana (formerly Napo): Coca, confluence of Coca and Napo rivers, 250 m a.s.l. (0°28'00.0"S, 76°58'00.0"W) (GBIF-Secretariat 2025). (E).

**174. Culex (Melanoconion) theobaldi (Lutz, 1904)**.

**Synonyms**. *Cx. chrysonotum* Dyar & Knab, 1908; *Mel. theobaldi* Lutz, 1904; *Neomelanoconion chrysothorax* Newstead & Thomas, 1910.

**Distribution**. Sucumbíos: Limoncocha (00°24'37.8"S, 076°37'26.4"W); Sucumbíos: Cuyabeno, ~ 50 km W of Tarapoa (Main Camp); Esmeraldas: La Propicia 1 (00°56'0.2"N, 079°39'28.6"W) (GBIF-Secretariat 2025); Orellana: Yasuní National Park, Tiputini Biodiversity Station, Lake ~ 4 hectares (0°38'13.52"S, 76°9'0.33"W); Tiputini River, blackwater area ~3.5 km from Tiputini Biodiversity Station (0°38'18.00"S, 76°10'55.00"W); Main Camp (0°38'13.52"S, 76°9'0.33"W) (Linton et al. 2013). INSPI collection—Esmeraldas: Borbón ECU28063. (E, W).

**175. Culex (Melanoconion) vomerifer Komp, 1932**.

**Distribution**. Esmeraldas: San Lorenzo, 14 km from ‘‘La Chiquita,’’ near sea level (01°13'41.9"N078°45'55"W) (Heinemann and Belkin 1979); Orellana: Yasuní National Park, Tiputini Biodiversity Station, Main Camp (0°38'13.52"S, 76°9'0.33"W) (Linton et al. 2013). INSPI collection—Esmeraldas: Borbón (1°05'24.4"N, 78°59'32.4"W) ECU28261. (E, W).

**176. Culex (Microculex) chryselatus Dyar & Knab, 1919**.

**Distribution**. Orellana: Yasuní National Park, Tiputini Biodiversity Station, Tower (00°38'17"S, 076°08'42"W) (Linton et al. 2013; GBIF-Secretariat 2025). INSPI collection—Esmeraldas: Alto Tambo (0°55'49.00"N, 78°32'37.00"W) (Navarro et al. 2015). (E, W).

**177. Culex (Microculex) imitator Theobald, 1903**.

**Synonyms**. *Cx. daumasturus* Dyar & Knab, 1906; *Cx. vector* Dyar & Knab, 1906; *Microculex argenteoumbrosus* Theobald, 1907.

**Distribution**. Guayas; Los Ríos; Napo, Pastaza, and Orellana (formerly Napo-Pastaza); Morona Santiago and Zamora Chinchipe (formerly Santiago-Zamora) (Levi-Castillo 1949b, 1953d); Ecuador (Levi-Castillo 1958; Stone et al. 1959; Bánki et al. 2025). (E, W).

**178. Culex (Microculex) neglectus Lutz, 1904**.

**Distribution**. Esmeraldas: Alto Tambo (0°55'49.00"N, 78°32'37.00"W) (Navarro et al. 2015). (W).

**179. Culex (Microculex) pleuristriatus Theobald, 1903**.

**Distribution**. Orellana: Yasuní National Park, Tiputini Biodiversity Station, Tower (0°38'17.0"S, 76°08'42.0"W); Main Camp (0°38'13.5"S, 76°09'00.3"W) (Linton et al. 2013). (E).

**180. Culex (Microculex) stonei Lane & Whitman, 1943**.

**Distribution**. Orellana: Yasuní National Park, Tiputini Biodiversity Station, Tower (0°38'17.0"S, 76°08'42.0"W) (Linton et al. 2013). (E).

**181. Culex (Phenacomyia) corniger Theobald, 1903**.

**Synonyms**. *Cx. basilicus* Dyar & Knab, 1906; *Cx. hassardii* Grabham, 1906; *Cx. loquaculus* Dyar & Knab, 1909; *Cx. rigidus* Senevet & Abonnenc, 1939; *Cx. subfuscus* Theobald, 1907.

**Distribution**. Orellana (formerly Napo), ~ 50 km E of El Coca, “Isla Pompeya” (island in Napo River at confluence with Jivino River) (00°26'12"S, 076°37'07.5"W); Esmeraldas: Burrera Island (0°55'00.0"N, 79°39'11.0"W) (GBIF-Secretariat 2025); Sucumbíos (formerly Napo), Cuyabeno, ~ 50 km W of ‘‘Tarapoa, Campsite 800–1000 m south of the trail”, 300 m a.s.l. (Heinemann and Belkin 1979); Imbabura: Lita (0°52'11.8"N, 78°27'05.6"W); Esmeraldas: San Francisco de Bogotá (01°09'21.98"N, 78°40'17.98"W); Esmeraldas: Durango (1°00'40.87"N, 78°36'03.85"W); Carchi: El Baboso (0°53'47.05"N, 78°26'34.98"W) (Navarro et al. 2015). INSPI collection—Manabí: Pacoche ECU12433; Esmeraldas: Esmeraldas ECU6112. (E, W).

**182. *Culex maculatus*Belkin 1968†. Nomen dubium**

**Distribution**. Type locality: Los Ríos, Babahoyo (1°48'10.1"S, 79°31'51.6"W) (Stone et al. 1959; Belkin 1968). (W).

**183. *Culex ocellatus* Theobald, 1903. Uncertain subgenus**.

**Synonyms**. *Cx. automartus* Root, 1927.

**Distribution**. Guayas: Samborondón (GBIF-Secretariat 2025). (W).

**184. *Deinocerites pseudes* Dyar & Knab, 1909**.

**Distribution**. Guayas: Playas, 7 km SE of Playas on road to Posorja (2°40'52.0"S, 80°21'16.2"W) (Heinemann and Belkin 1979; GBIF-Secretariat 2025). INSPI collection—Esmeraldas: Rioverde (1°03'03.9"N, 79°24'29.8"W) ECU8451. (W).

**185. *Galindomyia leei* Stone & Barreto, 1969**.

**Distribution**. Manabí: Bahía de Caráquez (0°37'47.9"S, 80°25'28.3"W) (Adames and Arzube 1975). (W).

**186. Lutzia (Lutzia) allostigma (Howard, Dyar & Knab, 1915)**.

**Distribution**. Orellana: Yasuní National Park, Tiputini Biodiversity Station, near Lake (0°38'13.5"S, 76°09'00.3"W); Lago Trail, 1,500 m a.s.l. (0°38'14.98"S, 76°9'37.53"W); Main Camp (0°38'13.52"S, 76°9'0.33"W); near Main Camp (022'15.14"S, 76°9'0.32"W) (Linton et al. 2013). INSPI collection—Morona Santiago: Macas (2°28'08.6"S, 78°10'16.6"W) ECU16518; Sucumbíos: General Farfán (0°38'13.52"S, 76°9'0.33"W) ECU7310. (E).

**187. Lutzia (Lutzia) bigoti Bellardi, 1862**.

**Distribution**. Ecuador (Levi-Castillo 1958).

### Tribe Mansoniini (11 species)

**188. Coquillettidia (Rhynchotaenia) albicosta Peryassú, 1908**.

**Synonym**. *Taeniorhynchus albicosta* Peryassú, 1908.

**Distribution**. Sucumbíos: Shushufindi, La Unión neighborhood (00°09'28.72"S, 76°38’ 53.05"W) ECU10696. (NR, E).

**189. Coquillettidia (Rhynchotaenia) arribalzagae (Theobald, 1903)**.

**Synonyms**. *Taeniorhynchus arribalzagae* Theobald, 1903; *Taeniorhynchus coticula* Dyar & Knab, 1907.

**Distribution**. Orellana (formerly Napo): Coca, confluence of Coca and Napo rivers, 250 m a.s.l. (0°28'00.0"S, 76°58'00.0"W); Orellana (formerly Napo): Coca, ~ 50 km E of ‘‘Isla Pompeya’’ (island in Napo River at confluence with Jivino River) (0°26'12.0"S, 76°37'07.5"W) (Heinemann and Belkin 1979; GBIF-Secretariat 2025). (E).

**190. Coquillettidia (Rhynchotaenia) fasciolata (Lynch Arribálzaga, 1891)**.

**Synonym**. *Taeniorhynchus fasciolata* Lynch Arribalzaga, 1891.

**Distribution**. Orellana (formerly Napo): Coca, confluence of Coca and Napo rivers, 250 m a.s.l. (0°28'00.0"S, 76°58'00.0"W); ~ 50 km E of El Coca, “Isla Pompeya” (island in Napo River at confluence with Jivino River) (0°26'12.0"S, 76°37'07.5"W) (GBIF-Secretariat 2025); Sucumbíos (formerly Napo): Cuyabeno, 50 km of “Tarapoa, Campsite 800–1000 m south of the trail” (Heinemann and Belkin 1979). (E).

**191. Coquillettidia (Rhynchotaenia) lynchi (Shannon, 1931)**.

**Synonym**. *Ma. lynchi* Shannon, 1931.

**Distribution**. Orellana (formerly Napo): Coca, confluence of Coca and Napo rivers, 250 m a.s.l. (0°28'00.0"S, 76°58'00.0"W); Orellana (formerly Napo), ~ 50 km E of El Coca, ‘‘Isla Pompeya’’ (island in Napo River at confluence with Jivino River) (0°26'12.0"S, 76°37'07.5"W) (Heinemann and Belkin 1979; GBIF-Secretariat 2025). INSPI collection—Zamora Chinchipe: Zamora ECU8051; Esmeraldas, Eloy Alfaro, Borbón (1°05'24.4"N, 78°59'32.4"W) ECU27888. (E, W).

**192. Coquillettidia (Rhynchotaenia) nigricans (Coquillett, 1904)**.

**Synonyms**. *Bancroftia persephassa* Dyar & Knab, 1909; *Taeniorhynchus nigricans* Coquillett, 1904.

**Distribution**. INSPI collection—Sucumbíos: Nueva Loja (0°04'12.3"N, 76°52'32.0"W) ECU14109; Manabí: Montecristi (1°07'25.0"S, 80°50'43.9"W) ECU11436. (E).

**193. Coquillettidia (Rhynchotaenia) venezuelensis (Theobald, 1912)**.

**Synonyms**. *Ma. araozi* Shannon & Ponte, 1928; *Pseudotaeniorhynchus venezuelensis* Theobald, 1912.

**Distribution**. Pastaza: Puyo (Gutiérrez-Vera et al. 2021). INSPI collection—Esmeraldas: Borbón ECU27609. (E, W).

**194. Mansonia (Mansonia) humeralis Dyar & Knab, 1916**.

**Distribution**. INSPI collection—Manabí: Boca de Palmito (0°05'16.4"S, 79°43'42.0"W) ECU19100; Guayas: Guayaquil (2°16'49.3"S, 79°54'14.6"W) ECU6104. (W).

**195. Mansonia (Mansonia) indubitans Dyar & Shannon, 1925**.

**Distribution**. Manabí: “Bahía, Floresta” (Heinemann and Belkin 1979). (W).

**196. Mansonia (Mansonia) pseudotitillans Theobald, 1901**.

**Synonym**. *Panoplites pseudotitillans* Theobald, 1901.

**Distribution**. INSPI collection—Esmeraldas: Esmeraldas (0°57'54.0"N, 79°39'24.3"W) ECU13848. (W).

**197. Mansonia (Mansonia) titillans (Walker, 1848)**.

**Synonym**. *Cx. titillans* Walker, 1848.

**Distribution**. INSPI collection—Guayas: Guayaquil (2°09'18.5"S, 79°53'09.8"W) ECU10715; Zamora: Zamora (4°07'44.6"S, 78°59'44.3"W) ECU8094; Esmeraldas: Borbón (1°05'24.4"N, 78°59'32.4"W) ECU28282. (E, W).

**198. Mansonia (Mansonia) wilsoni (Barreto & Coutinho, 1944)**.

**Synonym**. *Taeniorhynchus wilsoni* Barreto & Coutinho, 1944.

**Distribution**. Orellana: Yasuní National Park, Tiputini Biodiversity Station, Maquisapa Trail, 6,300 m a.s.l. (0°37'54.9"S, 76°09'58.3"W) (Linton et al. 2013). INSPI collection—Los Ríos: Baba (1°46'51.7"S, 79°40'25.0"W) ECU16544. (E, W).

### Tribe Orthopodomyiini (3 species)

**199. *Orthopodomyia albicosta* (Lutz, 1904)**.

**Synonym**. *Bancroftia albicosta* Lutz, 1904.

**Distribution**. INSPI collection—Morona Santiago: Macas (02°20'17.30"S, 078°9'46.63"W) ECU11574, ECU11586, ECU11590. (NR, E).

**200. *Orthopodomyia fascipes* (Coquillett, 1906)**.

**Synonyms**. *Mansonia bacigalupoi* Martinez & Prosen, 1958; *Ma. fascipes* Coquillett, 1906; *Ma. longipalpis* Newstead & Thomas, 1910; *Ma. townsendi* Lima, 1935.

**Distribution**. Orellana: Yasuní National Park, Tiputini Biodiversity Station, Canopy walkway (0°38'14.9"S, 76°08'36.7"W); Maquisapa Trail, 6,200 m a.s.l. (00°37'54.9"S, 076°09'58.4"W) (Linton et al. 2013); Orellana (formerly Napo): Coca, confluence of Coca and Napo rivers, 250 m a.s.l. (0°28'00.0"S, 76°58'00.0"W) (Heinemann and Belkin 1979; GBIF-Secretariat 2025). (E).

**201. *Orthopodomyia phyllozoa* (Dyar & Knab, 1907)**.

**Synonym**. *Ma. phyllozoa* Dyar & Knab, 1907.

**Distribution**. Sto. Domingo de los Tsáchilas (formerly part of Pichincha): Reserva La Perla (00°01'03"S, 079°23'47"W) (GBIF-Secretariat 2025). INSPI collection—Sucumbíos: Nueva Loja (0°06'04.2"N, 76°52'47.8"W) ECU14118. (E, W).

### Tribe Sabethini (52 species)

**202. *Johnbelkinia longipes* (Fabricius, 1805)**.

**Synonym**. *Cx. longipes* Fabricius, 1805.

**Distribution**. Orellana (formerly Napo): Coca, confluence of Coca and Napo rivers, 250 m a.s.l. (0°28'00.0"S, 76°58'00.0"W); Orellana (formerly Napo), ~ 50 km E of El Coca, “Isla Pompeya” (island in Napo River at confluence with Jivino River) (0°26'12.0"S, 76°37'07.5"W) (Heinemann and Belkin 1979; Zavortink 1979; GBIF-Secretariat 2025). INSPI collection—Sucumbíos: Shushufindi (0°04'55.6"S, 76°38'37.0"W) ECU13365; Napo: Tena (1°01'50.0"S, 77°34'41.0"W) ECU7357. (E).

**203. *Johnbelkinia ulopus* (Dyar & Knab, 1906)**.

**Synonym**. *Lesticocampa culicivora* Dyar & Knab, 1907.

**Distribution**. Cañar: Cochancay, km 86 on Rt. 8 from Guayaquil (2°28'08.4"S, 79°17'50.6"W) (Heinemann and Belkin 1979; GBIF-Secretariat 2025); Manabí: Pacoche Lodge, “La Bomba” sector (1°04'04.6"S, 80°52'44.8"W); Los Ríos: Pichilingue (GBIF-Secretariat 2025); Bolívar: Balzapamba; Cañar: Cochancay, at km 86 on Rt. 8 (Zavortink 1979). INSPI collection—Sto. Domingo de los Tsáchilas: Santo Domingo (0°22'47.2"S, 79°16'41.8"W) ECU20402; Pichincha: Mindo (0°02'55.4"S, 78°46'30.7"W) ECU10267. (W).

204. *Limatus andinus* Levi-Castillo, 1954**†.

**Distribution**. Type locality: Los Ríos: Valencia (0°57'14.8"S, 79°21'10.3"W) (Stone et al. 1959); Ecuador (Levi-Castillo 1958; AFPMB 1998; Bánki et al. 2025). (W).

**205. *Limatus asulleptus* (Theobald, 1903)**.

**Synonyms**. *Li. methysticus* Dyar & Knab, 1909; *Dendromyia asulleptus* Theobald, 1903.

**Distribution**. Orellana (formerly Napo): Coca, confluence of Coca and Napo rivers, 250 m a.s.l. (0°28'00.0"S, 76°58'00.0"W), ~ 50 km E of El Coca, “Isla Pompeya” (island in Napo River at confluence with Jivino River) (00°26'12"S, 076°37'07.5"W); Los Ríos: Quevedo, 1 km E of Valencia (00°57'14.8"S, 079°21'10.3"W) (Heinemann and Belkin 1979; GBIF-Secretariat 2025). INSPI collection—Esmeraldas: San Lorenzo, San Francisco (1°17'18.0"N, 78°50'13.0"W) ECU3768; Imbabura: Lita (0°52'15.8"N, 78°27'06.6"W) ECU19850. (E, W).

**206. *Limatus durhamii* Theobald, 1901**.

**Synonyms**. *Dendromyia paraensis* Theobald, 1903; *Li. cacophrades* Dyar & Knab, 1909; *Li. exhibitor* Shannon & Ponte, 1927; *Simondella curvirostris* Laveran, 1902.

**Distribution**. Orellana (formerly Napo): Coca, confluence of Coca and Napo rivers, 250 m a.s.l. (0°28'00.0"S, 76°58'00.0"W); Orellana (formerly Napo), ~ 50 km E of El Coca, ‘‘Isla Pompeya’’ (island in Napo River at confluence with Jivino River) (00°26'12"S, 076°37'07.5"W); Los Ríos: Quevedo, 1 km E of Valencia, 200 m a.s.l.; Los Ríos: Quevedo, ~ 10 km SW of Pichilingue (01°06'11.9"S, 079°29'7.8"W); Los Ríos: Babahoyo, ~ 30 km E of Montalvo (01°49'11.9"S, 079°31'7.8"W); Sucumbíos (formerly Napo): Cuyabeno, ~ 50 km W of “Tarapoa, Campsite 5.5 km N”, 300 m a.s.l. (Heinemann and Belkin 1979; GBIF-Secretariat 2025); Orellana: Yasuní National Park, Tiputini Biodiversity Station, Lago Trail, 1,700 m a.s.l. (0°38'14.12"S, 76°9'43.74"W); Main Camp (0°38'13.52"S, 76°9'0.33"W); Lago Trail, 1,400 m a.s.l. (0°38'14.94"S, 76°9'34.46"W) (Linton et al. 2013); Imbabura: Lita (0°52'11.83"N, 78°27'05.56"W); Cachaco (0°49'41.19"N, 78°24'03.55"W). INSPI collection—Chimborazo, Cumandá ECU204; Morona Santiago: Yantzaza (3°48'46.6"S, 78°45'30.1"W) ECU6142; Sto. Domingo de los Tsáchilas: Santo Domingo (0°15'40.6"S, 79°11'45.4"W) ECU10224; Pichincha: Mindo (0°02'21.9"S, 78°46'07.2"W) ECU9999; Zamora Chinchipe: Zamora (4°05'15.7"S, 78°57'27.3"W) ECU10932; Esmeraldas: Borbón (11°05'22.0"N, 78°59'30.8"W) ECU28830. (E, W).

**207. *Limatus flavisetosus* de Oliveira Castro, 1935**.

**Distribution**. Orellana: Yasuní National Park, Tiputini Biodiversity Station (00°38'13.51"S, 76°09'0.31"W), Lago Trail, 2,000 m a.s.l. (0°38'15.54"S, 76°9'51.82"W); Lago Trail, 1,700 m a.s.l. (0°38'14.13"S, 76°9'43.83"W); Main Camp (0°38'13.52"S, 76°9'0.33"W); Harpía Trail, 750 m a.s.l. (00°37'50.2"S, 076°08'43.4"W); Maquisapa Trail, 6,200 m a.s.l. (00°37'54.9"S, 076°09'58.4"W) (Linton et al. 2013). INSPI collection—Sto. Domingo de los Tsáchilas: La Concordia (0°00'35.5"N, 79°22'60.0"W) ECU3288; Zamora Chinchipe: Copalinga (4°05'10.9"S, 78°57'25.2"W) ECU10938; Esmeraldas: Borbón ECU27567. (E, W).

208. *Limatus guayasi* Levi-Castillo, 1954 **†.

**Distribution**. Type locality: Guayas: El Empalme (Levi-Castillo 1954; Stone et al. 1959; GBIF-Secretariat 2025); Ecuador (Levi-Castillo 1958; AFPMB 1998; Bánki et al. 2025) (W).

**209. *Onirion imparis* Peyton & Harbach, 2000**.

**Distribution**. Orellana (formerly Napo): Coca, confluence of Coca and Napo rivers (Harbach and Peyton 2000); Orellana: Tiputini Biodiversity Station, Maquisapa Trail (00°37'54.9"S, 076°09'58.4"W), Tiputini River, 6 km from Tiputini Biodiversity Station (0°39'50.00"S, 76°12'56.01"W) (Linton et al. 2013). INSPI collection—Orellana: Loreto (0°31'08.0"S, 77°25'40.4"W) ECU10705. (E).

**210. Runchomyia (Ctenogoeldia) magna (Theobald, 1905)**.

**Synonym**. *Phoniomyia magnum* Theobald, 1905.

**Distribution**. Cañar: Cochancay, at km 86 on Rt. 8 from Guayaquil (02°28'08.4"S, 079°17'50.6"W); Los Ríos: Quevedo, ~ 30 km SW of Quevedo, 3 km S of El Empalme (01°06'12.0"S, 079°41'07.9"W) (Heinemann and Belkin 1979; GBIF-Secretariat 2025); Orellana: Yasuní National Park, Tiputini Biodiversity Station, near Tiputini River, ~ 8 km from Tiputini Biodiversity Station (0°39'53.00"S, 76°12'54.00"W) (Linton et al. 2013). (E, W).

**211. Sabethes (Peytonulus) identicus Dyar & Knab, 1907**.

**Synonym**. *Sa. lutzianus* Lane & Cerqueira, 1942.

**Distribution**. INSPI collection—Manabí: Manta (1°04'06.7"S, 80°53'13.3"W) ECU10638. Manabí: San Lorenzo (1°07'35.7"S, 80°50'41.3"W) ECU10619. (W).

**212. Sabethes (Peytonulus) luxodens Hall, Howard & Harbach, 1999†**.

**Distribution**. Type locality: Guayas: Guayaquil, 12 km W of Guayaquil on Rt. 3 (2°11'20.73"S, 79°59'1.73"W) (Hall et al. 1999); Orellana: Yasuní National Park, Tiputini Biodiversity Station, near Tiputini River, ~ 8 km from Tiputini Biodiversity Station (0°39'53.00"S, 76°12'54.00"W) (Linton et al. 2013). (E).

**213. Sabethes (Petyonulus) soperi Lane & Cerqueira, 1942**.

**Distribution**. Napo: National Reserve Colonso-Chalupas (0°56'09.8"S, 77°54'12.3"W) (Navarro et al. 2015). (E).

**214. Sabethes (Peytonulus) undosus (Coquillett, 1906)**.

**Synonym**. *Sa. undosus* Coquillett, 1906.

**Distribution**. Guayas: Guayaquil, at km 12 on Rt. 3, ~ 0.9 km W of checkpoint (02°11'32.6"S, 079°59'09.7"W) (Heinemann and Belkin 1979; GBIF-Secretariat 2025). (W).

**215. Sabethes (Sabethes) albiprivus Theobald, 1903**.

**Synonyms**. *Sa. albiprivatus* Lutz, 1904; *Sa. albiprivatus* Theobald, 1907; *Sa. neivai* Petrocchi, 1927.

**Distribution**. INSPI collection—Chimborazo: Cumandá (2°12'18.09"S, 79°8'9.56"W) ECU6283. Esmeraldas: Rioverde (1°2'31.13"N, 79°22'59.48"W) ECU7244, ECU8059; Guayas: Progreso (2°24'57.86"S. 80°21'57.32"W) ECU7154; Los Ríos: Quevedo (01°1'4.83"S, 079°27'32.71"W) ECU6094; Manabí: Chone, San Andrés (0°41'17.7"S, 80°05'48.4"W) ECU6121; Manabí: Cantón Junín ECU6441; Sto. Domingo de los Tsáchilas: Sueño de Bolívar (0°13'16.00"S, 79°9'54.18"W) ECU10100. (NR, W).

**216. Sabethes (Sabethes) amazonicus Gordon & Evans, 1922**.

**Synonyms**. *Sa. kappleri* Bonne, 1923; *Sa. longfieldae* Edwards, 1928.

**Distribution**. INSPI collection—Zamora Chinchipe: Zamora, Copalinga (4°05'14.4"S, 78°57'22.8"W) ECU10694. (E).

**217. Sabethes (Sabethes) belisarioi Neiva, 1908**.

**Synonyms**. *Sa. argyronotum* Edwards, 1928; *Sa. goeldii* Howard, Dyar & Knab, 1917; *Sa. schausi* Dyar & Knab, 1908; *Sebethes bellisarioi* Neiva, 1908.

**Distribution**. INSPI collection—Zamora Chinchipe: Copalinga (4°05'13.2"S, 78°57'24.6"W) ECU10718; Sucumbíos: Shushufindi (0°11'01.5"S, 76°39'22.6"W) ECU16528. Sucumbíos: Pacayacu (0°02'09.9"S, 76°35'41.8"W) ECU13405. (NR, E).

**218. Sabethes (Sabethes) bipartipes Dyar & Knab, 1906**.

**Synonym**. *Sa. chroiopus* Dyar & Knab, 1913.

**Distribution**. Los Ríos: Quevedo, 1 km E of Valencia (00°56"46.9"S, 079°20'10.5"W); Los Ríos: Quevedo, ~ 10 km SW of Pichilingue; Guayas: Guayaquil, 14 km W of Guayaquil on Rt. 3 (2°10'48.21"S, 80°0'6.49"W), 12 km W of Guayaquil on Rt. 3 (2°11'20.73"S, 79°59'1.73"W) (Heinemann and Belkin 1979; GBIF-Secretariat 2025). (W).

**219. Sabethes (Sabethes) cyaneus (Fabricius, 1805)**.

**Synonyms**. *Cx. cyaneus* Fabricius, 1805; *Cx. remipes* Wiedemann, 1828; *Sa. locuples* Robineau-Desvoidy, 1827.

**Distribution**. INSPI collection—Sucumbíos: Nueva Loja (0°09'53.7"N, 76°40'43.0"W) ECU18477; Esmeraldas: Alto Tambo (0°58'33.3"N, 78°34'28.2"W) ECU17956. (E, W).

**220. Sabethes (Sabethes) purpureus (Theobald, 1901)**.

**Synonyms**. *Sa. purpureus* Peryassú, 1908; *Sa. remipusculus* Dyar, 1924; *Sabethoides purpureus* Theobald, 1907.

**Distribution**. INSPI collection—Esmeraldas: Rioverde, road to El Alto (1°3'2.31"N, 79°25'13.67"W) ECU8528; Sucumbíos: Cuyabeno (0°13'3.41"S, 75°48'24.87"W) ECU10720. (NR, E, W).

**221. Sabethes (Sabethes) quasicyaneus Peryassú, 1922**.

**Distribution**. INSPI collection—Esmeraldas: San Lorenzo (1°17'18.00"N, 78°50'13.00"W) ECU3767; Esmeraldas: road to El Alto (1°2'16.30"N, 79°14'55.54"W) ECU8531; Guayas: Progreso (2°24'57.86"S, 80°21'57.32"W) ECU7158; Manabí: Junín (0°55'31.4"S, 80°12'29.7"W) ECU20172. (NR, W).

**222. Sabethes (Sabethes) intermedius (Lutz, 1904)**.

**Synonym**. *Sbn. intermedius* Lutz, 1904.

**Distribution**. Napo: Biological Reserve Colonso-Chalupas (Navarro et al. 2015). (E).

**223. Sabethes (Sabethoides) chloropterus (von Humboldt, 1819) †**.

**Synonyms**. *Cx. chloropterus* Humboldt, 1819; *Sa. confusus* Theobald, 1901; *Sa. imperfectus* Bonne-Wepster & Bonne, 1920; *Sa. rangeli* Surcouf & Gonzales-Rincones, 1911.

**Distribution**. Type locality Guayas: Guayaquil River, near “Borodon’’ most likely refers to Samborondón (Stone et al. 1959; Belkin 1968); Los Ríos: Babahoyo, ~ 30 km E of Montalvo, “Hacienda Mora’’ (01°45'49.2"S, 079°14'12"W) (Heinemann and Belkin 1979; GBIF-Secretariat 2025). INSPI collection—Sucumbíos: Cuyabeno (0°12'56.3"S, 75°48'33.6"W) ECU10717. (E, W).

**224. Sabethes (Sabethoides) glaucodaemon (Dyar & Shannon, 1925)**.

**Synonym**. *Sbo. glaucodaemon* Dyar & Shannon, 1925.

**Distribution**. INSPI collection—Sucumbíos: Cuyabeno (0°13'4.53"S, 75°48'33.55"W) ECU10716; Sucumbíos: Shushufindi, La Invasión (0°11'57.03"S, 76°37'31.83"W) ECU7093. (NR, E).

**225. *Trichoprosopon andinum* Levi-Castillo, 1953†**.

**Distribution**. Type locality: Cotopaxi: Macuchi, 1,500 m a.s.l. (00°59'60"S, 079°04'00"W) (Levi-Castillo 1953b; Stone et al. 1959). (A).

**226. *Trichoprosopon compressum* Lutz, 1905**.

**Synonym**. *Tr. compressum* var. *Mogilasium* from Panamá. *Tr. compressum mogilasium* (Dyar & Knab, 1907).

**Distribution**. Los Ríos: Quevedo, 4 km W of Valencia (1°01'00.0"S, 79°21'00.0"W); Esmeraldas: Alto Tambo (0°55'49.00"N, 78°32'37.00"W) (Navarro et al. 2015); Orellana: Coca, Confluence of Coca and Napo rivers; Coca, ~ 50 km E of “Isla Pompeya” (island in Napo River at confluence with Jivino River); Manabí: Pacoche Lodge (1°03'59.00"S, 80°52'51.2"W) (GBIF Secretariat 2025). INSPI collection—Morona Santiago: Parroquia Macuma. Research Station Wisui (2°05'13.0"S, 77°45'03.4"W) ECU3516; Sucumbíos: Shushufindi (0°11'58.8"S, 76°35'22.0"W) ECU20474. (E, W).

227. *Trichoprosopon cotopaxense* Levi-Castillo, 1953**†.

**Note**. Described as cotopaxensis. Changed to cotopaxense (Stone 1967). Nomen dubium, possible synonyms *Tr. digitatum* and *Johnbelkinia ulopus* (Zavortink 1979). Provisionally accepted name (Bánki et al. 2025). Subgenus nomina dubia (Zavortink 1979).

**Distribution**. Type locality: Cotopaxi: Macuchi (00°59'10.2"S, 079°04'00"W) (Levi-Castillo 1953b; Stone et al. 1959). Ecuador (Levi-Castillo 1958). (A).

**228. *Trichoprosopon evansae* Antunes, 1942**.

**Distribution**. Napo, Orellana, and Pastaza (formerly Napo-Pastaza) (Levi-Castillo 1953c). Ecuador (Levi-Castillo 1958). (E).

**229. *Trichoprosopon digitatum* (Rondani, 1848)**.

**Synonyms**. *Cx. digitatus* Rondani, 1848; *Joblotia subsplendens* Martini, 1931; *Tr. nivipes* Theobald, 1901; *Tr. splendens* Lutz, 1904; *Tr. wilsoni* Ludlow, 1918.

**Distribution**. Sto. Domingo de los Tsáchilas (formerly Pichincha): Valle Hermoso; Orellana (formerly Napo), ~ 50 km E of El Coca, ‘‘Isla Pompeya” (island in Napo River at confluence with Jivino River) (00°26'12"S, 076°37'07.5"W); (GBIF-Secretariat 2025); Napo: Coca, confluence of Coca and Napo rivers, 250 m a.s.l.; Los Ríos: Quevedo, 1 km E of Valencia, 200 m a.s.l. (01°00'00"S, 076°00'00"W); Los Ríos: Quevedo, 4 km W of Valencia, 200 m a.s.l. (1°01'00.0"S, 79°21'00.0"W); Los Ríos: Quevedo, ~ 30 km SW of Quevedo, 3 km S of El Empalme, 100 m a.s.l.; Los Ríos: Babahoyo, ~ 30 km E of Montalvo, 100 m a.s.l. (1°48'00.0"S, 79°20'00.0"W); Los Ríos: Babahoyo, ~ 30 km E of Montalvo, “Hacienda Mora”, 100 m a.s.l. across river at end of main path of Montalvo (01°49'11.9"S, 079°31'07.8"W); Sucumbíos (formerly Napo): Cuyabeno, ~ 50 km W of ‘‘Tarapoa, Campsite 5.5 km N” (0°10'00.0"S, 76°20'00.0"W) (Heinemann and Belkin 1979); Orellana: Yasuní National Park, Tiputini Biodiversity Station, Lago Trail, 2,200 m a.s.l. (0°38'9.80"S, 76°9'51"W); Lago Trail, 2,000 m a.s.l. (0°38'15.54"S, 76°9'51.82"W); Harpía Trail, 800 m a.s.l. (0°37'48.64"S, 76°8'43.81"W); Tower (0°39'52.99"S, 76°12'54.00"W); Numa Trail (0°38'15.50"S, 76°8'59.31"W); Main Camp (0°38'13.52"S, 76°9'0.33"W) (00°38'17"S, 076°09'22.2"W); Main Camp at Chorongo Trail (0°38'10.87"S, 76°8'55.40"W); Lago Trail 975 m (0°38'17.01"S, 76°9'22.21"W); Lago Trail 1400 m (0°38'14.94"S, 76°9'34.46"W); near Main Camp (022'15.14"S, 76°9'0.32"W) (Linton et al. 2013). INSPI collection—Morona Santiago: Macuma, Research Station Wisui (2°05'13.0"S, 77°45'03.4"W) ECU3517; Sucumbíos: Shushufindi (0°10'19.3"S, 76°38'08.0"W) ECU14842; Chimborazo: Cumandá (2°12'18.1"S, 79°08'10.0"W) ECU20208. (E, W).

**230. *Trichoprosopon lampropus* (Howard, Dyar & Knab, 1913)**.

**Synonym**. *Lesticocampa lampropus* Howard, Dyar & Knab, 1913.

**Distribution**. Orellana: Yasuní National Park, Tiputini Biodiversity Station, Lago Trail, 975 m a.s.l. (0°38'17.01"S, 76°9'22.21"W) (Linton et al. 2013). (E).

**231. *Trichoprosopon lanei* (Antunes, 1937)**.

**Synonym**. *Goeldia lanei* Antunes, 1937.

**Distribution**. Orellana (formerly Napo): ~ 50 km E of El Coca, ‘‘Isla Pompeya’’ (island in Napo River at confluence with Jivino River) (00°26'12"S, 076°37'07.5"W); Orellana (formerly Napo): Coca, confluence of Coca and Napo rivers, 250 m a.s.l. (0°28'00.0"S, 76°58'00.0"W) (Heinemann and Belkin 1979; GBIF-Secretariat 2025). (E).

**232. *Trichoprosopon pallidiventer* (Lutz, 1905)**.

**Synonym**. *Hyloconops pallidiventer* Lutz, 1905.

**Distribution**. Orellana (formerly Napo), Coca, ~ 50 km E of ‘‘Isla Pompeya’’ (island in Napo River at confluence with Jivino River) (0°26'00.0"S, 76°37'00.0"W) (Heinemann and Belkin 1979). INSPI collection—Imbabura: Cachaco; Esmeraldas: San Francisco de Bogotá; Pichincha: Nanegalito (0°04'00.0"N, 78°39'60.0"W) ECU3257. (E, W).

**233. *Trichoprosopon vonplesseni* (Dyar & Knab, 1906) †**.

**Synonym**. *Lesticocampa vonplesseni* Dyar & Knab, 1906.

**Distribution**. Type locality: Upper Pastaza River (Napo-Pastaza) (Lane 1953; Belkin et al. 1965a; Heinemann and Belkin 1979). INSPI collection—Sucumbíos: Shushufindi (0°11'03.1"S, 76°37'59.5"W) ECU14745; Morona Santiago: Río Blanco (2°20'17.3"S, 78°09'46.6"W) ECU11583. (E).

**234. Wyeomyia (Decamyia) pseudopecten Dyar & Knab, 1906**.

**Synonyms**. *Den. bicompressa* Lutz, 1928; *Wy. cara* Dyar & Knab, 1909; *Wy. eloisa* Howard, Dyar & Knab, 1912; *Wy. galoa* Dyar & Knab, 1906.

**Distribution**. Orellana (formerly Napo): Coca, confluence of Coca and Napo rivers (0°28'00.0"S, 76°58'00.0"W); ~ 50 km of ‘‘Isla Pompeya” (island in Napo River at confluence with Jivino River) (0°26'00.0"S, 76°3700.0"W) (Heinemann and Belkin 1979; GBIF-Secretariat 2025); Orellana: Yasuní National Park, Tiputini Biodiversity Station, Main Camp (0°38'13.52'S 76°9'0.33"W) (0°38'8.99"S, 76°9'34.96"W) (Linton et al. 2013). (E).

**235. Wyeomyia (Decamyia) ulocoma (Theobald, 1903)**.

**Synonyms**. *Den. ulocoma* Theobald, 1903; *Wy. cacodela* Dyar & Knab, 1909; *Wy. oindus* Dyar & Knab, 1909; *Wy. pantoia* Dyar & Knab, 1909.

**Distribution**. Orellana: Yasuní National Park, Tiputini Biodiversity Station, Main Camp (0°38'13.52"S, 76°9'0.33"W); Matapalo Trail, 450 m a.s.l. (0°38'15.11"S, 76°8'50.43"W) (Linton et al. 2013). (E).

**236. Wyeomyia (Dendromyia) complosa (Dyar, 1928)**.

**Synonym**. *Den. complosa* Dyar, 1928.

**Distribution**. Los Ríos: Quevedo, Valencia, 1 km E of Valencia (00°56'47"S, 079°20'10.5"W); Los Ríos: Quevedo, 1 km E of Valencia (01°01'S 79°21'W), Quevedo, ~ 10 km SW of Pichilingue (1°0600.0"S, 79°29'00.0"W); Cañar: Cochancay, 86 km E of Guayaquil on Rt. 8 (02°28'08.4"S, 079°17'50.6"W) (Heinemann and Belkin 1979; GBIF-Secretariat 2025); Pichincha: San Miguel de los Bancos (0°01'25.37"N, 78°53'42.74"W) (Navarro et al. 2013b). (W).

**237. Wyeomyia (Dodecamyia) aphobema Dyar, 1918**.

**Synonyms**. *Wy. aequatorialis* Levi-Castillo, 1952; *Wy. bodkini* Edwards, 1922.

**Distribution**. Orellana (formerly Napo): Coca, confluence of Coca and Napo rivers, 250 m a.s.l. (0°28'00.0"S, 76°58'00.0"W) (Heinemann and Belkin 1979); Orellana: Yasuní National Park, Tiputini Biodiversity Station, Main Camp (0°38'13.52"S, 76°9'0.33"W); Guacamayo Trail, 2,300 m a.s.l. (0°38'2.42"S, 76°9'49.87"W); Chorongo Trail, 375 m a.s.l. (Linton et al. 2013). (E).

238. Wyeomyia (Hystatomyia) esmeraldasi (Levi-Castillo, 1955) **†. Described as *Phoniomyia esmeraldasi* by Levi-Castillo (1955).

**Distribution**. Type locality: Esmeraldas: Island of Changuaral, Sardinas Bight (01°23'57.2"N, 078°52'34.3"W) (Levi-Castillo 1955b; Stone et al. 1959); Ecuador (Levi-Castillo 1958; AFPMB 1998; Bánki et al. 2025). (W).

**239. Wyeomyia (Hystatomyia) lamellata (Bonne-Wepster & Bonne, 1919)**.

**Synonym**. *Histatomyia lamellata* Bonne-Wepster & Bonne, 1919.

**Distribution**. Orellana: Yasuní National Park, Tiputini Biodiversity Station, Main Camp (00°38'17"S, 076°08'42"W) (Linton et al. 2013). (E).

**240. Wyeomyia (Miamyia) codiocampa Dyar & Knab, 1907**.

**Distribution**. Orellana: Yasuní National Park, Tiputini Biodiversity Station, near Tiputini River, ~ 8 km from Tiputini Biodiversity Station (0°39'53.00"S, 76°12'54.00"W); Maquisapa Trail, 6,200 m a.s.l. (00°37'54.9"S, 076°09'58.4"W) (Linton et al. 2013). (E).

**241. Wyeomyia (Miamyia) oblita (Lutz, 1904)**.

**Synonyms**. *Dendromyia oblita* Lutz, 1904; *Miamyia pintoi* Lima, 1930; *Wy. fallax* Bonne-Wepster & Bonne, 1919.

**Distribution**. Orellana: Yasuní National Park, Tiputini Biodiversity Station, near Tiputini River, ~ 8 km from Tiputini Biodiversity Station (0°39'53.00"S, 76°12'54.00"W); Maquisapa Trail, 6,200 m a.s.l. (00°37'54.9"S, 076°09'58.4"W) (Linton et al. 2013); Orellana: Yasuní National Park, at river tip ~ 8 km from Tiputini (00°39'53"S, 076°12'54"W) (GBIF-Secretariat 2025). (E).

**242. Wyeomyia (Nunezia) bicornis (Root, 1928)**.

**Synonym**. *Dendromyia bicornis* Root, 1928.

**Distribution**. Imbabura: Lita (0°52'11.8"N, 78°27'05.6"W) (Navarro et al. 2015) (W).

**243. Wyeomyia (Phoniomyia) lassalli (Bonne-Wepster & Bonne, 1921)**.

**Synonym**. *Dyarina lassalli* Bonne-Wepster & Bonne, 1921.

**Distribution**. Napo, Orellana, and Pastaza (formerly Napo-Pastaza) (Levi-Castillo 1953c). Ecuador (Levi-Castillo 1958). (E).

**244. Wyeomyia (Phoniomyia) splendida Bonne-Wepster & Bonne, 1919**.

**Distribution**. Manabí; Esmeraldas; Napo, Orellana, and Pastaza (formerly Napo-Pastaza) (Levi-Castillo 1953c); Ecuador (Levi-Castillo 1958). (E, W).

**245. *Wyeomyia albosquamata* Bonne-Wepster & Bonne, 1919**.

**Distribution**. Orellana: Yasuní National Park, Tiputini Biodiversity Station, Main Camp (00°38'17"S, 076°08'42"W); Guacamayo Trail, 2300 m a.s.l. (0°38'2.42"S, 76°9'49.87"W) (Linton et al. 2013). (W).

246. *Wyeomyia amazonica* Levi-Castillo, 1954**†. Uncertain subgenus.

**Distribution**. Type locality Napo: Tena (00°59'48"S, 077°48'49.2"W); Pastaza (Levi-Castillo 1954; Stone et al. 1959); Ecuador (Levi-Castillo 1958; Bánki et al. 2025). (E).

247. *Wyeomyia aphobema* var. aequatorialis Levi-Castillo, 1952 **†. Uncertain subgenus.

**Distribution**. Type locality: Los Ríos: “Hacienda Pichilingue” (01°06'11.9"S, 079°29'7.8"W) (Levi-Castillo 1952b, 1953f; Stone et al. 1959); Ecuador (Levi-Castillo 1958; AFPMB 1998; Bánki et al. 2025). (W).

**248. *Wyeomyia chalcocephala* Dyar & Knab, 1906**.

**Synonym**. *Wy. luciae* Senevet & Chabelard, 1942. Uncertain subgenus.

**Distribution**. Los Ríos: Quevedo, 1 km E of Valencia; Guayas: Cochancay, at km 86 on Rt. 8 from Guayaquil (02°28'08.4"S, 079°17'50.6"W); Guayas: Guayaquil, 12 km W of Guayaquil on Rt 3, ~ 0.9 km W of checkpoint at km 12 on Rt. 3 (02°11'32.6"S, 079°59'09.7"W); Cañar: Cochancay, at km 86 on Rt. 8 from Guayaquil (Heinemann and Belkin 1979; GBIF-Secretariat 2025). (W).

**249. *Wyeomyia melanocephala* Dyar & Knab, 1906**.

**Synonyms**. *Dendromyia melanoides* Root, 1928; *Den. typharum* Shannon & Ponte, 1927; *Prosopolepis hemisiris* Dyar & Shannon, 1925; *Sabethes canfieldi* Dyar & Knab, 1907; *Wy. agnostips* Dyar & Knab, 1907; *Wy. fauna* Dyar & Knab, 1919; *Wy. grenadensis* Edwards, 1916; *Wy. modalma* Dyar, 1922; *Wy. pandora* Dyar & Knab, 1909. Uncertain subgenus.

**Distribution**. Orellana (formerly Napo): Coca, confluence of Coca and Napo rivers, 250 m a.s.l. (0°28'00.0"S, 76°58'00.0"W); Orellana (formerly Napo): ~ 50 km E of El Coca, “Isla Pompeya” (island in Napo River at confluence with Jivino River) (00°26'12"S, 076°37'07.5"W) (Heinemann and Belkin 1979; GBIF-Secretariat 2025). (E).

**250. Wyeomyia (Wyeomyia) arthrostigma (Lutz, 1905)**.

**Synonyms**. *Dendromyia arthrostigma* Lutz, 1905; *Wy. bromeliarum* Dyar & Knab, 1906; *Wy. drapetes* Dyar & Knab, 1909; *Wy. espartana* Dyar & Knab, 1906; *Wy. panamena* Dyar & Knab, 1907.

**Distribution**. Orellana (formerly Napo): ~ 50 km E of El Coca, ‘‘Isla Pompeya’’ (island in Napo River at confluence with Jivino River) (00°26'12"S, 076°37'07.5"W) (Heinemann and Belkin 1979; GBIF-Secretariat 2025). (E).

**251. Wyeomyia (Wyeomyia) celaenocephala Dyar & Knab, 1906**.

**Synonyms**. *Phoniomyia chrysomus* Dyar & Knab, 1907; *Pho. philophone* Dyar & Knab, 1907; *Wy. mataea* Dyar & Knab, 1908; *Wy. megalodora* Dyar & Knab, 1908.

**Distribution**. Carchi: El Baboso (0°53'47.05"N, 78°26'34.98"W) (Navarro et al. 2015). (W).

**252. Wyeomyia (Wyeomyia) medioalbipes Lutz, 1904**.

**Synonyms**. *Wy. telestica* Dyar & Knab, 1906; *Wy. abascanta* Dyar & Knab, 1908.

**Distribution**. Orellana: Yasuní National Park, Tiputini Biodiversity Station, Tower (0°38'17.0"S, 76°08'42.0"W) (Linton et al. 2013). (E).

**253. Wyeomyia (Wyeomyia) scotinomus (Dyar & Knab, 1907)**.

**Synonyms**. *Phoniomyia scotinomus* Dyar & Knab, 1907; *Wy. abrachys* Dyar & Knab, 1909; *Wy. camptocomma* Dyar, 1924; *Wy. chresta* Dyar & Knab, 1909; *Wy. hapla* Dyar & Knab, 1909; *Wy. homothe* Dyar & Knab, 1907; *Wy. incana* Dyar, 1922; *Wy. leucopisthepus* Dyar & Knab, 1907.

**Distribution**. Manabí; Esmeraldas; Los Ríos; Napo, Orellana, and Pastaza (formerly Napo-Pastaza) (Levi-Castillo 1953c); Ecuador (Levi-Castillo 1958). (E, W).

### Tribe Toxorhynchitini (7 species)

**254. *Toxorhynchites aequatorianus* Levi-Castillo, 1953. Nomen dubium (Stone et al. 1959)**.

**Distribution**. Los Ríos: Pichilingue (01°06'11.9"S, 079°29'7.8"W) (Levi-Castillo 1953e; Bánki et al. 2025). (W).

**255. *Toxorhynchites haemorrhoidalis superbus* (Fabricius, 1787)**.

**Synonym**. *Megarhinus superbus* Dyar & Knab, 1906.

**Distribution**. Guayas: Guayaquil and its surroundings (Campos 1925); Guayas; Manabí; Los Ríos (Levi-Castillo 1953c). (W).

**256. Toxorhynchites (Lynchiella) bambusicola Knight & Rozeboom, 1946**.

**Synonyms**. *Megarhinus aldrichanus* Bonne-Wepster & Bonne, 1919; *Megarhinus bambusicola* Lutz & Neiva, 1913.

**Distribution**. Orellana: Yasuní National Park, Tiputini Biodiversity Station, Maquisapa Trail, 6,200 m a.s.l. (00°37'54.9"S, 076°09'58.4"W); Tiputini River, 6 km from Tiputini Biodiversity Station (0°39'50.00"S, 76°12'56.01"W) (Linton et al. 2013). (E).

**257. Toxorhynchites (Lynchiella) guadeloupensis (Dyar & Knab, 1906)**.

**Synonyms**. *Megarrhina horei* Gordon & Evans, 1922; *Meg. tucumanus* Brèthes, 1926; *Meg. arborealis* Shannon & Ponte, 1927; *Meg. guianensis* Bonne-Wepster & Bonne, 1953.

**Distribution**. INSPI collection—Manabí: La Pila (1°6'43.28"S, 80°35'18.33"W) ECU11017. (NR, W).

**258. Toxorhynchites (Lynchiella) haemorrhoidalis (Fabricius, 1787)**.

**Synonym**. *Cx. haemorrhoidalis* Fabricius, 1787.

**Distribution**. Orellana (formerly Napo): Coca, ~ 50 km E of ‘‘Isla Pompeya” (island in Napo River at confluence with Jivino River) (0°26'00"S, 76°36'60"W); ~ 50 km W of ‘‘Tarapoa (J-C Camp)”; 200 m south of the airstrip” (0°10'00"S, 76°20'00"W) (Heinemann and Belkin 1979; GBIF-Secretariat 2025); Orellana: Yasuní National Park, Tiputini Biodiversity Station, Chorongo Trail, 800 m a.s.l. (0°37'59.04"S, 76°9'2.02"W); Maquisapa Trail, 7,200 m a.s.l. (0°37'54.89"S, 76°9'58.37"W) (Linton et al. 2013). INSPI collection—Sucumbíos: Nueva Loja (0°03'49.3"N, 76°59'41.8"W) ECU16346; Sucumbíos: Shushufindi (0°11'25.8"S, 76°37'39.1"W) ECU14789. (E).

**259. Toxorhynchites (Lynchiella) hypoptes Knab, 1907**.

**Distribution**. Guayas: Guayaquil, 12 km W of Guayaquil on Rt. 3. (2°11'20.73"S, 79°59'1.73"W); Manabí: Portoviejo (1°03'00.0"S, 80°27'00.0"W) (Heinemann and Belkin 1979). (W).

**260. Toxorhynchites (Lynchiella) theobaldi (Dyar & Knab, 1906)**.

**Synonyms**. *Cx. ferox* Wiedemann, 1828; *Megarhinus ambiguus* Dyar & Knab, 1906; *Megarhinus moctezuma* Dyar & Knab, 1906; *Megarhinus theobaldi* Dyar & Knab, 1906; *Megarhinus trinidadensis* Dyar & Knab, 1906; *Megarhinus wiedemanni* Dyar & Knab, 1906.

**Distribution**. Manabí: Portoviejo (01°03'01.4"S, 080°27'01.5"W); Guayaquil; 12 km W of Guayaquil on Rt. 3, ~ 0.9 km W of checkpoint at km 12 on Rt. 3 (GBIF-Secretariat 2025). INSPI collection—Esmeraldas: Tonsupa (0°53'10.0"N, 79°48'26.9"W) ECU11472. (W).

### Tribe Uranotaeniini (6 species)

**261. Uranotaenia (Uranotaenia) calosomata Dyar & Knab, 1907**.

**Synonym**. *Ur. albitarsis* Gordon & Evans, 1922.

**Distribution**. Orellana (formerly Napo): Coca, confluence of Coca and Napo rivers, 250 m a.s.l. (0°28'00.0"S, 76°58'00.0"W) (Heinemann and Belkin 1979; GBIF-Secretariat 2025); Orellana: Yasuní National Park, Tiputini Biodiversity Station, Lake ~4 hectares (0°38'13.52"S, 76°9'0.33"W) (Linton et al. 2013). INSPI collection—Loja: Chaguarpamba (3°52'45.1"S, 79°37'35.4"W) ECU4385. (E).

**262. Uranotaenia (Uranotaenia) ditaenionota Prado, 1931**.

**Synonym**. *Ur. burkii* Lane, 1936.

**Distribution**. INSPI collection—El Oro: Marcabelí, Aguas Negras (3°47'6.10"S, 79°54'44.37"W) ECU4911. (NR, W).

263. Uranotaenia (Uranotaenia) aequatorianna Levi-Castillo, 1953**†.

**Distribution**. Type locality: Los Ríos (Levi-Castillo 1953c); Los Ríos: Babahoyo (01°48'55.5"S, 079°31'00"W) (GBIF-Secretariat 2025); Los Ríos: “Hacienda Pichilingue” (Stone et al. 1959); Ecuador (Levi-Castillo 1958; AFPMB 1998; Bánki et al. 2025). (W).

**264. Uranotaenia (Uranotaenia) geometrica Theobald, 1901**.

**Distribution**. Guayas: Guayaquil and its surroundings (Campos 1925); Orellana (formerly Napo), ~ 50 km E of El Coca, ‘‘Isla Pompeya” (island in Napo River at confluence with Jivino River) (00°26'12"S, 076°37'07.5"W) (Heinemann and Belkin 1979); Sto. Domingo de los Tsáchilas (formerly part of Pichincha): Valle Hermoso, near Hostería “Valle Hermoso” (00°05'06"S, 079°16'35"W); Orellana: Yasuní National Park, Tiputini Biodiversity Station (00°38'13.5"S, 076°09'0.3"W) (GBIF-Secretariat 2025). (E, W).

**265. Uranotaenia (Uranotaenia) leucoptera (Theobald, 1907)**.

**Synonym**. *Anisocheleomyia leucoptera* Theobald, 1907.

**Distribution**. INSPI collection—Sucumbíos: Nueva Loja (0°06'03.0"N, 76°52'50.2"W) ECU13915. (E).

**266. Uranotaenia (Uranotaenia) lowii Theobald, 1901**.

**Synonyms**. *Ur. continentalis* Dyar & Knab, 1906; *Ur. minuta* Theobald, 1907; *Ur. monilis* Shannon & Ponte, 1927.

**Distribution**. Los Ríos: Babahoyo, 5 km W of Babahoyo, 50 m a.s.l.; Guayas: Chongón, 24 km W of Guayaquil on Rt. 3, near road to Chongón (02°13'10.5"S, 080°05'05.6"W); Guayas: Guayaquil, 10 km W of Guayaquil on Rt. 3 (02°11'32.6"S, 079°58'05"W); Esmeraldas: near Atacames (00°52'40"N, 079°49'47"W) (Heinemann and Belkin 1979; GBIF-Secretariat 2025). INSPI collection—El Oro: Huaquillas (3°29'07.4"S, 80°13'30.0"W) ECU20195. (W).

## Discussion

The study of mosquitoes in Ecuador dates to 1819, when Alexander von Humboldt provided the first descriptions of local species (Belkin et al. 1965b). Later, Francisco Campos expanded the research on Ecuadorian mosquito fauna, publishing several studies on the subject. Campos primarily collected specimens from Guayaquil and coastal regions (Campos 1921, 1922, 1924, 1925). These specimens were sent to the entomologist Harrison G. Dyar, who described numerous new species from Ecuador between 1918 and 1925, some of which were named in honor of Campos (Levi-Castillo 1952a). Subsequently, Levi-Castillo made important contributions to the understanding to the country’s mosquito fauna. By the 1960s, 28 Culicidae species were documented in Ecuador (Belkin et al. 1965b), of which 19 species were described by Levi-Castillo. However, most of the specimens, apparently, went missing (Zavortink 1979), and some of the material is now at the United States National Museum (USNM) (Bram 1967).

The number of species recorded in Ecuador is significant considering the country’s size. By comparison, Brazil has 447 mosquito species, Mexico has approximately 244 (Ortega-Morales et al. 2023), Colombia 251 species (World Population Review 2025), and Perú 181 species (Ayala-Sulca et al. 2021). Foley et al. (2007) reported only 118 species, 23 endemic species, and 34 types for Ecuador. However, the most recent comprehensive Culicidae listing for the country identifies 179 species (Linton et al. 2013). In this study, we add 87 species, including 17 new national records, bringing the checklist of mosquitoes of Ecuador to 266 species. These new national records correspond to species within genera and subgenera already known in the country, so no new higher-level taxa are reported. Molecular barcoding resolved 90 specimens that were difficult to identify morphologically. One specimen (*Aedes albopictus*) was readily identified by taxonomic keys, but molecular confirmation was conducted as a control. The mosquito species documented in this revision account for 7% of the global Culicidae diversity, highlighting Ecuador’s substantial contribution to the overall mosquito biodiversity.

Despite this diversity, mosquito taxonomy in Ecuador remains an understudied field, with limited systematic research on the country’s mosquito diversity. To address this gap, we integrate molecular identification techniques as a complement to traditional morphology-based taxonomy. This integrative approach represents an essential step toward resolving the country’s understudied mosquito taxonomy and strengthening the baseline knowledge required for vector surveillance and more effective vector-borne disease control.

### Species of medical importance

Species of medical importance are summarized in Suppl. material 2, which provides a comprehensive classification of mosquito species according to the type of vectorial evidence (confirmed vectors, natural infections, experimental infections, and pathogen detection only).

#### Subfamily Anophelinae Grassi, 1900.

There are 472 formally recognized species of *Anopheles* mosquitoes (Harbach 2013, 2025), and only a small fraction act as vectors for human diseases. In Ecuador, 42 species of this genus have been recorded. *Anopheles* mosquitoes are the primary vectors for the *Plasmodium* parasite, responsible for malaria, as well as for the O’nyong-nyong virus. Some species transmit filarial nematodes, including *Wuchereria bancrofti*, *Brugia malayi*, and *Brugia timori*. *Anopheles* mosquitoes are not typically recognized as arbovirus vectors; however, certain anthropophilic species that frequently feed on vertebrates may become exposed to circulating viruses, potentially acquiring and transmitting arboviruses (Hernandez-Valencia et al. 2023).

The species that have been reported as infected with the malaria parasites in the Americas are: An. (Ano.) fluminensis (Neves et al. 2013), An. (Ano.) mattogrossensis (Pimenta et al. 2015), An. (Ano.) neomaculipalpus (Moreno et al. 2005), An. (Ano.) pseudopunctipennis (Valderrama et al. 2021; Naranjo-Díaz and Correa 2025), An. (Ano.) vestitipennis (Loyola et al. 1991), *An. (Ker.) cruzii*, *An. (Ker.) homunculus* (Lorenz et al. 2012), An. (Ker.) pholidotus (Escovar et al. 2014, Naranjo-Díaz and Correa 2025), An. (Ker.) neivai, An. (Ano.) punctimacula, An. (Ano.) calderoni, An. (Nys.) albimanus, An. (Nys.) nuneztovari (Naranjo-Díaz and Correa 2025), An. (Nys.) aquasalis (Alencar et al. 2023), An. (Nys.) oswaldoi, An. (Nys.) triannulatus (Pimenta et al. 2015), An. (Nys.) rangeli (Quiñones et al. 2006), An. (Nys.) trinkae (Hayes et al. 1987), An. (Nys.) benarrochi (Pereira-Silva et al. 2022), An. (Nys.) darlingi (Naranjo-Díaz and Correa 2025, Pontual et al. 2025), An. (Nys.) marajoara (Brochero et al. 2010).

#### Subfamily Culicinae Meigen, 1818.

Aedeomyia (Aedomyia) squamipennis is the only species of this genus in Ecuador, with records exclusively from the western lowlands of the Andes (Levi-Castillo 1953c; Ramírez-Aguilar 1953). This species has been identified as an important vector of Gamboa virus (GAMV) and avian malaria (Burkett-Cadena and Blosser 2017), and there is evidence suggesting its role in the transmission of Venezuelan equine encephalitis virus (VEEV) (Martin et al. 2024). *Aedeomyia squamipennis* has been implicated as a carrier of 24 virus strains serologically related to the prototype GAMV in Ecuador (Calisher et al. 1981; Mitchell et al. 1985).

The genus *Aedes* has 27 species that have been recorded in Ecuador. Some *Aedes* species pose a direct threat to human health, while others have been implicated in disease transmission in wildlife or act as secondary vectors under specific ecological conditions (Das et al. 2018). Aedes (Stegomyia) aegypti has been recorded on both the eastern and western continental slopes of the Andes, as well as in the Galápagos Islands. This species has been recorded in locations at elevations of 1,650 meters above sea level. Renowned for its role in virus transmission, *Aedes aegypti* is a major vector of Chikungunya virus (CHIKV), Yellow Fever virus (YFV), all serotypes of Dengue virus (DENV), and Zika virus (ZIKV), significantly impacting human health (Cevallos et al. 2018; Ogunlade et al. 2021). Additionally, studies have reported midgut infections of this species with Sindbis virus (SINV) (Saredy et al. 2020). *Aedes aegypti* is also suspected to serve as vector of *Wuchereria bancrofti*, the causative agent of lymphatic filariasis (Paily et al. 2006).

*Aedes albopictus* was first recorded in Ecuador in 2017 in Guayaquil and has since expanded to the Amazon basin and northwestern lowlands (Ponce et al. 2018). Field studies in the Americas have detected multiple arboviruses in Aedes (Stegomyia) albopictus, including Eastern equine encephalitis virus (EEEV), Potosi virus (POTV), Keystone virus (KSV), Tensaw virus (TENV) (Mitchell et al. 1992), Cache Valley virus (CVV) (Mitchell et al. 1998), West Nile virus (WNV) (Rothman et al. 2021), Usutu virus (USUV) (Puggioli et al. 2017), all serotypes of DENV (Ferreira-de-Lima and Lima-Camara 2018), YFV, CHIKV and ZIKV (Grard et al. 2014), highlighting its potential to infect humans and animals (Cancrini et al. 2003). Experimental evidence confirms its competence as a vector for DENV, ZIKV, WNV, YFV, CHIKV, EEEV, VEEV, Mayaro virus (MAYV), Ross River virus (RRV), SINV, Western equine encephalitis virus (WEEV), Jamestown Canyon virus (JCV), KSV, La Crosse virus (LACV), and Rift Valley virus (RVFV) (Pereira-dos-Santos et al. 2020). Notably EEEV and KSV were the first arboviruses isolated from this species, initially detected in tire dumps in Florida (Mitchell et al. 1992). *Aedes albopictus* is also a vector of filarial parasites, reinforcing its epidemiological significance (Cancrini et al. 2003).

Aedes (Ochlerotatus) taeniorhynchus is a coastal mosquito that lives in salty marshes, capable of forming large swarms. This species is a vector of VEEV (Weaver et al. 1996; Rivas et al. 1997). In the Galápagos, this species is a competent and efficient vector of WNV. Unlike mainland populations, the Galápagos strain shows significant genetic divergence, suggesting it may be a distinct, locally adapted species with unique vector ecology (Eastwood et al. 2013).

The genus *Haemagogus* has 13 species reported in Ecuador, including Haemagogus (Haemagogus) janthinomys and Haemagogus (Conopostegus) leucocelaenus, which are key vectors of YFV in sylvatic cycles, transmitting it between non-human primates and humans (Li et al. 2022). Haemagogus (Haemagogus) anastasionis is considered a potential vector of YFV and MAYV (Navarro et al. 2013a).

Fourteen *Psorophora* species have been recorded in Ecuador, including Psorophora (Janthinosoma) ferox which is a vector of Rocio virus (ROCV) (De Souza Lopes et al. 1981) and Ilehus virus (ILHV) (Johnson et al. 2007).

The genus *Culex* has 83 species in Ecuador, which represents the 8% of the world’s diversity. Culex (Culex) quinquefasciatus is a species with a broad distribution across the western and eastern regions of the Andean Mountain range in Ecuador. Additionally, it has been introduced into the Galápagos Islands, where it serves as a vector for avian malaria, the avian pox virus, and the WNV (Levi-Castillo 1958; Whiteman et al. 2005). Furthermore, this species is the primary agent responsible for the transmission of filariasis and has been implicated as a secondary vector of the Oropouche virus (OROV) (Consoli and Lourenço de Oliveira 1994).

Culex (Culex) nigripalpus, originally native to Brazil, has a widespread distribution in tropical and subtropical areas in North and South America. This mosquito species serves as a vector for multiple arboviruses, including Saint Louis encephalitis virus (SLEV), VEEV, EEEV, and WNV (Rutledge et al. 2003; Richards et al. 2012; De Carvalho et al. 2017).

Culex (Melanoconion) iolambdis, a mosquito species found throughout much of tropical America, is a putative vector in the transmission of VEEV (Scherer et al. 1971). The poor knowledge regarding their biology and ecology limits the current understanding of the potential importance of this mosquito species in the transmission of human pathogens (Blosser et al. 2016).

Culex (Melanoconion) ocossa has been identified as a carrier of a flavivirus in Perú (Evangelista et al. 2013) and is also associated with the VEEV (Turell et al. 1999; Weaver et al. 2004; Morrison et al. 2008).

The genus *Deinocerites* is represented in Ecuador by a single reported species, *Deinocerites pseudes*. This species has been suggested as a potential vector of VEEV and SLEV (Adames 1971).

Mosquitoes of the genus *Coquillettidia* have been identified as carriers of various pathogens, including avian malaria parasites (Njabo et al. 2009) and several arboviruses such as EEEV, WNV, SINV, MAYV, OROV, SLEV, Usutu virus (USUV), Tahyna virus (TAHV) and Batai virus (BATV) (Velásquez 2014; Lebl et al. 2015). In Ecuador, Coquillettidia (Rhynchotaenia) albicosta and Coquillettidia (Rhynchotaenia) venezuelensis have been reported. The former was identified as a carrier of Rochambeau virus (RBUV) in French Guiana in 1973 (Mahy and Van Regenmortel 2008), while the latter has been recognized as a potential vector for MAYV, OROV, and SLEV (Velásquez 2014).

In Ecuador, the genus *Mansonia* is represented by five species. However, research into their potential role as disease vectors remains scarce, despite evidence that some specimens harbor arboviruses and other pathogens. Mansonia (Mansonia) titillans has been identified as a carrier of VEEV (Weaver et al. 2004) and SLEV (Hoyos-López et al. 2015; Beranek et al. 2018) as well as a transporter of *Dermatobia hominis* eggs (Hervé et al. 1986; Lourenço-de-Oliveira and Heyden 1986). Additionally, the Bunyamwera serogroup, a significant subgroup within the *Orthobunyavirus* genus (Maes et al. 2018), has been reported in Mansonia (Mansonia) titillans from Argentina (Beranek et al. 2018). Furthermore, avian *Plasmodium* lineages have been found in Mansonia (Mansonia) titillans and Mansonia (Mansonia) pseudotitillans from Brazil (Ferreira et al. 2016).

Mansonia (Mansonia) indubitans has demonstrated moderate susceptibility to infection by four strains of VEEV. *Plasmodium nucleophilum* and Haemoproteus (Parahaemoproteus) spp. have been detected in this species (Turell et al. 1999; Méndez et al. 2001).

The genus *Johnbelkinia* has two species in Ecuador. Among them, *Johnbelkinia ulopus* has been mentioned as a potential vector of arbovirus like Triniti virus (TNTV) (Zavortink 1979; Machado-Allison et al. 1986; Naranjo-Díaz et al. 2022).

A total of 14 species of the genus *Sabethes* have been documented in Ecuador. Among them, Sabethes (Sabethoides) chloropterus has been reported as a vector of YFV (De Rodaniche and Galindo 1957), SLEV and ILHV (Hervé et al. 1986; Harbach 1994). Additionally, Sabethes (Sabethes) amazonicus and Sabethes (Sabethes) albiprivus have been identified as potential vectors of YFV (Navarro et al. 2013a; Cano et al. 2021).

## Conclusions

This study provides an update on the mosquito species of Ecuador, including insights into their taxonomy, distribution, and medical importance. Understanding the diversity and geographical distribution of these species is essential for vector control efforts aimed at mitigating disease transmission. However, the limited taxonomic research in Ecuador presents significant challenges for species identification and effective surveillance. The lack of standardized identification tools forces researchers to rely on external references that may not fully capture Ecuador’s unique mosquito fauna, further complicated by morphological variations influenced by ecological adaptations and geographic isolation.

To address these gaps, it is important to strengthen entomological studies, develop reliable identification resources, and improve local taxonomic expertise. Enhancing mosquito surveillance and research will not only refine species classification but also contribute to better disease prevention strategies, ultimately supporting public health efforts in the region.

## Additional information

### Use of AI

No use of AI was reported.

### Author ORCIDs

Patricio Ponce https://orcid.org/0000-0002-7627-1056

Varsovia Cevallos https://orcid.org/0000-0001-8666-1536

Andrés Carrazco-Montalvo https://orcid.org/0000-0002-3581-7761

Jennifer Gallardo-Cóndor https://orcid.org/0000-0001-7867-5372

Valentina Arévalo https://orcid.org/0000-0002-0088-4200

Ximena Galarza https://orcid.org/0000-0001-9181-3354

Josefina Coloma https://orcid.org/0000-0003-0222-6487

### Data availability

All of the data that support the findings of this study are available in the main text or Supplementary Information.

## Supplementary materials

Supplementary material 1 Geographic coordinates of new record collections Data type xlsx https://binary.pensoft.net/file/1554060This dataset is made available under the Open Database License (http://opendatacommons.org/licenses/odbl/1.0/). The Open Database License (ODbL) is a license agreement intended to allow users to freely share, modify, and use this Dataset while maintaining this same freedom for others, provided that the original source and author(s) are credited. Patricio Ponce, Varsovia Cevallos, Andrés Carrazco-Montalvo, Jennifer Gallardo-Cóndor, Valentina Arévalo, Ximena Galarza, Josefina Coloma

Supplementary material 2 Classification of mosquito species by vectorial evidence Data type docx https://binary.pensoft.net/file/1554061This dataset is made available under the Open Database License (http://opendatacommons.org/licenses/odbl/1.0/). The Open Database License (ODbL) is a license agreement intended to allow users to freely share, modify, and use this Dataset while maintaining this same freedom for others, provided that the original source and author(s) are credited. Patricio Ponce, Varsovia Cevallos, Andrés Carrazco-Montalvo, Jennifer Gallardo-Cóndor, Valentina Arévalo, Ximena Galarza, Josefina Coloma

## References

[B1] Adames AJ (1971) Mosquito Studies (Diptera, Culicidae): A Revision of the Crabhole Mosquitoes of the Genus *Deinocerites*. Contributions of the American Entomological Institute 7: 1–154.

[B2] Adames A, Arzube M (1975) Geographical extension of *Galindomyia Zeei* Stone and Barreto to Ecuador (Diptera: Culicidae). Mosquito Systematics 7: 113–114.

[B3] AFPMB (1998) Disease Vector Ecology Profile: Ecuador. United States Department of Defense, Office of the Deputy Under Secretary of Defense (Installations and Environment), Washington, DC, 38 pp.

[B4] Alencar RM, Sepulveda CCP, Martinez-Villegas L, Bahia AC, Santana RA, De Souza IB, D’Elia GMA, Duarte APM, De Lacerda MVG, Monteiro WM, Secundino NFC, Pimenta PFP, Koerich LB (2023) Unravelling the genome of the brackish water malaria vector *Anopheles aquasalis*. Scientific Reports 13: 20472. 10.1038/s41598-023-47830-1 PMC10665375 37993652

[B5] Arnell JH (1973) Mosquito Studies (Diptera, Culicidae). XXXII. A revision of the genus *Haemagogus*. Contributions of the American Entomological Institute (Ann Arbor) 10: 1–174.

[B6] Arnell JH (1976) Mosquito studies (Diptera, Culicidae) XXXIII. A revision of the Scapularis group of Aedes (Ochlerotatus). Contributions of the American Entomological Institute 13: 1–144.

[B7] Arregui G, Enriquez S, Benítez-Ortiz W, Navarro J-C (2015) Taxonomía molecular de *Anopheles* del Ecuador mediante ADN mitocondrial (Citocromo c Oxidasa I) y optimización por Parsimonia Máxima. Boletín de Malariología y Salud Ambiental 2: 132–154.

[B8] Ashfaq M, Hebert PDN, Mirza JH, Khan AM, Zafar Y, Mirza MS (2014) Analyzing Mosquito (Diptera: Culicidae) Diversity in Pakistan by DNA Barcoding. Boudko D (Ed.). PLoS ONE 9: e97268. 10.1371/journal.pone.0097268 PMC4036727 24827460

[B9] Ayala-Sulca Y, Carrasco-Badajoz C, Ramírez R, Iannacone J (2021) Diversidad y distribución de mosquitos (Diptera: Culicidae) en el Perú y su relación con las enfermedades metaxénicas. Revista de la Facultad de Medicina 70: e92324. 10.15446/revfacmed.v70n3.92324

[B10] Bánki O, Roskov Y, Döring M, Ower G, Hernández Robles DR, Plata Corredor CA, Stjernegaard Jeppesen T, Örn A, Pape T, Hobern D, Garnett S, Little H, DeWalt RE, Miller J, Orrell T, Aalbu R, Abbott J, Abreu C, Acero PA (2025) Catalogue of Life (2025-04-24 XR). Catalogue of Life Foundation, Amsterdam, Netherlands. 10.48580/dgvbl

[B11] Bataille A, Cunningham AA, Cedeño V, Cruz M, Eastwood G, Fonseca DM, Causton CE, Azuero R, Loayza J, Martinez JDC, Goodman SJ (2009) Evidence for regular ongoing introductions of mosquito disease vectors into the Galápagos Islands. Proceedings of the Royal Society B: Biological Sciences 276: 3769–3775. 10.1098/rspb.2009.0998 PMC2817279 19675009

[B12] Bataille A, Fournié G, Cruz M, Cedeño V, Parker PG, Cunningham AA, Goodman SJ (2012) Host selection and parasite infection in *Aedes taeniorhynchus*, endemic disease vector in the Galápagos Islands. Infection, Genetics and Evolution 12: 1831–1841. 10.1016/j.meegid.2012.07.019 22921730

[B13] Beebe NW (2018) DNA barcoding mosquitoes: advice for potential prospectors. Parasitology 145: 622–633. 10.1017/S0031182018000343 29564995

[B14] Beebe NW, Saul A (1995) Discrimination of all Members of the *Anopheles punctulatus* Complex by Polymerase Chain Reaction-Restriction Fragment Length Polymorphism Analysis. The American Journal of Tropical Medicine and Hygiene 53: 478–481. 10.4269/ajtmh.1995.53.478 7485705

[B15] Belkin JN (1962) The mosquitoes of the South Pacific (Diptera, Culicidae). University of California Press, Berkeley and Los Angeles, 412 pp.

[B16] Belkin J (1968) The type specimens of New World mosquitoes in European museums. Contributions of the American Entomological Institute 3: 1–72.

[B17] Belkin J, Schick R, Heinemann S (1965a) V. Mosquitoes originally described from Middle America. Contributions of the American Entomological Institute 1: 1–95.

[B18] Belkin JN, Hogue CL, Galindo P, Aitken TH, Schick RX, Powder WA (1965b) Mosquito Studies (Diptera, Culicidae) II. Methods for the collection, rearing and preservation of mosquitoes. Contributions of the American Entomological Institute 1: 19–78.

[B19] Beranek MD, Gallardo R, Almirón WR, Contigiani MS (2018) First detection of *Mansonia titillans* (Diptera: Culicidae) infected with St. Louis encephalitis virus (Flaviviridae: Flavivirus) and Bunyamwera serogroup (Peribunyaviridae: Orthobunyavirus) in Argentina. Journal of Vector Ecology 43: 340–343. 10.1111/jvec.12320 30408293

[B20] Berlin O (1969) Mosquito Studies (Diptera, Culicidae) XII. A Revision of the Neotropical Subgenus *Howardina* of *Aedes*. Contributions of the American Entomological Institute 4: 1–190.

[B21] Berlin O, Belkin JN (1980) Mosquito studies (Diptera, Culicidae) XXXVI. Subgenera *Aedinus*, *Tinolestes* and *Anoedioporpa* of *Culex*. 17: 1–104.

[B22] Blosser EM, Stenn T, Acevedo C, Burkett-Cadena ND (2016) Host use and seasonality of *Culex* (Melanoconion) iolambdis (Diptera: Culicidae) from eastern Florida, USA. Acta Tropica 164: 352–359. 10.1016/j.actatropica.2016.10.001 27712940

[B23] Bourke BP, Oliveira TP, Suesdek L, Bergo ES, Sallum MAM (2013) A multi-locus approach to barcoding in the *Anopheles strodei* subgroup (Diptera: Culicidae). Parasites & Vectors 6: 111. 10.1186/1756-3305-6-111 PMC3641011 23597081

[B24] Bram RA (1967) Classification of *Culex* subgenus Culex in the New World (Diptera: Culicidae). Proceedings of the United States National Museum 120: 1–122. 10.5479/si.00963801.120-3557.1

[B25] Brochero H, Li C, Wilkerson R, Conn JE, Ruiz-García M (2010) Genetic Structure of *Anopheles* (Nyssorhynchus) marajoara (Diptera: Culicidae) in Colombia. The American Society of Tropical Medicine and Hygiene 83: 585–595. 10.4269/ajtmh.2010.09-0482

[B26] Burkett-Cadena ND, Blosser EM (2017) *Aedeomyia squamipennis* (Diptera: Culicidae) in Florida, USA, a New State and Country Record. Journal of Medical Entomology 54: 788–792. 10.1093/jme/tjw226 28399225

[B27] Calisher CH, Lazuick JS, Justines G, Francy DB, Monath TP, V EG, Sabattini MS, Bowen GS, Jakob WL (1981) Viruses Isolated from *Aedeomyia Squamipennis* Mosquitoes Collected in Panama, Ecuador, and Argentina: Establishment of the Gamboa Serogroup. The American Journal of Tropical Medicine and Hygiene 30: 219–223. 10.4269/ajtmh.1981.30.219 6111232

[B28] Campos FR (1921) Especies nuevas de insectos ecuatorianos. Revista del Colegio Nacional Vicente Rocafuerte 3: 84–92.

[B29] Campos FR (1922) Estudios sobre la Fauna Entomológica del Ecuador. 2° Dipteros Nematóceros: Fam. Culicidae (Mosquitos). Revista del Colegio Nacional Vicente Rocafuerte 1: 18–30.

[B30] Campos FR (1924) Un año a caza de criaderos de mosquitos por los pantanos de Guayaquil y sus alrededores. Revista del Colegio Nacional Vicente Rocafuerte 1: 17–27.

[B31] Campos FR (1925) Estudios biológicos sobre los mosquitos de Guayaquil y alrededores. Revista del Colegio Nacional Vicente Rocafuerte 7: 21–22.

[B32] Cañadas L (1983) El mapa Bioclimático y Ecológico del Ecuador. Banco Central del Ecuador. Quito, 210 pp.

[B33] Cancrini G, Frangipane Di Regalbono A, Ricci I, Tessarin C, Gabrielli S, Pietrobelli M (2003) *Aedes albopictus* is a natural vector of *Dirofilaria immitis* in Italy. Veterinary Parasitology 118: 195–202. 10.1016/j.vetpar.2003.10.011 14729167

[B34] Cano ME, Marti GA, Balsalobre A, Muttis E, Bruno EA, Rossi G, Micieli MV (2021) Database of Sabethes and Haemagogus (Diptera: Culicidae) in Argentina: Sylvatic Vectors of the Yellow Fever Virus. Journal of Medical Entomology 58: 1762–1770. 10.1093/jme/tjab059 33905516

[B35] Cevallos V, Ponce P, Waggoner JJ, Pinsky BA, Coloma J, Quiroga C, Morales D, Cárdenas MJ (2018) Zika and Chikungunya virus detection in naturally infected *Aedes aegypti* in Ecuador. Acta Tropica 177: 74–80. 10.1016/j.actatropica.2017.09.029 28982578

[B36] Chakraborty C, Sharma AR, Sharma G, Bhattacharya M, Patra BC, Sarkar BK, Banerjee S, Banerjee K, Lee S-S (2021) Understanding the molecular evolution of tiger diversity through DNA barcoding marker ND4 and NADH dehydrogenase complex using computational biology. Genes & Genomics 43: 759–773. 10.1007/s13258-021-01089-w 33884571

[B37] Consoli RAGB, Lourenço de Oliveira R (1994) Principais mosquitos de importância sanitária no Brasil. Coimbra C, Bori C, Pessanha C, Momen H, Benchimol J, da Rocha Carvalheiro J, Ferreira LF, Struchiner M, Gadelha P, Amarante P, Marchiori Buss P, Macedo V, Brenner Z (Eds). FIOCRUZ, Río de Janeiro, 228 pp. 10.7476/9788575412909

[B38] Das B, Ghosal S, Mohanty S (2018) *Aedes*: What Do We Know about Them and What Can They Transmit? In: Savić S (Ed.), Vectors and Vector-Borne Zoonotic Diseases. IntechOpen, London, 6–17. 10.5772/intechopen.81363

[B39] De Carvalho GC, Vendrami DP, Marrelli MT, Wilke ABB (2017) Wing variation in *Culex nigripalpus* (Diptera: Culicidae) in urban parks. Parasites & Vectors 10: 423. 10.1186/s13071-017-2348-5 PMC5604421 28923116

[B40] De Souza Lopes O, De Sacchetta LA, Francy DB, Jakob WL, Calisher CH (1981) Emergence of a new arbovirus disease in Brazil. American Journal of Epidemiology 113: 122–125. 10.1093/oxfordjournals.aje.a113075 6110335

[B41] De Rodaniche E, Galindo P (1957) Isolation of Yellow Fever Virus from *Haemagogus Mesodentatus*, *H. Equinus* and *Sabethes Chloropterus* Captured in Guatemala in 1956. The American Journal of Tropical Medicine and Hygiene 6: 232–237. 10.4269/ajtmh.1957.6.232 13424899

[B42] Eastwood G, Goodman SJ, Cunningham AA, Kramer LD (2013) *AedesTaeniorhynchus* Vectorial Capacity Informs A Pre-Emptive Assessment Of West Nile Virus Establishment In Galápagos. Scientific Reports 3: 1519. 10.1038/srep01519 PMC3605609 23519190

[B43] Escovar JE, González R, Quiñones ML, Wilkerson RC, Ruiz F, Harrison BA (2014) Morphology of the larvae, male genitalia and DNA sequences of *Anopheles* (Kerteszia) pholidotus (Diptera: Culicidae) from Colombia. Memórias do Instituto Oswaldo Cruz 109: 473–479. 10.1590/0074-0276130596 PMC4155850 25075785

[B44] Evangelista J, Cruz C, Guevara C, Astete H, Carey C, Kochel TJ, Morrison AC, Williams M, Halsey ES, Forshey BM (2013) Characterization of a novel flavivirus isolated from *Culex* (Melanoconion) ocossa mosquitoes from Iquitos, Peru. Journal of General Virology 94: 1266–1272. 10.1099/vir.0.050575-0 23515021

[B45] Faran M (1979) Anopheles*(Nyssorhynchus) trinkae*, a new species in the *Albimanus* Section (Diptera: Culicidae). Mosquito Systematics 11: 26–39.

[B46] Faran ME (1980) Mosquito studies (Diptera, Culicidae). XXXIV. A revision of the *Albimanus* section of the subgenus Nyssorhynchus of *Anopheles*. Contributions of the American Entomological Institute (Ann Arbor) 15: 1–215.

[B47] Faran ME, Linthicum KJ (1981) A handbook of the Amazonian species of *Anopheles* (Nyssorhynchus) (Diptera: Culicidae). Mosquito Systematic 13: 1–81. 10.5281/zenodo.15903090

[B48] Ferreira FC, Rodrigues RA, Sato Y, Borges MAZ, Braga ÉM (2016) Searching for putative avian malaria vectors in a Seasonally Dry Tropical Forest in Brazil. Parasites & Vectors 9: 587. 10.1186/s13071-016-1865-y PMC5112751 27852326

[B49] Ferreira-de-Lima VH, Lima-Camara TN (2018) Natural vertical transmission of dengue virus in *Aedes aegypti* and *Aedes albopictus*: a systematic review. Parasites & Vectors 11: 77. 10.1186/s13071-018-2643-9 PMC5793400 29391071

[B50] Foley DH, Rueda LM, Wilkerson RC (2007) Insight into global mosquito biogeography from country species records. Journal of Medical Entomology 44: 554–567. 10.1093/jmedent/44.4.554 17695008

[B51] Folmer O, Black M, Hoeh W, Lutz R, Vrijenhoek R (1994) DNA primers for amplification of mitochondrial cytochrome c oxidase subunit I from diverse metazoan invertebrates. Molecular Marine Biology and Biotechnology 3: 294–299. 7881515

[B52] Forattini OP (1965) 2 Entomología Médica. Faculdade de Higiene e Saude Publica, São Paulo, Brazil, 46 pp.

[B53] Forattini OP, Sallum MAM (1992) A new species of Culex (Melanoconion) from the Amazonian Region (Diptera: Culicidae). Memórias do Instituto Oswaldo Cruz 87: 265–274. 10.1590/S0074-02761992000200015

[B54] Foster PG, Bergo ES, Bourke BP, Oliveira TMP, Nagaki SS, Sant’Ana DC, Sallum MAM (2013) Phylogenetic Analysis and DNA-based Species Confirmation in Anopheles (Nyssorhynchus). Moreira LA (Ed.). PLoS ONE 8: e54063. 10.1371/journal.pone.0054063 PMC3563636 23390494

[B55] Fritz GN, Engman S, Rodriguez R, Wilkerson RC (2004) Identification of Four Vectors of Human *Plasmodium* spp. by Multiplex PCR: *Anopheles rangeli*, *An. strodei*, *An. triannulatus*, and *An. trinkae* (Diptera: Culicidae: *Nyssorhynchus*). Journal of Medical Entomology 41: 1111–1115. 10.1603/0022-2585-41.6.1111 15605651

[B56] Gabaldon A, Cova-Garcia P (1952) Zoogeografía de los anofelinos en Venezuela. IV. Su posición en la región Neotrópica y observaciones sobre las especies de esta región. Revista de Sanidad y Asistencia Social 17: 171–209. 13121570

[B57] GBIF-Secretariat (2025) Global Biodiversity Information Repository. Universitet sparken, Copenhagen, Denmark. https://www.gbif.org/es/ [accessed March 5, 2025]

[B58] González R, Carrejo N, Wilkerson RC, Alarcon J, Alarcon-Ormasa J, Ruiz F, Bhatia R, Loaiza J, Linton Y-M (2010) Confirmation of Anopheles (Anopheles) calderoni Wilkerson, 1991 (Diptera: Culicidae) in Colombia and Ecuador through molecular and morphological correlation with topotypic material. Memórias do Instituto Oswaldo Cruz 105: 1001–1009. 10.1590/S0074-02762010000800009 21225197

[B59] Gorham JR, Stojanovich CJ, Scott HG (1973) Clave Ilustrada para los Mosquitos Anofelinos de Sudamérica Occidental. Mosquito Systematics 5: 97–156.

[B60] Grard G, Caron M, Mombo IM, Nkoghe D, Mboui Ondo S, Jiolle D, Fontenille D, Paupy C, Leroy EM (2014) Zika Virus in Gabon (Central Africa) – 2007: A New Threat from *Aedes albopictus*? Charrel R (Ed.). PLoS Neglected Tropical Diseases 8: e2681. 10.1371/journal.pntd.0002681 PMC3916288 24516683

[B61] Gutiérrez-Vera E, Patiño L, Castillo-Segovia M, Mora-Valencia V, Montesdeoca-Agurto J, Regato-Arrata M (2021) Seroprevalence of arboviruses in Ecuador: Implications for improved surveillance. Biomédica 41: 247–259. 10.7705/biomedica.5623 PMC8382292 34214266

[B62] Hall CR, Howard TM, Harbach RE (1999) Sabethes (Peytonulus) luxodens, a New Species of Sabethini (Diptera: Culicidae) from Ecuador. Memórias do Instituto Oswaldo Cruz 94: 329–338. 10.1590/S0074-02761999000300009 10419382

[B63] Harbach RE (1994) The subgenus *Sabethinus* of *Sabethes* (Diptera: Culicidae). Systematic Entomology 19: 207–234. 10.1111/j.1365-3113.1994.tb00588.x

[B64] Harbach RE (2007) The Culicidae (Diptera): a review of taxonomy, classification and phylogeny*. Zootaxa 1668: 591–638. 10.11646/zootaxa.1668.1.28

[B65] Harbach RE (2013) The Phylogeny and Classification of *Anopheles*. In: Manguin S (Ed.), *Anopheles* mosquitoes - New insights into malaria vectors. InTech, London, 830. 10.5772/54695

[B66] Harbach RE (2025) Mosquito Taxonomic Inventory. http://mosquitosxonomic-inventory.info/ [accessed May 5, 2025]

[B67] Harbach RE, Howard TM (2009) Review of the genus *Chagasia* (Diptera: Culicidae: Anophelinae). Zootaxa 2210: 1–25. 10.11646/zootaxa.2210.1.1

[B68] Harbach RE, Kitching IJ (1998) Phylogeny and classification of the Culicidae (Diptera). Systematic Entomology 23: 327–370. 10.1046/j.1365-3113.1998.00072.x

[B69] Harbach RE, Peyton EL (2000) Systematics of *Onirion*, a new genus of Sabethini (Diptera: Culicidae) from the Neotropical Region. Bulletin of the Natural History Museum. Entomology series 69: 115–169.

[B70] Harrison B, Ruiz-Lopez F, Calderon G (2012) Anopheles (Kerteszia) lepidotus (Diptera: Culicidae), not the malaria vector we thought it was: Revised male and female morphology; larva, pupa, and male genitalia characters; and molecular verification. Zootaxa 3218: 1–17. 10.11646/zootaxa.3218.1.1 PMC4696864 26726290

[B71] Hayes J, Calderon G, Falcon R, Zambrano V (1987) Newly incriminated anopheline vectors of human malaria parasites in Junin Department, Peru. Journal of the American Mosquito Control Association 3: 418–422. 3333060

[B72] Hebert PDN, Cywinska A, Ball SL, deWaard JR (2003) Biological identifications through DNA barcodes. Proceedings of the Royal Society of London. Series B: Biological Sciences 270: 313–321. 10.1098/rspb.2002.2218 PMC1691236 12614582

[B73] Heinemann SJ, Belkin JN (1979) Collection records of the project “Mosquitoes of Middle America”. 13. South America: Brazil (BRA, BRAP, BRB), Ecuador (ECU), Peru (PER), Chile (CH). Mosquito Systematics 11: 61–118. 10.5281/zenodo.15932108

[B74] Hernández-Triana LM, Brugman VA, Nikolova NI, Ruiz-Arrondo I, Barrero E, Thorne L, De Marco MF, Krüger A, Lumley S, Johnson N, Fooks AR (2019) DNA barcoding of British mosquitoes (Diptera, Culicidae) to support species identification, discovery of cryptic genetic diversity and monitoring invasive species. ZooKeys 832: 57–76. 10.3897/zookeys.832.32257 PMC6435598 30930645

[B75] Hernandez-Valencia JC, Muñoz-Laiton P, Gómez GF, Correa MM (2023) A Systematic Review on the Viruses of Anopheles Mosquitoes: The Potential Importance for Public Health. Tropical Medicine and Infectious Disease 8: 459. 10.3390/tropicalmed8100459 PMC10610971 37888587

[B76] Hervé J, Dégallier N, Travassos Da Rosa A, Pinheiro F, Sá Filho G (1986) Arboviroses. Aspectos ecológicos. In: Instituto Evandro Chagas. 50 años de contribuição às Ciências Biológicas e à Medicina Tropical. Fundação Serviços de Saúde Pública, Río de Janeiro, 529–556.

[B77] Holdridge LR, Grenke WC, Hatheway WH, Liang T, Tosi JA (1971) Forest environments in tropical life zones, a pilot study. Pergamon press, Oxford, 747 pp.

[B78] Hoyos-López R, Soto SU, Rúa-Uribe G, Gallego-Gómez JC (2015) Molecular identification of Saint Louis encephalitis virus genotype IV in Colombia. Memórias do Instituto Oswaldo Cruz 110: 719–725. 10.1590/0074-02760280040 PMC4667573 26313538

[B79] Jacome L, Liria J, Wilkerson R (2023) Species diversity and spatial distribution of mosquitoes (Diptera: Culicidae) from La Isla Amazon Park, Napo Province, Ecuador. Revista de Biología Tropical 71: e55184. 10.15517/rev.biol.trop..v71i1.55184

[B80] Jawień P, Pfitzner WP, Schaffner F, Kiewra D (2024) Mosquitoes (Diptera: Culicidae) of Poland: An Update of Species Diversity and Current Challenges. Insects 15: 353. 10.3390/insects15050353 PMC11122502 38786909

[B81] Johnson BW, Cruz C, Felices V, Espinoza WR, Manock SR, Guevara C, Olson JG, Kochel TJ (2007) *Ilheus Virus* Isolate from a Human, Ecuador. Emerging Infectious Diseases 13: 956–958. 10.3201/eid1306.070118 PMC2792834 17582910

[B82] Kirchgatter K, De Oliveira Guimarães L, Hugo Yañez Trujillano H, Rafael Arias F, Cáceres A, De Castro Duarte A, Dos Santos Malafronte R, Tubaki R, Mureb Sallum M (2020) Phylogeny of Anopheles (Kerteszia) (Diptera: Culicidae) Using Mitochondrial Genes. Insects 11: 324. 10.3390/insects11050324 PMC7290731 32456322

[B83] Kitching IJ, Lorna Culverwell C, Harbach RE (2015) The phylogenetic conundrum of *Lutzia* (Diptera: Culicidae: Culicini): a cautionary account of conflict and support. Insect Systematics & Evolution 46: 269–290. 10.1163/1876312X-45032119

[B84] Knight KL, Stone A (1977) A Catalog of the mosquitoes of the World (Diptera: Culicidae). Entomological Society of America, Press Thomas Say Foundation, Maryland, 611 pp.

[B85] Kroeger A, Mancheno M, Alarcon J, Pesse K (1995) Insecticide-Impregnated Bed Nets for Malaria Control: Varying Experiences from Ecuador, Colombia, and Peru Concerning Acceptability and Effectiveness. The American Journal of Tropical Medicine and Hygiene 53: 313–323. 10.4269/ajtmh.1995.53.313 7485681

[B86] Kumar NP, Rajavel AR, Natarajan R, Jambulingam P (2007) DNA Barcodes Can Distinguish Species of Indian Mosquitoes (Diptera: Culicidae). Journal of Medical Entomology 44: 01–07. 10.1093/jmedent/41.5.01 17294914

[B87] Lane J (1953) Neotropical Culicidae Vol. 1. University of São Paulo, São Paulo, 1055 pp.

[B88] Laurito M, Oliveira TMD, Almiron WR, Sallum MAM (2013) COI barcode versus morphological identification of Culex (Culex) (Diptera: Culicidae) species: a case study using samples from Argentina and Brazil. Memórias do Instituto Oswaldo Cruz 108: 110–122. 10.1590/0074-0276130457 PMC4109187 24473810

[B89] Laurito M, Ayala AM, Arias-Builes DL, Almirón WR (2022) Improving the DNA Barcode Library of Mosquito Species With New Identifications and Discoveries in North-Central Argentina. Slotman M (Ed.). Journal of Medical Entomology 59: 173–183. 10.1093/jme/tjab160 34661674

[B90] Lebl K, Zittra C, Silbermayr K, Obwaller A, Berer D, Brugger K, Walter M, Pinior B, Fuehrer H-P, Rubel F (2015) Mosquitoes (Diptera: Culicidae) and their relevance as disease vectors in the city of Vienna, Austria. Parasitology Research 114: 707–713. 10.1007/s00436-014-4237-6 PMC4303709 25468380

[B91] Levi-Castillo R (1945a) *Anopheles pseudopunctipennis* in the Los Chillos Valley of Ecuador. Journal of Economic Entomology 38: 385–388. 10.1093/jee/38.3.385

[B92] Levi-Castillo R (1945b) Catálogo de los Anofelinos de la República del Ecuador. Artes Gráficas Senefelder C.A., Guayaquil, Ecuador, 172 pp.

[B93] Levi-Castillo R (1949a) Atlas de los anofelinos sudamericanos. Sociedad Filantrópica del Guayas, Guayaquil, Ecuador, 207 pp.

[B94] Levi-Castillo R (1949b) Lista provisional de los mosquitos *Culex* del Ecuador. Physis 20: 190–193.

[B95] Levi-Castillo R (1952a) Redescription of Aedes (Ochlerotatus) camposanus Dyar (1918) as a Valid Species Found in the Coastal Plain of Ecuador. Pacific Science 6: 262–264.

[B96] Levi-Castillo R (1952b) Vorläufige Liste der Stechmücken (Uranofaeniini, Toxorhynchitini, Culicini, Aedini und Sabethini) aus Ecuador (Diptera-Culicidae). Zeitschrift für Tropenmedizin und Parasitologie 3: 552–559. 14959282

[B97] Levi-Castillo R (1953a) A new species of *Culex* from Ecuador. Proceedings of the Entomological Society of Washington 55: 161–163.

[B98] Levi-Castillo R (1953b) Dos especies nuevas de mosquitos de la sierra ecuatoriana (Diptera-Culicidae). Revista Ecuatoriana de Entomología y Parasitología 1: 63–70.

[B99] Levi-Castillo R (1953c) Lista provisional y distribución de los mosquitos Culicinos en el Ecuador. Revista Ecuatoriana de Entomología y Parasitología: 34–45.

[B100] Levi-Castillo R (1953d) Observations on the Subgenus *Phalangomyia* of the Genus *Culex* in Ecuador with description of a new species (Diptera, Culicidae). Pacific Science 7: 187–192.

[B101] Levi-Castillo R (1953e) *Toxohrynchites aequatorianus n. sp*., especie nueva de *Toxorhynchites* hallada en Pichilingue, Los Ríos Ecuador (Diptera-Culicidae). Revista Ecuatoriana de Entomología y Parasitología 1: 71–79.

[B102] Levi-Castillo R (1953f) Una nueva especie de *Culex* procedente de la Provincia de Los Ríos, Ecuador: *Culex (Carrollia) babahoyensis n. sp*. (Diptera-Culicidae). Revista Ecuatoriana de Entomología y Parasitología 1: 91–94.

[B103] Levi-Castillo R (1954) Cuatro especies nuevas de Sabethini del Ecuador (Diptera-Culicidae). Revista Ecuatoriana de Entomología y Parasitología 2: 247–260.

[B104] Levi-Castillo R (1955a) *Haemagogus soperi n. sp*. mosquito transmisor de Fiebre Amarilla Selvática en el Ecuador (Diptera-Culicidae). Revista Ecuatoriana de Entomología y Parasitología 2: 479–485.

[B105] Levi-Castillo R (1955b) *Phoniomyia esmeraldasi*, a new mosquito from Ecuador. Revista Ecuatoriana de Entomología y Parasitología 2: 389–392.

[B106] Levi-Castillo R (1955c) Un nuevo Anofelino de altura del Ecuador: *Anophelesgomezdelatorrei n.sp*. Revista Ecuatoriana de Entomología y Parasitología 2: 509–516.

[B107] Levi-Castillo R (1958) Provisional list of the Culicidae, Simuliidae, Phlebotomus and Culicoides of Ecuador. Proceedings of the Tenth International Congress of Entomology 3: 867–871. 10.5962/p.372162

[B108] Li SL, Acosta AL, Hill SC, Brady OJ, De Almeida MAB, Cardoso JDC, Hamlet A, Mucci LF, Telles De Deus J, Iani FCM, Alexander NS, Wint GRW, Pybus OG, Kraemer MUG, Faria NR, Messina JP (2022) Mapping environmental suitability of *Haemagogus* and *Sabethes* spp. mosquitoes to understand sylvatic transmission risk of yellow fever virus in Brazil. Wu JT (Ed.). PLOS Neglected Tropical Diseases 16: e0010019. 10.1371/journal.pntd.0010019 PMC8797211 34995277

[B109] Linton Y-M, Pecor JE, Porter CH, Mitchell LB, Garzon-Moreno A, Foley DH, Pecor DB, Wilkerson RC (2013) Mosquitoes of eastern Amazonian Ecuador: biodiversity, bionomics and barcodes. Memórias do Instituto Oswaldo Cruz 108: 100–109. 10.1590/0074-0276130440 PMC4109186 24473809

[B110] Lorenz C, Marques TC, Sallum MAM, Suesdek L (2012) Morphometrical diagnosis of the malaria vectors *Anopheles cruzii*, *An. homunculus* and *An. bellator*. Parasites & Vectors 5: 257. 10.1186/1756-3305-5-257 PMC3514230 23148743

[B111] Lounibos LP, Wilkerson RC, Conn JE, Hribar LJ, Fritz GN, Danoff-Burg JA (1998) Morphological, Molecular, and Chromosomal Discrimination of Cryptic Anopheles (Nyssorhynchus) (Diptera: Culicidae) from South America. Journal of Medical Entomology 35: 830–838. 10.1093/jmedent/35.5.830 9775617

[B112] Lourenço-de-Oliveira R, Heyden R (1986) Alguns aspectos da ecologia dos mosquitos (Diptera: Culicidae) de uma área de planície (granjas Calábria) em Jacarepaguá, Rio de Janeiro: IV. Preferências alimentares quanto ao hospedeiro e freqüência domiciliar. Memórias do Instituto Oswaldo Cruz 81: 15–27. 10.1590/S0074-02761986000100003 2883555

[B113] Loyola EG, Arredondo JI, Rodriguez MH, Brown DN, Vaca-Marin MA (1991) *Anopheles vestitipennis*, the probable vector of *Plasmodium vivax* in the Lacandon forest of Chiapas, México. Transactions of the Royal Society of Tropical Medicine and Hygiene 85: 171–174. 10.1016/0035-9203(91)90010-V 1887463

[B114] Machado-Allison CE, Barrera R, Delgado L, Gómez-Cova C, Navarro JC (1986) Mosquitos (Diptera: Culicidae) de los Fitotelmata de Panaquire, Venezuela. Acta Biologica Venezuelica 12: 1–12.

[B115] Maes P, Alkhovsky SV, Bào Y, Beer M, Birkhead M, Briese T, Buchmeier MJ, Calisher CH, Charrel RN, Choi IR, Clegg CS, De La Torre JC, Delwart E, DeRisi JL, Di Bello PL, Di Serio F, Digiaro M, Dolja VV, Drosten C, Druciarek TZ, Du J, Ebihara H, Elbeaino T, Gergerich RC, Gillis AN, Gonzalez J-PJ, Haenni A-L, Hepojoki J, Hetzel U, Hồ T, Hóng N, Jain RK, Jansen Van Vuren P, Jin Q, Jonson MG, Junglen S, Keller KE, Kemp A, Kipar A, Kondov NO, Koonin EV, Kormelink R, Korzyukov Y, Krupovic M, Lambert AJ, Laney AG, LeBreton M, Lukashevich IS, Marklewitz M, Markotter W, Martelli GP, Martin RR, Mielke-Ehret N, Mühlbach H-P, Navarro B, Ng TFF, Nunes MRT, Palacios G, Pawęska JT, Peters CJ, Plyusnin A, Radoshitzky SR, Romanowski V, Salmenperä P, Salvato MS, Sanfaçon H, Sasaya T, Schmaljohn C, Schneider BS, Shirako Y, Siddell S, Sironen TA, Stenglein MD, Storm N, Sudini H, Tesh RB, Tzanetakis IE, Uppala M, Vapalahti O, Vasilakis N, Walker PJ, Wáng G, Wáng L, Wáng Y, Wèi T, Wiley MR, Wolf YI, Wolfe ND, Wú Z, Xú W, Yang L, Yāng Z, Yeh S-D, Zhāng Y-Z, Zhèng Y, Zhou X, Zhū C, Zirkel F, Kuhn JH (2018) Taxonomy of the family Arenaviridae and the order Bunyavirales: update 2018. Archives of Virology 163: 2295–2310. 10.1007/s00705-018-3843-5 29680923

[B116] Mahy BWJ, Van Regenmortel MHV (2008) Encyclopedia of Virology. 3^rd^ edn. Academic Press, Amsterdam, Boston.

[B117] Martin H, Reeves LE, Steele G, Rosales A, Heinig R, Lucas KJ (2024) *Aedeomyia squamipennis*: A new genus and species record for Collier County, Florida, USA. Journal of the American Mosquito Control Association 40: 174–177. 10.2987/24-7188 39658836

[B118] Méndez W, Liria J, Navarro J-C, García CZ, Freier JE, Salas R, Weaver SC, Barrera R (2001) Spatial Dispersion of Adult Mosquitoes (Diptera: Culicidae) in a Sylvatic Focus of Venezuelan Equine Encephalitis Virus. Journal of Medical Entomology 38: 813–821. 10.1603/0022-2585-38.6.813 11761379

[B119] Mier-y-Teran-Romero L, Tatem AJ, Johansson MA (2017) Mosquitoes on a plane: Disinsection will not stop the spread of vector-borne pathogens, a simulation study. Rabaa MA (Ed.). PLOS Neglected Tropical Diseases 11: e0005683. 10.1371/journal.pntd.0005683 PMC5510898 28672006

[B120] Mitchell CJ, Monath TP, Sabattini MS, Cropp CB, Daffner JF, Calisher CH, Jakob WL, Christensen HA (1985) Arbovirus investigations in Argentina, 1977–1980. II. Arthropod collections and virus isolations from Argentine mosquitoes. The American Journal of Tropical Medicine and Hygiene 34: 945–955. 10.4269/ajtmh.1985.34.945 2863989

[B121] Mitchell CJ, Niebylski ML, Smith GC, Karabatsos N, Martin D, Mutebi JP, Craig GB, Mahler MJ (1992) Isolation of Eastern Equine Encephalitis Virus from *Aedes albopictus* in Florida. Science 257: 526–527. 10.1126/science.1321985 1321985

[B122] Mitchell CJ, Haramis LD, Karabatsos N, Smith GC, Starwalt VJ (1998) Isolation of La Crosse, Cache Valley, and Potosi Viruses from *Aedes* Mosquitoes (Diptera: Culicidae) Collected at Used-Tire Sites in Illinois During 1994–1995. Journal of Medical Entomology 35: 573–577. 10.1093/jmedent/35.4.573 9701947

[B123] Mojica J, Arévalo V, Juarez JG, Galarza X, Gonzalez K, Carrazco A, Suazo H, Harris E, Coloma J, Ponce P, Balmaseda A, Cevallos V (2025) A numbers game: mosquito-based arbovirus surveillance in two distinct geographic regions of Latin America. Healy K (Ed.). Journal of Medical Entomology 62: 220–224. 10.1093/jme/tjae121 PMC11735261 39308414

[B124] Moreno JE, Rubio-Palis Y, Páez E, Pérez E, Sánchez V, Vaccari E (2005) Anopheles (Anopheles) neomaculipalpus: a new malaria vector in the Amazon basin? Medical and Veterinary Entomology: 329–332. 10.1111/j.1365-2915.2005.00572.x 16134983

[B125] Morrison AC, Forshey BM, Notyce D, Astete H, Lopez V, Rocha C, Carrion R, Carey C, Eza D, Montgomery JM, Kochel TJ (2008) Venezuelan Equine Encephalitis Virus in Iquitos, Peru: Urban Transmission of a Sylvatic Strain. PLoS Neglected Tropical Diseases 2: e349. 10.1371/journal.pntd.0000349 PMC2593782 19079600

[B126] Naranjo-Díaz N, Correa MM (2025) An updated checklist of *Anopheles* (Diptera, Culicidae) of Colombia with new records and distribution data. ZooKeys 1231: 169–189. 10.3897/zookeys.1231.133711 PMC11923593 40114813

[B127] Naranjo-Díaz N, Suaza-Vasco J, Pineda-Angel J, Uribe S (2022) New records of Sabethini (Diptera: Culicidae) from Colombia. Biodiversity Data Journal 10: e68413. 10.3897/BDJ.10.e68413 PMC8831386 35153528

[B128] Navarro J-C, Ponce P, Cevallos V (2013a) Dos nuevos registros de vectores potenciales de Fiebre Amarilla selvática y Mayaro para el Ecuador. Boletín de Malariología y Salud Ambiental 53: 77–81.

[B129] Navarro J-C, Enriquez S, Vaca F, Benitez-Ortiz W (2013b) A New Phytotelm Plant, *Crinum moorei* (Asparagales: Amaryllidaceae), for the Americas and Its Mosquito Inhabitant (Diptera: Culicidae) in Ecuador. Florida Entomologist 96: 1224–1227. 10.1653/024.096.0374 PMC6759327 31551609

[B130] Navarro J-C, Arrivillaga J, Morales D, Ponce P, Cevallos V (2015) Evaluación rápida de biodiversidad de mosquitos (Diptera: Culicidae) y riesgo en salud ambiental en un área Montana del Chocó Ecuatoriano. Entomotropica 30: 160–173.

[B131] Navarro J-C, Enríquez S, Arrivillaga J, Benítez-Ortíz W (2016) Un nuevo *Aedes* para la Amazonía de Ecuador y actualización taxonómica del género para el país. Boletín de Malariología y Salud Ambiental 56: 113–121.

[B132] Nebbak A, Almeras L, Parola P, Bitam I (2022) Mosquito Vectors (Diptera: Culicidae) and Mosquito-Borne Diseases in North Africa. Insects 13: 962. 10.3390/insects13100962 PMC9604161 36292910

[B133] Neves A, Urbinatti PR, Malafronte RDS, Fernandes A, Paganini WDS, Natal D (2013) Malaria outside the Amazon region: Natural *Plasmodium* infection in anophelines collected near an indigenous village in the Vale do Rio Branco, Itanhaém, SP, Brazil. Acta Tropica 125: 102–106. 10.1016/j.actatropica.2012.08.014 22989665

[B134] Njabo KY, Cornel AJ, Sehgal RN, Loiseau C, Buermann W, Harrigan RJ, Pollinger J, Valkiūnas G, Smith TB (2009) *Coquillettidia* (Culicidae, Diptera) mosquitoes are natural vectors of avian malaria in Africa. Malaria Journal 8: 193–193. 10.1186/1475-2875-8-193 PMC3152766 19664282

[B135] Ogunlade ST, Meehan MT, Adekunle AI, Rojas DP, Adegboye OA, McBryde ES (2021) A Review: Aedes-Borne Arboviral Infections, Controls and Wolbachia-Based Strategies. Vaccines 9: 32–32. 10.3390/vaccines9010032 PMC7827552 33435566

[B136] Ortega-Morales AI, León-Espinosa GA, Rodríguez-Rojas JJ (2023) Updated checklist of the mosquitoes (Diptera: Culicidae) of Mexico. Journal of Vector Ecology 49: 28–43. 10.52707/1081-1710-49.1.28 38147299

[B137] Paily KP, Hoti SL, Balaraman K (2006) Development of Lymphatic Filarial Parasite *Wuchereria bancrofti* (Spirurida: Onchocercidae) in Mosquito Species (Diptera: Culicidae) Fed Artificially on Microfilaremic Blood. Journal of Medical Entomology 43: 1222–1226. 10.1093/jmedent/43.6.1222 17162957

[B138] Paskewitz SM, Collins FH (1990) Use of the polymerase chain reaction to identify mosquito species of the *Anopheles gambiae* complex. Medical and Veterinary Entomology 4: 367–373. 10.1111/j.1365-2915.1990.tb00453.x 2133004

[B139] Pecor J, Mallampalli V, Harbach RE, Peyton EL (1992) Catalog and illustrated review of the subgenus *Melanoconion* of *Culex* (Diptera: Culicidae). Contributions of the American Entomological Institute 27: 1–228. 10.21236/ada274863

[B140] Pereira-dos-Santos T, Roiz D, Lourenço-de-Oliveira R, Paupy C (2020) A Systematic Review: Is *Aedes albopictus* an Efficient Bridge Vector for Zoonotic Arboviruses? Pathogens 9: 266. 10.3390/pathogens9040266 PMC7238240 32272651

[B141] Pereira-Silva JW, Martins-Campos KM, Ferreira-Neto JV, Lacerda MVG, Pessoa FAC, Ríos-Velásquez CM (2022) Amazonian *Anopheles* with low numbers of oocysts transmit *Plasmodium vivax* sporozoites during a blood meal. Scientific Reports 12: 19442. 10.1038/s41598-022-24058-z PMC9663451 36376491

[B142] Pimenta PF, Orfano AS, Bahia AC, Duarte AP, Ríos-Velásquez CM, Melo FF, Pessoa FA, Oliveira GA, Campos KM, Villegas LM, Rodrigues NB, Nacif-Pimenta R, Simões RC, Monteiro WM, Amino R, Traub-Cseko YM, Lima JB, Barbosa MG, Lacerda MV, Tadei WP, Secundino NF (2015) An overview of malaria transmission from the perspective of Amazon *Anopheles* vectors. Memórias do Instituto Oswaldo Cruz 110: 23–47. 10.1590/0074-02760140266 PMC4371216 25742262

[B143] Pinault LL, Hunter FF (2011) New highland distribution records of multiple *Anopheles* species in the Ecuadorian Andes. Malaria Journal 10: 236. 10.1186/1475-2875-10-236 PMC3176254 21835004

[B144] Ponce P, Morales D, Argoti A, Cevallos VE (2018) First Report of *Aedes* (Stegomyia) albopictus (Skuse) (Diptera: Culicidae), the Asian Tiger Mosquito, in Ecuador. Journal of Medical Entomology 55: 248–249. 10.1093/jme/tjx165 PMC5850216 29029173

[B145] Pontual JDC, Coelho NV, Santos NACD, Bastos ADS, Araújo JE, Andrade AO, Medeiros JF, Araujo MDS (2025) Blood Source and Anesthetics Effects on the Maintenance of *Anopheles darlingi* in the Lab-Rearing Condition. Insects 16: 281. 10.3390/insects16030281 PMC11942927 40266770

[B146] Puggioli A, Bonilauri P, Calzolari M, Lelli D, Carrieri M, Urbanelli S, Pudar D, Bellini R (2017) Does *Aedes albopictus* (Diptera: Culicidae) play any role in Usutu virus transmission in Northern Italy? Experimental oral infection and field evidences. Acta Tropica 172: 192–196. 10.1016/j.actatropica.2017.05.006 28495404

[B147] Quinatoa Tutillo P, Bustillos JJ, Mora JP, Padilla AN, Morales Viteri D (2025) *Anopheles* (Diptera: Culicidae) mosquito species in Ecuador: their role in malaria transmission. Journal of Medical Entomology 62: 659–666. 10.1093/jme/tjaf015 40176310

[B148] Quiñones ML, Ruiz F, Calle DA, Harbach RE, Erazo HF, Linton Y-M (2006) Incrimination of *Anopheles* (Nyssorhynchus) *rangeli* and An. (Nys.) oswaldoi as natural vectors of *Plasmodium vivax* in Southern Colombia. Memórias do Instituto Oswaldo Cruz 101: 617–623. 10.1590/S0074-02762006000600007 17072473

[B149] Ramírez-Aguilar N (1953) Especie Anofelina predominante en el Oriente ecuatoriano. Boletin de la oficina sanitaria Panamericana 35: 499–504. 13105785

[B150] Reinert JF (2001) Revised list of abbreviations for genera and subgenera of Culicidae (diptera) and notes on generic and subgeneric changes. Journal of the American Mosquito Control Association 17: 51–55. 11345419

[B151] Reinert JF (2009) List of abbreviations for currently valid generic-level taxa in family Culicidae (Diptera). European Mosquito Bulletin 27: 68–76.

[B152] Richards SL, Anderson SL, Lord CC, Tabachnick WJ (2012) Effects of Virus Dose and Extrinsic Incubation Temperature on Vector Competence of *Culex nigripalpus* (Diptera: Culicidae) for St. Louis Encephalitis Virus. Journal of Medical Entomology 49: 1502–1506. 10.1603/ME12054 PMC3546324 23270182

[B153] Rivas F, Diaz LA, Cardenas VM, Daza E, Bruzon L, Alcala A, la Hoz OD, Caceres FM, Aristizabal G, Martinez JW, Revelo D, la Hoz FD, Boshell J, Camacho T, Calderon L, Olano VA, Villarreal LI, Roselli D, Alvarez G, Ludwig G, Tsai T (1997) Epidemic Venezuelan Equine Encephalitis in La Guajira, Colombia, 1995. The Journal of Infectious Diseases 175: 828–832. 10.1086/513978 9086137

[B154] Rothman SE, Jones JA, LaDeau SL, Leisnham PT (2021) Higher West Nile Virus Infection in *Aedes albopictus* (Diptera: Culicidae) and *Culex* (Diptera: Culicidae) Mosquitoes From Lower Income Neighborhoods in Urban Baltimore, MD. Andreadis T (Ed.) Journal of Medical Entomology 58: 1424–1428. 10.1093/jme/tjaa262 33257956

[B155] Ruiz-Lopez F, Wilkerson RC, Ponsonby DJ, Herrera M, Sallum MAM, Velez ID, Quiñones ML, Flores-Mendoza C, Chadee DD, Alarcon J, Alarcon-Ormasa J, Linton Y-M (2013) Systematics of the *Oswaldoi Complex* (*Anopheles*, *Nyssorhynchus*) in South America. Parasites & Vectors 6: 324. 10.1186/1756-3305-6-324 PMC3843595 24499562

[B156] Rutledge CR, Day JF, Lord CC, Stark LM, Tabachnick WJ (2003) West Nile Virus Infection Rates in *Culex nigripalpus* (Diptera: Culicidae) Do Not Reflect Transmission Rates in Florida. Journal of Medical Entomology 40: 253–258. 10.1603/0022-2585-40.3.253 12943101

[B157] Sallum MAM, Schultz TR, Foster PG, Aronstein K, Wirtz RA, Wilkerson RC (2002) Phylogeny of Anophelinae (Diptera: Culicidae) based on nuclear ribosomal and mitochondrial DNA sequences. Systematic Entomology 27: 361–382. 10.1046/j.1365-3113.2002.00182.x

[B158] Saredy JJ, Chim FY, Lyski ZL, Ahearn YP, Bowers DF (2020) Confocal Analysis of the Distribution and Persistence of Sindbis Virus (TaV-GFP) Infection in Midguts of *Aedes aegypti* Mosquitoes. Microscopy and Microanalysis 26: 267–274. 10.1017/S1431927620001270 32189602

[B159] Scherer WF, Dickerman RW, La Fiandra RP, Chia CW, Terrian J (1971) Ecologic Studies of Venezuelan Encephalitis Virus in Southeastern México. The American Journal of Tropical Medicine and Hygiene 20: 980–988. 10.4269/ajtmh.1971.20.980 5131697

[B160] Schick R (1970) Mosquito studies (Diptera, Culicidae) XX. The terrens group of *Aedes* (Finlaya). Contributions of the American Entomological Institute (Ann Arbor) 5: 1–158.

[B161] Siers S, Merkel J, Bataille A, Vargas FH, Parker PG (2010) Ecological Correlates of Microfilariae Prevalence in Endangered Galápagos Birds. Journal of Parasitology 96: 259–272. 10.1645/GE-2070.1 19954259

[B162] Sippy R, Lippi C, Stewart A, Ryan S (2020) Endemic and Emerging Arboviruses of Mosquitoes in Ecuador. Práctica Familiar Rural 5. 10.23936/pfr.v5i2.165

[B163] Sirivanakarn S, Galindo P (1980) *Culex* (Melanoconion) adamesi, a New Species from Panama (Diptera, Culicidae). Mosquito Systematics 12. 10.5281/zenodo.15939475

[B164] Stone A (1967) A Synoptic Catalog of the Mosquitoes of the World, Supplement III (Diptera: Culicidae). Proceedings of the Entomological Society of Washington 69: 197–224.

[B165] Stone A (1970) A Synoptic Catalog of the Mosquitoes of the World, Supplement IV (Diptera: Culicidae). Proceedings of the Entomological Society of Washington 72: 137–171.

[B166] Stone A, Knight KL (1956) Type specimens of mosquitoes in the United States National Museum: II, The genus *Aedes* (Diptera, Culicidae). Journal of the Washington Academy of Science 46: 213–228.

[B167] Stone A, Knight K, Starcke H (1959) A Synoptic Catalog of the Mosquitoes of the World (Diptera, Culicidae). Entomological Society of America, Press Thomas Say Foundation, Washington D.C. 10.4182/umjb2446.1959.9

[B168] Taira K, Toma T, Tamashiro M, Miyagi I (2012) DNA barcoding for identification of mosquitoes (Diptera: Culicidae) from the Ryukyu Archipelago, Japan. Medical Entomology and Zoology 63: 289–306. 10.7601/mez.63.289

[B169] Tatem AJ, Huang Z, Das A, Qi Q, Roth J, Qiu Y (2012) Air travel and vector-borne disease movement. Parasitology 139: 1816–1830. 10.1017/S0031182012000352 22444826

[B170] Telles-de-Deus J, Guimarães LDO, Rocha EC, Helfstein VC, Reginato SL, Mucci LF, Bergo ES, De Camargo-Neves VLF, Kirchgatter K (2024) COI DNA barcoding to differentiate *Haemagogus janthinomys* and *Haemagogus capricornii* (Diptera: Culicidae) mosquitoes. Acta Tropica 259: 107377. 10.1016/j.actatropica.2024.107377 39245155

[B171] Turell MJ, Barth J, Coleman RE (1999) Potential for Central American mosquitoes to transmit epizootic and enzootic strains of Venezuelan equine encephalitis virus. Journal of the American Mosquito Control Association 15: 295–298. 10480118

[B172] Valderrama L, Ayala S, Reyes C, González CR (2021) Modeling the Potential Distribution of the Malaria Vector *Anopheles* (Ano.) pseudopunctipennis Theobald (Diptera: Culicidae) in Arid Regions of Northern Chile. Frontiers in Public Health 9: 611152. 10.3389/fpubh.2021.611152 PMC8144306 34046385

[B173] Valencia J (1973) Mosquito studies (Diptera, Culicidae) XXXI. A revision of the subgenus *Carrolia* of *Culex*. Contribution of the American Entomological Institute 9: 1–134.

[B174] Velásquez G (2014) Bionomics, Ecology and Medical Importance of Coquillettidia (Rhynchotaenia) venezuelensis Theobald, 1912 (Diptera: Culicidae). Saber, Universidad de Oriente 26.

[B175] Weaver SC, Salas R, Rico-Hesse R, Ludwig GV, Oberste MS, Boshell J, Tesh RB (1996) Re-emergence of epidemic Venezuelan equine encephalomyelitis in South America. The Lancet 348: 436–440. 10.1016/S0140-6736(96)02275-1 8709783

[B176] Weaver SC, Ferro C, Barrera R, Boshell J, Navarro J-C (2004) Venezuelan Equine Encephalitis. Annual Review of Entomology 49: 141–174. 10.1146/annurev.ento.49.061802.123422 14651460

[B177] Whiteman NK, Goodman SJ, Sinclair BJ, Walsh T, Cunningham AA, Kramer LD, Parker PG (2005) Establishment of the avian disease vector *Culex quinquefasciatus* Say, 1823 (Diptera: Culicidae) on the Galápagos Islands, Ecuador. Ibis 147: 844–847. 10.1111/j.1474-919X.2005.00468.x

[B178] Wilkerson R, Sallum MA, Forattini OP (1997) Redescription of Anopheles (Anopheles) shannoni Davis; a member of the Arribalzagia series from the amazon basin (Diptera: Culicidae). Proceedings of the entomological society of Washington 99: 461–471.

[B179] World Population Review (2025) Mosquito Population by Country 2025. https://worldpopulationreview.com/country-rankings/mosquito-population-by-country [accessed June 5, 2025].

[B180] Yao H, Song J, Liu C, Luo K, Han J, Li Y, Pang X, Xu H, Zhu Y, Xiao P, Chen S (2010) Use of ITS2 Region as the Universal DNA Barcode for Plants and Animals. Hansson B (Ed.). PLoS ONE 5: e13102. 10.1371/journal.pone.0013102 PMC2948509 20957043

[B181] Zavortink TJ (1973) Mosquito Studies (Diptera, Culicidae) XXIX. A Review of The Subgenus *Kerteszia* of *Anopheles*. Contributions of the American Entomological Institute 9: 1–56.

[B182] Zavortink TJ (1979) Mosquito Studies (Diptera, Culicidae) XXXV. The New Sabethine Genus *Johnbelkinia* and A Preliminary Reclassification Of The Composite Genus *Trichoprosopon*. Contributions of the American Entomological Institute 17: 1–61.

