# Supplementary material for: An updated checklist of mosquitoes (Diptera, Culicidae) of Ecuador: new records and public health significance
Source: Zookeys. 2026 Mar 5;1272:67–136. doi: 10.3897/zookeys.1272.179156 (PMC12983083; doi:10.3897/zookeys.1272.179156)
Supplement: Supplementary material 2 — Classification of mosquito species by vectorial evidence [file zookeys-1272-067_article-179156__-s002.docx]

**Supplementary 2.** Classification of mosquito species by vectorial evidence

| **Specie** | **Pathogen(s) Reported** | **Transmission Role** | **Reference** |
| --- | --- | --- | --- |
| *Anopheles fluminensis* | *Plasmodium spp.* | Confirmed vector | (Neves et al. 2013) |
| *Anopheles mattogrossensis* | *Plasmodium spp.* | Parasite detected in blood meal—vector role remains uncertain | (Pimenta et al. 2015) |
| *Anopheles neomaculipalpus* | *Plasmodium spp.* | Evidence of natural infection —secondary vector | (Moreno et al. 2005) |
| *Anopheles calderoni* | *Plasmodium spp.* | Confirmed vector | (Naranjo-Díaz and Correa 2025) |
| *Anopheles pseudopunctipennis* | *Plasmodium spp.* | Confirmed vector | (Naranjo-Díaz and Correa 2025; Valderrama et al. 2021) |
| *Anopheles punctimacula* | *Plasmodium spp.* | Confirmed vector | (Naranjo-Díaz and Correa 2025) |
| *Anopheles vestitipennis* | *Plasmodium spp.* | Parasite detected in blood meal—vector role remains uncertain | (Loyola et al. 1991) |
| *Anopheles* cruzii | *Plasmodium spp.* | Confirmed vector | (Lorenz et al. 2012) |
| *Anopheles pholidotus* | *Plasmodium spp.* | Confirmed vector | (Escovar et al. 2014; Naranjo-Díaz and Correa 2025) |
| *Anopheles neivai* | *Plasmodium spp.* | Confirmed vector | (Naranjo-Díaz and Correa 2025) |
| *Anopheles marajoara* | *Plasmodium falciparum* | Evidence of natural infection —secondary vector | (Brochero et al. 2010) |
|  | *Plasmodium vivax* | Evidence of experimental infection —secondary vector |  |
|  | *Wuchereria bancrofti* | Possible vector (unconfirmed transmission) |  |
| *Anopheles nuneztovari* | *Plasmodium spp.* | Confirmed vector | (Naranjo-Díaz and Correa 2025) |
| *Anopheles homunculus* | *Plasmodium spp.* | Secondary vector | (Lorenz et al. 2012) |
| *Anopheles oswaldoi* | *Plasmodium spp.* | Parasite detected in blood meal—vector role remains uncertain | (Pimenta et al. 2015) |
| *Anopheles benarrochi* | *Plasmodium vivax* | Evidence of natural and experimental infection—low experimental transmission rate | (Pereira-Silva et al. 2022) |
| *Anopheles albimanus* | *Plasmodium spp.* | Confirmed vector | (Naranjo-Díaz and Correa 2025) |
| *Anopheles aquasalis* | *Plasmodium spp.* | Confirmed vector | (Alencar et al. 2023) |
| *Anopheles darlingi* | *Plasmodium spp.* | Confirmed vector | (Naranjo-Díaz and Correa 2025; Pontual et al. 2025) |
| *Anopheles evansae* | *Plasmodium vivax* | Evidence of experimental infection — low experimental transmission rate | (Pereira-Silva et al. 2022) |
| *Anopheles rangeli* | *Plasmodium vivax* | Evidence of natural infection—it is necessary to assess the role of *An. rangeli* in malaria transmission across Latin America | (Quiñones et al. 2006) |
| *Anopheles triannulatus* | *Plasmodium vivax* | Parasite detected in blood meal—vector role remains uncertain | (Pimenta et al. 2015) |
|  | *Plasmodium falciparum* |  |  |
| *Anopheles trinkae* | *Plasmodium spp.* | Recognized as an important malaria vector based on field evidence (salivary gland dissections, abundance, and human-vector contact rates) | (Hayes et al. 1987) |
| *Aedeomyia squamipennis* | GAMV | Confirmed vector | (Burkett-Cadena and Blosser 2017) |
|  | Orthobunyavirus of  the Gamboa serogroup |  |  |
|  | *Plasmodium spp.* (Avian malaria) |  |  |
|  | VEEV | Isolated from field-collected females |  |
| *Aedes aegypti* | CHIKV | Confirmed vector | (Cevallos et al. 2018; Ogunlade et al. 2021) |
|  | YFV |  |  |
|  | All serotypes of DENV |  |  |
|  | ZIKV |  |  |
|  | SINV | Evidence of experimental infection | (Saredy et al. 2020) |
|  | *Wuchereria bancrofti* | Evidence of experimental infection | (Paily et al. 2006) |
| *Aedes albopictus* | USUV | Evidence of natural infection | (Mitchell et al. 1992) |
|  | KSV |  |  |
|  | TENV |  |  |
|  | CVV |  | (Mitchell et al. 1998) |
|  | POTV | Evidence of natural and experimental infection | (Mitchell et al. 1992; Puggioli et al. 2017; Rothman et al. 2021) |
|  | EEEV |  |  |
|  | WNV |  |  |
|  | VEEV |  |  |
|  | YFV | Confirmed vector | (Ferreira-de-Lima and Lima-Camara 2018; Grard et al. 2014) |
|  | CHIKV |  |  |
|  | ZIKV |  |  |
|  | All serotypes of DENV |  |  |
|  | MAYV | Evidence of experimental infection | (Pereira-dos-Santos et al. 2020) |
|  | RRV |  |  |
|  | SINV |  |  |
|  | WEEV |  |  |
|  | JCV |  |  |
|  | KSV |  |  |
|  | LACV |  |  |
|  | RVFV |  |  |
|  | *Dirofilaria immitis* | Evidence of natural and experimental infection | (Cancrini et al. 2003) |
| *Aedes taeniorhynchus* | VEEV | Confirmed vector | (Rivas et al. 1997) |
|  | WNV | Evidence of experimental infection | (Eastwood et al. 2013) |
| *Haemagogus leucocelaenus* | YFV | Confirmed vector | (Li et al. 2022) |
| *Haemagogus janthinomys* | YFV | Confirmed vector | (Li et al. 2022) |
| *Haemagogus anastasionis* | YFV | Suggested as a potential vector | (Navarro et al. 2013a) |
|  | MAYV |  |  |
| *Psorophora ferox* | ROCV | Confirmed vector | (De Souza Lopes et al. 1981) |
|  | ILHV |  | (Johnson et al. 2007). |
| *Culex quinquefasciatus* | *Plasmodium spp.* (Avian malaria) | Confirmed vector | (Whiteman et al. 2005) |
|  | Avian pox virus |  |  |
|  | WNV |  |  |
|  | OROV | Secondary vector | (Consoli and Lourenço-de-Oliveira 1994) |
| *Culex nigripalpus* | WNV | Confirmed vector | (Rutledge et al. 2003) |
|  | SLEV |  |  |
|  | EEEV |  |  |
|  | VEEV |  | (De Carvalho et al. 2017) |
| *Culex* *iolambdis* | VEEV | Suggested as a vector | (Scherer et al. 1971) |
| *Culex ocossa* | Flavivirus | Confirmed vector | (Evangelista et al. 2013) |
|  | VEEV |  | (Morrison et al. 2008) |
| *Deinocerites pseudes* | VEEV | Suggested as a potential vector | (Adames 1971) |
|  | SLEV |  |  |
| *Coquillettidia albicosta* | RBUV | Confirmed vector | (Mahy and Van Regenmortel 2008) |
| *Coquillettidia venezuelensis* | MAYV | Suggested as a potential vector | (Velásquez 2014) |
|  | OROV |  |  |
|  | SLEV |  |  |
| *Mansonia titillans* | VEEV | Evidence of natural infection—secondary vector | (Morrison et al. 2008; Hoyos-López et al. 2015; Weaver et al., 2004) |
|  | SLEV | Evidence of natural infection |  |
|  | *Dermatobia hominis* eggs | Transporter | (Hervé et al. 1986; Lourenço-de-Oliveira and Heyden 1986) |
|  | Bunyamwera serogroup | Evidence of natural infection | (Maes et al. 2018) |
|  | *Plasmodium spp.* (Avian malaria) |  | (Ferreira et al. 2016) |
| *Mansonia* *pseudotitillans* | *Plasmodium spp.* (Avian malaria) | Evidence of natural infection | (Ferreira et al. 2016) |
| *Mansonia indubitans* | VEEV | Evidence of natural infection —secondary vector | (Turell et al. 1999) |
| *Johnbelkinia ulopus* | TNTV | Evidence of natural infection | (Zavortink 1979) |
| *Sabethes chloropterus* | YFV | Confirmed vector | (De Rodaniche and Galindo 1957) |
|  | SLEV | Evidence of natural infection | (Harbach 1994) |
|  | ILHV |  | (De Rodaniche and Galindo 1957) |
| *Sabethes amazonicus* | YFV | Suggested as a potential vector | (Navarro et al. 2013a) |
| *Sabethes albiprivus* | YFV | Evidence of natural infection | (Cano et al. 2021) |
